# Age-related symptom clustering in pediatric hypermobility spectrum disorders: a scoping review

**DOI:** 10.1186/s13023-026-04424-0

**Published:** 2026-05-30

**Authors:** Phoebe Pochcial, Sarah Cohen Solomon, Katelyn A. Bruno, DeLisa Fairweather, Angelica Kinane, Lisa Letzkus, Kelly K. Gurka, Ina Stephens, Dacre R. T. Knight

**Affiliations:** 1 Wroclaw Medical University, Wroclaw, Poland; 2 Department of Pediatrics, University of Virginia, Charlottesville, VA USA; 3 Division of Cardiovascular Medicine, Department of Medicine, University of Florida, Gainesville, FL USA; 4 Department of Medicine, University of Virginia, Charlottesville, VA USA; 5 Department of Cardiovascular Medicine, Mayo Clinic, Jacksonville, FL USA; 6 Center for Clinical and Translational Sciences, Mayo Clinic, Rochester, MN USA

**Keywords:** Hypermobile ehlers-danlos syndrome, Pediatric hypermobility, Early diagnosis, Scoping review, Neurodevelopmental comorbidities, Diagnostic delay, Hypermobility spectrum disorder

## Abstract

**Background:**

Pediatric generalized hypermobility spectrum disorders (pgHSD), are a group of multisystemic heritable connective tissue disorders frequently under-recognized in pediatric populations. The variable and often vague early symptomatology, coupled with the absence of definitive genetic markers, present significant diagnostic challenges. Early manifestations are commonly misattributed to other conditions, leading to substantial diagnostic delays and prolonged morbidity. The purpose of this study is to provide clarity on the presentation of pgHSD symptoms that may ultimately reduce diagnostic delays.

**Methods:**

A scoping review was conducted in accordance with the PRISMA-ScR guidelines. Comprehensive searches were performed on studies reporting clinical features of patients aged 0–18 years diagnosed with hypermobile Ehlers-Danlos syndrome (hEDS), pgHSD, and joint hypermobility syndrome (JHS). There were 27 studies that met the inclusion criteria searched 1974 to May 8, 2025.

**Results:**

Thematic analysis identified eight recurring symptom clusters that frequently characterize early pgHSD. The most consistently reported features included frequent subluxations or dislocations, chronic musculoskeletal pain, disabling fatigue, orthostatic intolerance, and various gastrointestinal complaints. Notably, impaired neurodevelopmental traits were frequently reported.

**Conclusions:**

This scoping review maps age-related symptom/comorbidity clusters reported in pediatric hypermobility spectrum disorders, with the aim of informing earlier clinical recognition.

**Supplementary Information:**

The online version contains supplementary material available at 10.1186/s13023-026-04424-0.

## Introduction

Ehlers-Danlos syndromes (EDS) comprise a heterogeneous group of inherited connective tissue disorders characterized by joint hypermobility, skin hyperextensibility, and tissue fragility [1–4]. The most common hypermobility-related conditions include hypermobility spectrum disorders (HSD), encompassing pediatric generalized hypermobility spectrum disorder (pgHSD), and hypermobile Ehlers-Danlos syndrome (hEDS), which is reserved for biologically mature adolescents [4–6]. Diagnosis of these conditions in childhood remains challenging due to the absence of a definitive biomarker and a highly variable, multisystem clinical presentation [1, 2, 5]. As a result, diagnostic delays are common – over a decade on average – and are associated with substantial morbidity [1, 3, 5, 7–9].

Children with hEDS/pgHSD frequently present with symptoms extending beyond the musculoskeletal system, reflecting the systemic nature of connective tissue involvement. Common manifestations include chronic pain, fatigue, autonomic dysfunction (e.g., orthostatic intolerance), gastrointestinal disturbances, and a higher prevalence of neurodevelopmental delay [1, 3, 6–9]. These symptoms are often evaluated in isolation or attributed to more common pediatric conditions, delaying recognition of an underlying connective tissue disorder [8, 9].

Historically, pediatric diagnosis has been complicated by the application of adult-derived criteria. The 2017 hEDS diagnostic framework posed particular challenges in children, as many defining features evolve with age and may not be fully expressed in early life [4, 8, 10–12]. Importantly, the average age that females and males mature sexually differs significantly, resulting in an earlier progression to pgHSD in females. Additionally, while the Beighton score is useful for identifying generalized joint hypermobility, it does not reliably distinguish benign hypermobility from pathological connective tissue disorders in pediatric populations [11].

To address these limitations, a pediatric-specific diagnostic framework was introduced in 2023 [12]. This framework classifies joint hypermobility from age five through biological maturity and emphasizes a dynamic diagnostic approach, distinguishing generalized joint hypermobility (GJH) from pgHSD based on musculoskeletal complications, skin and tissue findings, and core comorbidities. Importantly, it recognizes that diagnostic categories may evolve over time. Despite this advance, awareness and consistent clinical application of the framework remain limited, and pgHSD continues to present with age-dependent symptom patterns that frequently mimic common pediatric complaints [9, 11].

Given the heterogeneity of clinical presentations, evolving terminology, and variability in study designs, a scoping review approach was selected to comprehensively map the existing literature. The aim of this review was to characterize early, age-related symptom patterns and recurring multisystem clusters reported in pediatric hEDS and HSD. By synthesizing these patterns, this work seeks to support earlier clinical recognition and provide a foundation for the future development of pediatric screening approaches, with the ultimate goal of improving outcomes for affected children.

## Methods

### Protocol and reporting standards

This scoping review was conducted in accordance with the Preferred Reporting Items for Systematic Reviews and Meta-Analyses extension for Scoping Reviews (PRISMA-ScR) guidelines [13]. A predefined protocol guided the review process, including the review questions, eligibility criteria, search strategy, study selection, and data charting methods.

A scoping review methodology was selected due to the heterogeneity of study designs, evolving diagnostic terminology, and variability in reported outcomes within the pediatric hypermobility literature. This approach was chosen to comprehensively map existing evidence rather than to assess intervention efficacy or generate pooled estimates.

### Eligibility criteria

Studies were eligible for inclusion if they reported clinical features, early symptoms, comorbidities, or functional impacts in pediatric populations aged 0–18 years. Longitudinal studies were included if pediatric-onset data were reported, even when follow-up extended into adulthood.

Eligible studies included those using the diagnostic labels hypermobile Ehlers-Danlos syndrome (hEDS), hypermobility spectrum disorder (HSD), joint hypermobility syndrome (JHS), or the more recent pediatric generalized hypermobility spectrum disorder (pgHSD). Inclusion of multiple diagnostic terms reflected historical variability in nomenclature and recognized symptom overlap. Interpretation of findings was guided by the 2023 pediatric diagnostic framework for joint hypermobility [11].

Included studies were limited to human research published in English and employed observational, descriptive, or mixed-methods designs. Exclusion criteria included studies focused exclusively on adult populations without pediatric-onset data, non-peer reviewed abstracts, non-peer reviewed gray literature, case reports of only one patient, reports limited to genetic or molecular findings without reports of symptomology, and studies examining biomechanics alone without patient-reported or clinical symptom outcome data.

### Information sources and search strategy

A comprehensive literature search was conducted January 1, 1974 through May 8, 2025. To ensure broad coverage, three complementary approaches were used to identify relevant studies. All searches and study selection procedures were conducted by a single reviewer (PP).

### AI-assisted semantic search

An artificial intelligence-assisted semantic search was conducted using Elicit.org, an AI-powered research discovery tool drawing primarily from the Semantic Scholar corpus. The research question, “*What are symptoms or early signs of hypermobile Ehlers–Danlos syndrome in children?*”, was used to generate an initial list of 50 semantically relevant articles.

All articles identified through Elicit underwent manual title, abstract, and full-text screening using the same eligibility criteria applied to bibliographic database searches. Elicit’s internal relevance rankings were not used for study inclusion decisions. Articles ultimately included from this search were independently verified and accessed through conventional bibliographic databases (e.g., PubMed). The Elicit search was treated as a supplementary discovery method and is reported as a distinct source in the PRISMA-ScR flow diagram.

### Bibliographic database searching

Traditional bibliographic database searches were conducted in PubMed and Embase. PubMed was searched using Boolean combinations of Medical Subject Headings (MeSH) and free-text terms related to Ehlers-Danlos syndromes, pediatric populations, and clinical presentation. The full PubMed search strategy is provided in Appendix A.

Embase was searched via Ovid (1974 to May 8, 2025) using a strategy developed and executed by an experienced health sciences librarian, incorporating Emtree terms and keywords related to pediatric hypermobility disorders (i.e., joint hypermobility, generalized joint hypermobility, hypermobility spectrum disorder, pediatric hypermobility spectrum disorder, Ehlers-Danlos syndrome, hypermobile Ehlers-Danlos syndrome, benign joint hypermobility syndrome, heritable disorders of connective tissue). The complete Embase search strategy is available in Supplementary File 2. All database searches were limited to English-language human studies where filters permitted.

**Fig. 1:**
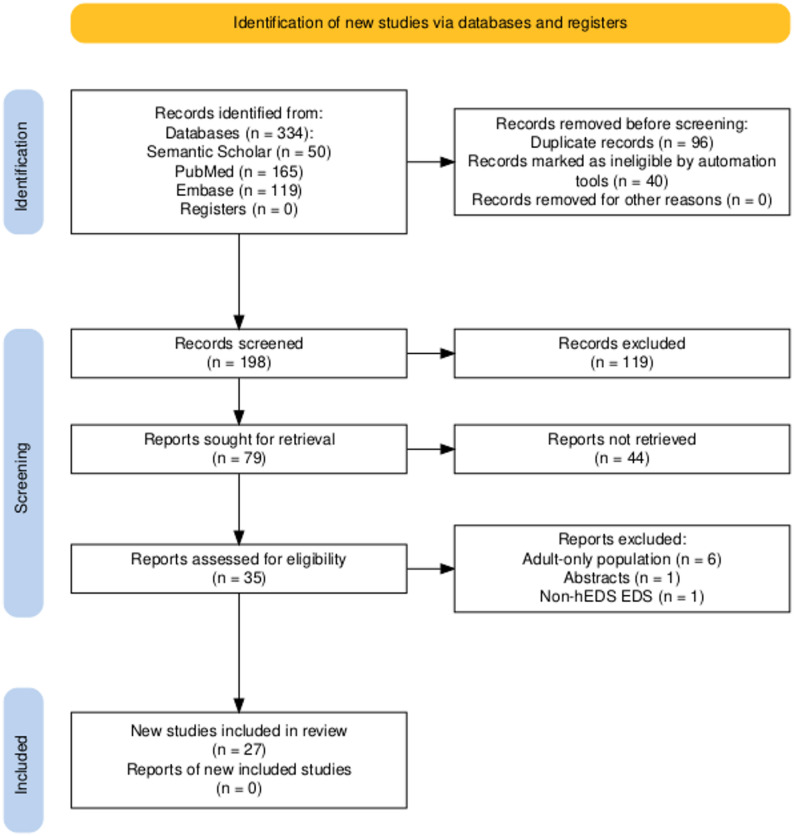
PRISMA-ScR flowchart illustrating identification, screening, and inclusion of studies examining pediatric hypermobility spectrum disorders. Studies using legacy diagnostic terminology (e.g., benign joint hypermobility syndrome, and hEDS vs. pgHSD) were retained when pediatric-onset hypermobility was clearly described. Automation tools were used for preliminary exclusion of clearly irrelevant records prior to manual screening. Reports not retrieved were primarily abstracts or inaccessible full text

### Selection of sources of evidence

The study selection process is summarized in the Fig. 1 PRISMA-ScR flow diagram [14]. Records were managed using Zotero reference management software. After automatic duplicate removal (*n* = 96) and automated identification of clearly ineligible records (*n* = 40), 198 titles and abstracts were screened. Full texts were retrieved for 79 articles and assessed for eligibility against predefined criteria.

Of these, 44 full-text reports were irretrievable despite reasonable efforts. Four additional studies were excluded after full-text review: three due to exclusive focus on adult populations and one due to primary emphasis on non-hypermobile EDS subtypes. A total of 27 studies were included in the final synthesis. All screening and selection steps were performed by a single reviewer, which may introduce selection bias. This limitation is addressed in the Discussion.

### Data charting process

Data were extracted using a standardized data charting form developed in Zotero. A blank version of the charting form is provided in Supplementary File 1. Extracted variables included study design, sample size, age range, demographic characteristics, early symptom profiles, symptom prevalence, age at onset, diagnostic delay, comorbidities, functional limitations, psychosocial impact, health-related quality of life, diagnostic considerations, reported “red flag” features, and management or intervention approaches. To promote consistency, periodic automated re-extraction and cross-checking of randomly selected studies were performed. Extracted data were organized and synthesized using Zotero and Microsoft Excel.

### Critical appraisal of individual sources of evidence

Consistent with scoping review objectives to map available literature, formal critical appraisal of study quality or risk of bias was not undertaken. The review mapped reported clinical presentations without evaluating evidence strength or intervention effectiveness.

### Synthesis of results

A descriptive numerical summary and thematic synthesis were employed. Quantitative data (e.g., symptom frequency, age of onset, diagnostic delay, comorbidity prevalence) were summarized descriptively. Qualitative data relating to clinical symptom presentations, age-related symptom evolution, functional impact, and diagnostic experiences were analyzed using an inductive thematic approach involving familiarization, coding, and theme development. Themes were subsequently grouped by physiological systems and recurring symptom clusters to provide an integrated overview of pediatric hEDS and pgHSD presentations. Thematic clustering also accounted for study heterogeneity and minimized single-domain bias.

## Results

### Study characteristics

This scoping review embarked on a vital exploration of pediatric hypermobility spectrum disorders (pgHSD), synthesizing insights from 27 studies. As detailed in the PRISMA-ScR flow diagram (Fig. 1), the breadth of evidence encompassed diverse methodologies, from retrospective chart reviews [5, 8, 15] offering a historical lens to observational case-control studies [7] and cross-sectional studies [16–19] capturing snapshots of current presentation. Table 1 provides a comprehensive overview of the 27 studies. Many of the studies focused on a particular set of symptoms; however, several studies (*n* = 7) examined a more comprehensive range of symptoms which were useful for creating a more consistent symptom profile. Most studies included pediatric patients that spanned the crucial developmental window from infancy to 18 years [9, 18, 20–23], with data extending into early adulthood (up to 28 years, e.g. [17]).

Sample sizes varied, ranging from a small study of hEDS/HSD patients (*n* = 12) (de Koning 2023) to expansive retrospective cohorts involving over 400 participants [17]. A female predominance was consistently observed across cohorts, typically comprising 66% [17, 24] to 89% [14, 25] of patients, with a majority of Caucasian/White individuals (89-95.5%) [4, 17, 24]. Mean ages of the pediatric and adolescent cohorts generally fell between 11.0 and 16.0 years of age [5, 14, 25].

**Table 1:** Studies included in the Scoping review

Study	Study design	Sample size (*n*)	Age range (years)	Main symptom findings
Baeza-Velasco 2017 [9]	Narrative review	104 papers	pediatric	behavioral, feeding difficulties, hyper/hypoactivity, inattention, language, mood issues, motor abnormality, sleep, social interaction
Coles 2018 [6]	Narrative review	-	pediatric	anxiety, bladder dysfunction, depression, DGBI, musculoskeletal pain, pelvic dysfunction, POTS
de Koning 2023 [16]	Cross-sectional study examining gait	12 hEDS/ HSD, 15 healthy controls	12–18	gait issues
de Koning 2023 [18]	Observational, multicenter study to examine somatic symptoms, pain and pain catastrophizing	127 heritable connective tissue disorder (29 hEDS)	4–18	functional disability, nonspecific somatic symptoms, pain, pain catastrophizing
Feldman 2020 [10]	Narrative review of pain symptoms	-	pediatric	musculoskeletal pain, other pain (i.e., headache, abdominal pain, dysmenorrhea, painful pedal papules)
Feldman 2023 [1]	Narrative review on illness uncertainty in hEDS	7 pediatric/ 35 studies	includes pediatric	discusses symptoms of illness
Garcia 2025 [5]	Retrospective chart review on joint pain & strength	69	10–22	joint pain (i.e., knee, hip, shoulder, back, ankle, wrist pain)
Gensemer 2021 [19]	Narrative review	-	includes pediatric	delay in walking, clumsiness, musculoskeletal pain, other pain, sleep issues
Hertel 2024 [4]	Mini review of cardiovascular symptoms in hEDS	-	includes pediatric	cardiovascular symptoms (i.e., lightheadedness, palpitations, fainting, chest pain), dysautonomia
Hertel 2024 [17]	Longitudinal study of cardiovascular symptoms in hEDS	70	5–22	chest pain, dizziness, dysautonomia, orthostatic intolerance, palpitations, postural orthostatic tachycardia syndrome (POTS), shortness of breath tachycardia
Hertel 2025 [20]	Longitudinal study of psychiatric & sleep disorders in hEDS	123	5–22	anxiety, depression, poor sleep
Jeong 2023 [21]	Mixed-methods, cross-sectional study	21 hEDS/ HSD	8–17	functional disability, pain interference, sleep difficulties
Jones 2022 [22]	Survey of families with EDS	238 (83 minors)	Includes pediatric	rheumatology, cardiology, and neurology most referrals for pediatric patients
Kindgren 2021 [23]	Retrospective study of ADHD & ASD	201 (88 hEDS, 113 HSD)	6–18	ADHD, ASD, fatigue, gastrointestinal, pain, sleep, urinary tract problems; symptoms worse if had ADHD, ASD
Lahey 2024 [24]	Retrospective study of aortic root dilation in hEDS	237	pediatric	aortic root dilation same as general population
Morris 2017 [25]	Cross-sectional analysis of GJH	1,584	14	musculoskeletal, pain (back, limb, neck), sex differences observed
Peterson 2018 [26]	Systematic review of trials for PT for lower limb symptoms	86 (2 RCTs)	7–16	lower limb symptoms, Current studies are limited by small sample sizes and high attrition rates. No PT compares to sham intervention or no intervention, so overall effectiveness is unknown.
Russek 2019 [27]	Narrative review	-	-	balance issues, delayed motor development, dysautonomia, fatigue, GI problems, pain, poor motor control, poor postural control, proprioception, stress incontinence
Scheper 2013 [28]	Narrative review + RCTs	95 (3 RCTs)	0–18	activity avoidance, fatigue, muscle strength, musculoskeletal complaints, pain, proprioception
Scheper 2017 [29]	Discriminative analysis to study generalized hyperalgesia	225 (51 children, 174 adults)	<18	generalized hyperalgesia (lowered pain threshold), chronic pain
Schubert-Hjalmarsson 2025 [7]	Prospective, case-control study to examine central sensitization	37 hEDS/ HSD, 47 healthy controls	13–17	central sensitization, generalized hyperalgesia, fatigue, musculoskeletal pain, pain pressure thresholds
Sokol 2025 [8]	Retrospective study of pain, fatigue, and anxiety	200 (100 hEDS, 100 chronic pain controls)	9–17	fatigue (associated with pain interference and avoidance)
Sood 2024 [15]	Retrospective study of GI symptoms	435 hEDS/JHS	5–28	constipation, dyspepsia, dysphagia
Tofts 2009 [11]	Narrative review	534 papers	<18	anxiety, fatigue, musculoskeletal pain, osteopenia
Tofts 2023 [12]	Narrative review	-	9–18	core comorbidities (i.e., anxiety, chronic fatigue, chronic pain, non-structural bladder disorder, disorders of gut-brain interaction, dysautonomia), musculoskeletal complications, skin and tissue abnormalities
Veriki 2021 [30]	Cross-sectional survey of bladder dysfunction	67	6–18	enuresis, urinary urgency
Yeung 2024 [31]	Retrospective study of fractures	26 EDS/GJH	< 18	fractures (result of identifiable injury event- no comparative controls)

Primary recurring symptom categories found from the Scoping review are listed in Table 2 and referred to as ‘red flag’ symptoms to assist in early identification of hEDS and pgHSD in pediatric populations. Interestingly, these symptom groupings are the same as have been reported in adults with hEDS/HSD [32–34]. For each symptom cluster, the table outlines key features reported in the literature, their clinical significance and impact on patients, common misdiagnoses, and crucial clinical pearls or diagnostic indicators to aid in earlier recognition.

**Table 2:** Pediatric HSD/ hEDS: red flag symptom clusters, clinical significance, and diagnostic pearls

Red Flag symptom cluster	Key symptoms	Clinical significance	Common misdiagnosis	Clinical Pearl
Musculo-skeletal pain & instability	Chronic pain (mean pain intensity of 5.53 on an 11-point scale [10]) in up to 89% of patients with hEDS [1] Pain in multiple joints in 94% of patients age 6–16 [11] Frequent subluxations, dislocations.[10, 12] Early motor delays, clumsiness, poor coordination, proprioception deficits [9, 10, 13, 21, 31]. Altered gait, reduced stability(16). Average pain was found in 7 body regions [10].	Leading cause of disability and reduced QoL. Often presents before age 10. [12] Associated with activity avoidance [5] and psychosocial strain. Number of studies reporting = 9.	“Growing pains”, benign hypermobility orthopedic overuse [10].	Pain > 3 months, widespread [18], or function-limiting is not normal. Hyperalgesia [31] and proprioceptive deficits [1] are key clues.
Autonomic dysfunction & orthostatic intolerance (POTS/OI)	59% OI, 21% POTS in pediatric hEDS [12]. 47% with diagnosed dysautonomia [14]. Symptoms include dizziness, palpitations [18] tachycardia, [4, 18] syncope. Pupillomotor symptoms contribute to HRQoL loss. Symptoms of autonomic dysfunction [9, 12] can also include positional hypotension, gastrointestinal dysmotility, disturbed bladder function, and sweating regulation [18]. Vasovagal dystonia [9].	Major source of fatigue, school absences, daily impairment. High autonomic burden linked to worse HRQoL. Number of studies reporting = 6.	Anxiety, panic, dehydration, vasovagal syncope, psychogenic symptoms [18].	A Stand Test can be used as a substitute for a formal tilt-table test in patients who complain of fatigue and POTS-related symptoms [13]. Consider symptomatic HSD, including pgHSD, if autonomic symptoms coexist with hypermobility.
Chronic GI issues	Constipation (61%), dysphagia (32%), dyspepsia (25%) [4]. 54% of children with hEDS with GI complaints [10]. Abnormal EGD (58%), GES (59%), EoE (21%), celiac (4%) [17].	Highly prevalent, impacts nutrition, growth, sleep, and QoL. Symptoms often resist standard treatment. Number of studies reporting = 3.	DGBI, IBS, anxiety-related GI upset, dietary issues [17].	Evaluate for coexisting fatigue, dizziness, MSK pain. Dysphagia + dysmotility suggest deeper systemic issues.
Debilitating fatigue & non-restorative sleep	Fatigue in 71% of hEDS youth [23]. Mean fatigue score 68.9 vs. 32.2 (controls) [31]. Sleep problems in 45%, with OSA (26%), insomnia (22%), PLMD (17%), hypersomnia (15%) [25]. Up to 65% of pediatric hEDS patients require pharmacologic treatment for sleep concerns [25].	Predictor of school absenteeism, functional limitation [8]. Often precedes pain or autonomic signs. Number of studies reporting = 4.	Depression, anemia, behavioral issues, laziness.	Persistent, activity-limiting fatigue unrelieved by rest suggests systemic involvement. Sleep study may reveal comorbid OSA.
Neuro-developmental & neuro-psychiatric overlap	ADHD (16–23%), ASD (5–8%) [23], anxiety (80%), depression (43%) [25]. Irrepressible fidgetiness [9] 86% of pediatric hEDS patients with ≥ 1 psychiatric diagnosis [25].	Impacts academic, social, and emotional function. ND traits may obscure physical symptoms. Number of studies reporting = 4.	ADHD, ASD, anxiety, somatization [25].	Screen for symptomatic HSD, including pgHSD, if ND symptoms coexist with pain, fatigue, GI distress, or clumsiness [9].
Bladder & pelvic floor dysfunction (early onset)	LUTD w/ urgency (97%), enuresis (42%), frequency (50.8%), voiding posture (49.2%), and holding maneuvers (52.3%), onset age ~ 3.7 years [24]. Stress incontinence in 26% [11].	Can cause embarrassment, social withdrawal. Emotional distress frequently reported. Number of studies reporting = 3.	Behavioral voiding disorder, anxiety, UTI [11].	Early LUTD with joint laxity = red flag [23]. Screen for systemic symptoms. Consider pelvic floor dysfunction even in children.
Pain, headache, & neurological findings	Widespread pain and generalized hyperalgesia (lower pressure-pain thresholds − 222 to -259%) [31]. Migraine, tension headaches, CSF leak, IIH, neuropathic pain, Chiari malformation [10].	Suggests altered pain processing and CNS involvement; requires multidisciplinary pain management. Number of studies reporting = 2.	Growing pains, anxiety, fibromyalgia, psychogenic pain [31].	Look for widespread pain without clear orthopedic cause, or pain out of proportion to exam; evaluate for neurological structural issues if symptoms suggest.
Immune dysregulation & connective tissue clues	Soft, hyperextensible skin [1], easy bruising, piezogenic papules, atrophic scars, recurrent hernias, [1] recurrent soft tissue injuries [9–11, 31], high or narrow palate [13]. MCAS-type symptoms [13] (urticaria, flushing, pruritus) [10, 12, 13].	Offers visible diagnostic clues; symptoms mimic (are) allergic or dermatologic conditions. Number of studies reporting = 5.	Isolated bruising (child abuse concern), unexplained fractures, idiopathic urticaria, allergies, eczema, asthma [10, 13].	Look for a combination of seemingly unrelated mild skin/ tissue signs; consider MCAS if there are episodic allergic-type reactions or flushing without clear triggers.

Abbreviations: ASD: Autism Spectrum Disorder. GI: Gastrointestinal. HRQoL: Health-Related Quality of Life. PLMD: Periodic Limb Movement Disorder. IU: Illness Uncertainty. OSA: Obstructive Sleep Apnea. EoE: Eosinophilic Esophagitis. ND: Nocturnal Diuresis. MCAS: Mast Cell Activation Syndrome. IIH: Idiopathic Intracranial Hypertension. LUTD: Lower Urinary Tract Dysfunction. OI: Orthostatic Intolerance. AIS: Adolescent Idiopathic Scoliosis. UTI: Urinary Tract Infection

### Clinical characterization and age-related evolution of symptom clusters

**Fig. 2:**
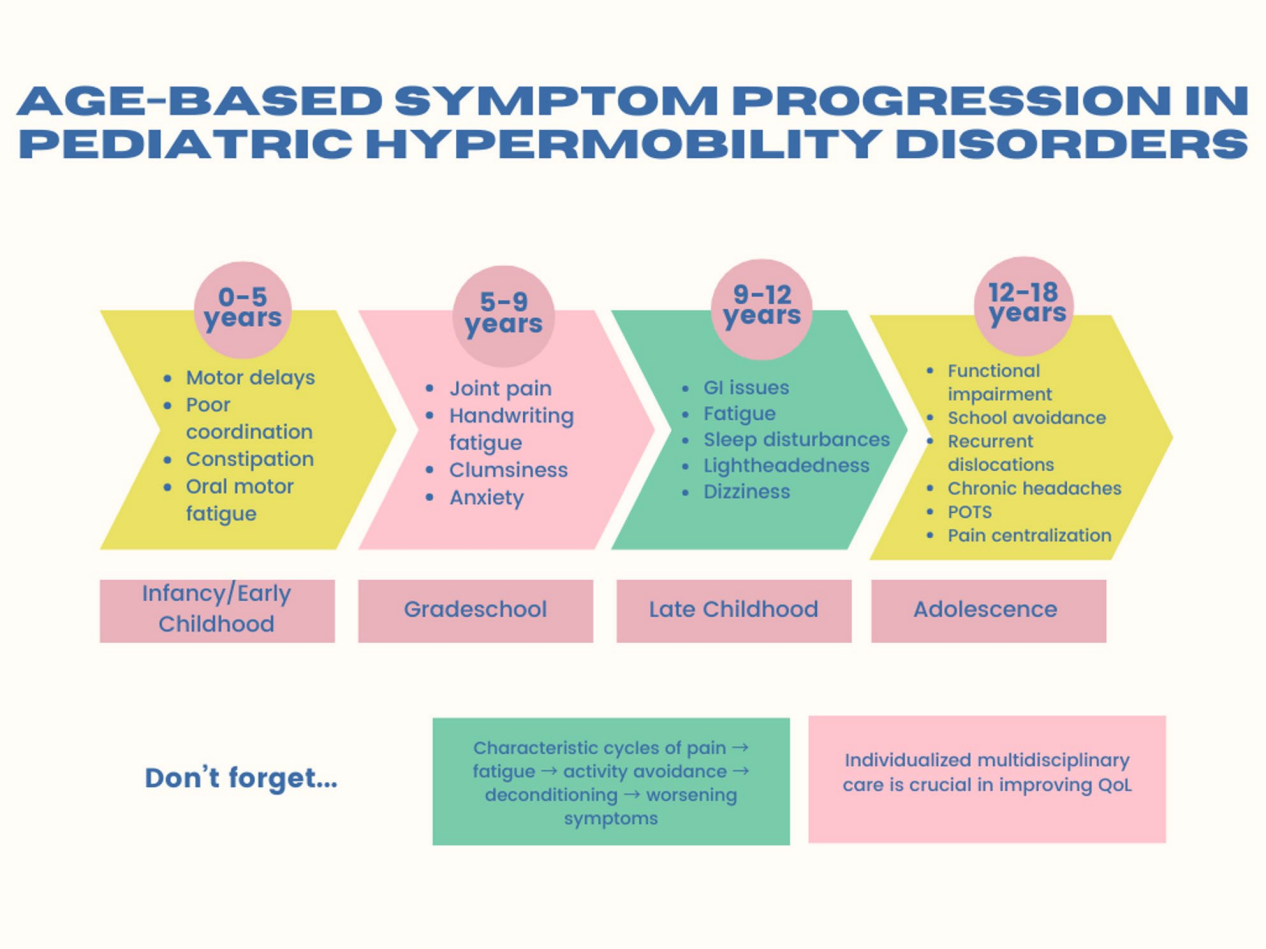
Age-related symptom evolution in pediatric hypermobility spectrum disorders

Our thematic analysis revealed not just a collection of symptoms, but eight distinct, recurring symptom clusters that form the dynamic landscape of early pediatric hEDS/pgHSD. As presented in Table 2, these clusters profoundly underscore the multisystemic nature of the disorder, often creating a perplexing clinical picture. All major clusters were identified in multiple studies, with several (e.g., pain, fatigue, GI symptoms, dysautonomia) appearing in the majority of included papers. A particularly compelling aspect of this study was the discernible, yet often overlooked, progression of common symptoms with age. Based on these studies, we propose an evolution of the 8 symptom clusters in Fig. 2. This conceptual timeline represents the typical age-related progression and clustering of symptoms that may be observed in pediatric pgHSD from infancy through adolescence. It highlights the eight key symptom categories and their approximate emergence or increasing prominence across different developmental stages, underscoring the multisystemic and evolving nature of the hEDS/pgHSD. Figure 2 should be interpreted in conjunction with Table 3 which describes detailed clinical manifestations and diagnostic considerations. Organized by age, Table 3 details the eight common symptom clusters, associated clinical “red flags,” recommended evaluation approaches, suggested interventions, and important notes or pitfalls for clinicians.

**Table 3:** Clinical presentation of symptomatic hEDS/pgHSD

Age (years)	Common symptom clusters	Clinical Red Flags	Recommended evaluation	Suggested interventions	Notes/pitfalls
Infancy (0–1)	Delayed motor milestones [9, 12, 13, 21] Hypotonia [9, 12, 21] Poor head/ neck control [12] Feeding difficulties [9]	Floppy baby appearance [12] Inconsolable crying due to discomfort [9] Reflux or feeding aversion [9, 13, 23]	• Pediatric neuromuscular exam • Observe head lag & motor tone • OT referral	• Early PT • Postural support tools • Feeding evaluation	Often misattributed to benign hypotonia or sensory delay. Feeding issues may trigger GI workup instead.
Toddlers (1–3)	Frequent falls Delayed walking or atypical gait [9, 12, 13, 21] Joint hyperextension visible [1, 10] Skin fragility, bruising [9, 10]	Reluctance to walk/ climb [9] “Clumsy” label, oral motor fatigue [9, 12, 13] Excessive bruising without trauma [6, 9]	• Beighton scoring (modified for age) • Joint ROM testing • Injury history	•PT for gait and proprioception • Protective gear for fall-prone toddlers • Pediatric PT if severe	Still often dismissed as “normal clumsiness”. Bruising misattributed to abuse or low platelets.
Early childhood (4–7)	Pain in legs (especially knees, ankles) [3, 5, 12] Chronic fatigue [7, 8, 12, 13] Slow on playground [10, 21, 31] Fatigue with exertion [10, 16, 21] Coordination issues [9, 11, 13]	Avoidance of active play [1, 7, 8, 31] Frequent “growing pains” Poor posture, handwriting fatigue [12, 13]	• Grip strength & fine motor testing • Joint stability assessment •Pain frequency diaries	• OT for grip and motor control • Core/ postural exercises •Nighttime pain management	Pain is often invalidated or minimized. Often confused with ADHD or behavioral issues due to school fatigue or motor avoidance.
Middle childhood (8–12)	Chronic musculoskeletal pain [1] Headaches [10, 13] GI issues begin (constipation, nausea) [13, 21, 23] Anxiety symptoms [9, 13, 21, 25, 31] Dislocations/ subluxations [1, 10, 12, 13]	Chronic pain [1] Frequent absences from school [8] Orthodontic issues (e.g., crowded teeth, narrow palate)	• Pediatric pain questionnaire • Psychosocial screening • Screen for orthostatic intolerance	• Begin pain reprocessing therapy • Gut-directed dietary support • CBT referral if anxiety depression symptoms emerge	Misdiagnoses increase: anxiety, fibromyalgia, IBS, migraines, etc. School avoidance may raise behavioral concerns.
Adolescence (13–18)	Full-blown dysautonomia (POTS, fainting) [4, 13, 14] Widespread chronic pain [1, 7, 11, 12] TMJ dysfunction [21] Menstrual pain [11] Depression, social withdrawal [9, 13, 21, 25]	Fatigue worsens significantly [13] Exercise intolerance [13] Avoidance of sports [1, 8, 10] Recurrent injuries [9, 31] Identity confusion due to complex symptoms [7, 8, 10, 12]	• Tilt table test • Pelvic floor assessment • Pain catastrophizing scale scoring • Hormonal workup for dysmenorrhea	•Interdisciplinary pain clinics • Cardiovascular conditioning • Hormone- gynecological consult • Myofascial therapy	This is the most commonly diagnosed age, but often after years of misattribution. Symptoms overlap with mood disorders, eating disorders, or malingering. Critical window for validation.

**From ages 0–5 years**, studies (*n* = 10) showed the earliest indications of pgHSD are often subtle and easily dismissed as benign developmental variations [5, 8–13, 21, 22, 24]. Clinicians should be alert for persistent motor issues, including pronounced clumsiness, frequent falls, and delays in achieving crucial milestones like walking, running, or stair navigation [9, 21]. Children may demonstrate unusual flexibility, adopt postures like W-sitting, or kinesiophobia [5], all of which can signal underlying joint instability. These microtraumas [10, 11, 15] and compensatory movement strategies often go undiagnosed, underscoring the need for early physical therapy focusing on proprioceptive training, core stabilization, and gross motor coordination support, as evidenced by a systematic review on physical and mechanical therapies [22]. Notably, Lower Urinary Tract Dysfunction (LUTD) can also begin remarkably early, with a mean onset age of just 3.69 years (SD 2.9) in affected children [24].

**By ages 5–9 years**, studies (*n* = 11) showed musculoskeletal pain emerges as a dominant and increasingly insistent complaint [5, 9, 10–13, 15, 16, 18, 21, 31]. Children typically describe a deep, intermittent, and activity-induced discomfort, particularly following physical education, sports, or prolonged standing [5, 10, 11, 31]. This pain frequently starts in the lower extremities (knees, ankles, hips) [5, 10, 11], but progressively becomes more widespread [5, 11, 31] and persistent with advancing age [12]. Its fluctuating nature and absence of overt inflammatory signs often lead to frustrating mislabels such as “growing pains” or even psychosomatic attributions [1]. Mean pain intensity scores (VAS 0-100) were notable, averaging 48.4 for children with HMS/EDS-HT [31]. Despite minimal objective findings, clinicians may observe subtle gait abnormalities [13, 16, 21], foot pronation, or pronounced muscle fatigue [8, 12, 13]. At this crucial stage, a targeted Beighton score assessment [1, 6, 13, 15], evaluation for muscle deconditioning [8, 31], and individualized strengthening with joint protection strategies are paramount [22].

**From ages 9–12 years**, studies (*n* = 10) showed the symptom canvas expands significantly to include disorder of gut-brain interaction (DGBI) complaints (e.g., chronic constipation, bothersome reflux, abdominal pain) and a profound, debilitating fatigue [4, 8, 10–14, 17, 23, 25]. This fatigue is often non-restorative and dramatically impacts school participation [10]. It frequently co-occurs with troublesome sleep disturbances [25] and unsettling lightheadedness, strongly suggesting the emergence of autonomic dysfunction [4, 14]. Unfortunately, these symptoms are commonly misinterpreted in isolation, often attributed solely to anxiety or behavioral issues [1, 9]. Many females begin puberty in this age range, which may exacerbate symptoms.

**During adolescence (12–18 years)**, studies (*n* = 10) show that the symptom profile crescendos, becoming truly multisystemic and relentlessly chronic [1, 4, 7–10, 12, 14, 16, 25]. Most females would be sexually mature during this timeframe while males likely mature toward the end of this time. Patients report diffuse musculoskeletal pain [1, 7, 10–12, 16], frequent subluxations or dislocations [1, 10, 12], and chronic headaches [10] (especially migraines or pressure-like pain exacerbated by posture or stress). Dysautonomia symptoms often solidify between 14 and 17 years of age [4, 14]. The immense psychosocial toll becomes profoundly evident [1]: adolescents often face debilitating school avoidance [10], pervasive depressive symptoms, and the deep invalidation that stems from their elusive diagnosis [1, 8]. While anxiety is undeniably prevalent (80% of a pediatric hEDS cohort had an anxiety diagnosis) [25], it is frequently secondary to the relentless burden of chronic pain, the demoralizing experience of being gaslighted, and severe functional limitations [1, 10, 25]. Crucially, psychological symptoms may also signal intrinsic neurodivergent traits, increasingly observed in this population (16–23% ADHD, 6% ASD in pediatric hEDS/HSD cohorts) [23].

### Impact on health-related quality of life (HRQoL) in pediatric hEDS/pgHSD

Beyond the mere presence and evolution of symptoms, this review highlights the profound and pervasive impact of hEDS/pgHSD on the health-related quality of life (HRQoL) of affected children and adolescents. Numerous recurring factors emerged as significant contributors to HRQoL deficits.

Chronic and recurrent musculoskeletal pain emerged as a primary factor, significantly diminishing daily activities, school attendance, and overall QoL [7, 10, 11, 18, 21, 31], often serving as a strong predictor of diminished HRQoL and functional disability [10]. Similarly, disabling fatigue was identified as a major predictor of reduced quality of life across various domains, including physical, emotional, and school functioning [8, 10], consistently correlating with more functional impairment and worse HRQoL [8, 31]. The presence of dysautonomia, cardiovascular, and autonomic symptoms such as orthostatic intolerance, POTS, dizziness, palpitations, chest pain, gastrointestinal issues significantly impact overall QoL [4, 12–14], directly contributing to reduced quality of life and functional impairment [4]. Furthermore, high rates of psychiatric and mental health conditions, including anxiety, depression, and generalized psychological distress (80% anxiety, 43% depression, 86% overall psychiatric diagnoses), were strongly associated with lower HRQoL [25], consistently demonstrating a negative impact [25]. Disrupted or non-restorative sleep likewise significantly impaired HRQoL [9, 12, 25] with sleep disturbances and poor quality of sleep negatively correlating with the degree of symptom burden and functional disability [12, 25].

The frequent experience of functional disability and impairment, manifesting as limitations in motor development, coordination, and physical activities [9, 21], profoundly impacted physical, emotional, and school domains [9, 10, 13, 31], and could even predict worsening quality of life over time [8, 18]. Chronic gastrointestinal issues like constipation, diarrhea, and dysphagia were frequently reported and significantly impacted children’s QoL [9, 12, 13, 17]. Finally, urinary tract dysfunction, including symptoms of urgency, enuresis, and frequency, were common and directly linked to impaired HRQoL [13, 23, 24], notably contributing to quality-of-life deficits [13, 23]. A broader concept, illness uncertainty, particularly due to diagnostic delays and the elusive nature of the condition, was also consistently associated with psychological distress and reduced quality of life [1]. The complex interplay of multiple comorbid symptoms and frequent misdiagnosis further contribute to the negative impact on patient experience and HRQoL [8, 9].

## Discussion

This Scoping review synthesizes the existing literature to identify eight recurring symptom clusters associated with hEDS, pgHSD and JHS. Beyond cataloguing individual symptoms, this review highlights the age-related evolution of multisystem manifestations across childhood and adolescence. The consistency of symptom clustering across musculoskeletal, autonomic, gastrointestinal, neurological, immune, and neurodevelopmental domains reinforces the understanding of symptomatic hypermobility spectrum disorders as systemic conditions with distinct pediatric-onset trajectories, rather than isolated musculoskeletal variants [1–4, 9–14]. Fragmented recognition of these patterns likely contributes to well-documented diagnostic delays and delayed access to appropriate care [3, 35].

### Multisystem symptom clustering as a pediatric diagnostic signal

While generalized joint hypermobility is a recognized feature of hypermobility-related disorders, this review underscores that multisystem symptom clustering represents a more informative clinical signal in pediatric populations. Clustering reflects recurring patterns across the literature rather than weighting by study focus. Across studies, symptom onset often occurred early in life, with reported mean onset around 6 years and some manifestations, such as lower urinary tract dysfunction, emerging in preschool-aged children [9, 24]. Children presenting with combinations of chronic musculoskeletal pain, autonomic symptoms, gastrointestinal complaints, fatigue, and neurodevelopmental traits, whether or not they meet full diagnostic criteria, frequently shared a similar clinical trajectory. These findings support the use of symptom constellations, rather than single features, to prompt consideration of pgHSD, particularly in the absence of definitive biomarkers [1–3, 10, 12]. Family history for hEDS/HSD or parents with suspected or diagnosed hEDS/HSD should increase suspicion when these symptom constellations are present.

Across age groups, features that repeatedly preceded diagnosis included pain disproportionate to objective findings, fatigue unrelieved by rest, recurrent joint instability despite normal imaging, and multisystem symptoms lacking a unifying diagnosis [7, 10, 12, 31, 36]. Recognition of these patterns may help clinicians identify children who warrant further connective tissue evaluation earlier in the diagnostic process.

### Diagnostic delay and functional consequences

Diagnostic delays of three to seven years were commonly reported across cohorts [1, 8–10]. During this period, children often experienced progressive physical deconditioning, functional limitations, school disruption, and increased psychological burden [8, 15, 21, 25]. Psychiatric diagnoses, including anxiety and depression, were frequently reported, particularly in adolescents, and often coexisted with chronic pain, fatigue, and autonomic dysfunction [25]. The literature also highlighted a tendency for physical symptoms to be misattributed to psychosomatic or behavioral causes, particularly in female patients, reflecting broader diagnostic challenges in pediatric chronic conditions [5, 14, 23, 25, 26, 37]. These findings emphasize the need for improved interdisciplinary awareness and structured approaches to early recognition.

### Comorbidities and overarching clinical themes

Neurodevelopmental traits that may include ADHD and ASD appear in at least one study to be intertwined with physical symptom burden [23]. The study by Kindgren et al. [23] found that patients diagnosed with ADHD or ASD had worse hEDS/pgHSD symptoms. Similarly, immune-mediated and mast cell activation–like symptoms, though not a focus in these studies, are thought to contribute to overall symptom burden and reduced quality of life [1, 10, 12]. Several cross-cutting themes were evident across age groups. Pain and proprioceptive dysfunction were often early but under-recognized features, while fatigue emerged as a major contributor to functional impairment during school-age years [7, 8]. As children entered adolescence, symptoms frequently progressed to include dysautonomia, gastrointestinal dysmotility, and psychosocial withdrawal [4, 10, 14]. Family history can increase clinical suspicion and may be useful in future screening frameworks as it supports the heritable nature of these conditions, despite lack of a single genetic marker for pgHSD/hEDS. Although the Beighton score remains a useful assessment tool, findings from this review reinforce that it is insufficient as a standalone measure in pediatric populations and should be interpreted within a broader, symptom cluster-based clinical framework [9, 11].

### Implications for screening and clinical practice

The true incidence and prevalence of pediatric hypermobility disorders remain uncertain due to evolving diagnostic criteria and under-recognition. Whereas isolated joint hypermobility is common and may often benign, our framework is intended to guide targeted clinical suspicion when multisystem features co-occur, rather than broad screening. The reproducibility of symptom clusters and their age-related evolution provides a foundation for the development of pediatric-focused screening approaches, analogous to adult hypermobility questionnaires. Such tools could support earlier triage and referral, particularly in non-specialist settings where delay in diagnoses occur. Our findings support earlier recognition emphasizing multisystem symptom clustering over isolated symptoms, highlighting “red flag” combinations (e.g., pain + fatigue + GI + autonomic symptoms), and supporting early multidisciplinary evaluation and referral. Across studies, best practices emphasized early recognition based on symptom patterns, multidisciplinary evaluation when indicated, and individualized physical therapy addressing joint instability and proprioceptive deficits [4, 8, 13]. Education for patients and families regarding the systemic nature of hypermobility-related disorders was also consistently highlighted as an important component of care.

### Limitations

This review has several limitations. Most included studies were observational and heterogeneous in design, with variable diagnostic terminology reflecting changes in classification over time. Formal diagnostic criteria were inconsistently applied, particularly in retrospective studies, and outcome measures varied widely across cohorts due to inherent limitations in the diagnostic criteria themselves. Many studies were conducted in specialty clinic populations, which may overrepresent more severely affected patients. Additionally, the predominance of female and White participants limits the generalizability of findings and highlights the need for more diverse pediatric cohorts.

Study selection and data charting were performed by a single reviewer, which may introduce selection bias despite the use of predefined criteria and standardized extraction methods. Finally, as a Scoping review, this work did not assess methodological quality or risk of bias, nor was it intended to evaluate treatment efficacy. Longitudinal, population-based studies using standardized pediatric frameworks are needed to validate symptom trajectories and inform age-appropriate interventions.

## Conclusion

This review provides a comprehensive mapping of the literature that is crucial given the pervasive diagnostic delays of hEDS/pgHSD stemming from the misinterpretation of seemingly disparate symptoms as isolated or benign complaints. By providing a clear framework of these evolving ‘red flags’ and their clinical pearls, these results can support early recognition of hEDS/ pgHSD, which is imperative for facilitating timely care to reduce the debilitating pain-fatigue-activity avoidance-deconditioning cycle. Early recognition could also further mitigate long-term morbidity, and ultimately preserve the developmental potential and HRQol of affected children. Future work should focus on validating age-specific screening tools, evaluating their impact on diagnostic timelines, and longitudinal measures linking pediatric symptom clusters to adult outcomes to better risk stratify patients.

## Supplementary Information

Below is the link to the electronic supplementary material.

Supplementary Material 1

Supplementary Material 2

Supplementary Material 3

## Data Availability

The datasets generated or analyzed during this study are included in this published article and its supplementary files.

## Abbreviations

hEDS: Hypermobile Ehlers-Danlos Syndrome (hEDS)
HSD: Hypermobility Spectrum Disorder (HSD)
pgHSD: Pediatric Generalized Hypermobility Spectrum Disorder (HSD) (pgHSD)
GJH: Generalized Joint Hypermobility
HCTD: Heritable Connective Tissue Disorder
ADHD: Attention-Deficit/Hyperactivity Disorder
ASD: Autism Spectrum Disorder
GI: Gastrointestinal
HRQoL: Health-Related Quality of Life
IU: Illness Uncertainty
PT: Physical Therapy
HMS: Hypermobility Syndrome
EDS-HT: Ehlers-Danlos Syndrome, Hypermobility Type
LUTD: Lower Urinary Tract Dysfunction
POTS: Postural Orthostatic Tachycardia Syndrome
AIS: Adolescent Idiopathic Scoliosis
QoL: Quality of Life
