# Supplementary material for: Age-related symptom clustering in pediatric hypermobility spectrum disorders: a scoping review
Source: Orphanet J Rare Dis. 2026 May 30;21:264. doi: 10.1186/s13023-026-04424-0 (PMC13435761; doi:10.1186/s13023-026-04424-0)
Supplement: Supplementary file 1 [file 13023_2026_4424_MOESM1_ESM.docx]

### **Appendix A:** Detailed Search Strategy for PubMed

This appendix provides the full search strategy used in PubMed to identify relevant studies for this scoping review.

**Database:** PubMed

**Search Date:** April 5^th^, 2025 up to May 8th, 2025

**Search Strategy:**

("Ehlers-Danlos Syndrome"[MeSH Terms] OR "hypermobile EDS"[tiab] OR "hypermobility spectrum disorder"[tiab] OR "joint hypermobility syndrome"[tiab]) AND

("pediatric"[tiab] OR "children"[tiab] OR "adolescents"[tiab]) AND

("symptoms"[tiab] OR "early signs"[tiab] OR "clinical presentation"[tiab] OR "diagnosis"[tiab] OR "screening"[tiab])

*Note: "[MeSH Terms]" indicates the use of Medical Subject Headings. "[tiab]" indicates a search in the title and abstract fields.* *Filters applied: English language, Human studies*
