# Supplementary material for: Age-related symptom clustering in pediatric hypermobility spectrum disorders: a scoping review
Source: Orphanet J Rare Dis. 2026 May 30;21:264. doi: 10.1186/s13023-026-04424-0 (PMC13435761; doi:10.1186/s13023-026-04424-0)
Supplement: Supplementary file 2 [file 13023_2026_4424_MOESM2_ESM.docx]

### **Supplementary File 3: Data Charting Form**

| **Data Field Category** | **Specific Data Points to Extract** | **Notes / Instructions for Extractor** |
| --- | --- | --- |
| **Study Identification** | Article ID (internal tracking number) | Assign sequentially |
|  | Authors | First author, et al. |
|  | Publication Year |  |
|  | Journal / Source |  |
|  | Study Design | e.g., Cross-sectional, Longitudinal, Case Series, Review, RCT, Cohort, Case-Control |
|  | Country of Study |  |
| **Population** | Sample Size (N) | Total number of participants |
|  | Age Range / Mean Age | Specify units (years, months) |
|  | Gender Distribution (% Female) |  |
|  | Diagnostic Criteria Used (for hEDS/HSD/JHS) | e.g., 2017 EDS criteria, Brighton, Beighton, previous historical definitions, etc. |
|  | Relevant Comorbidities | e.g., autism, ADHD, dysautonomia, GI issues — document prevalence if reported |
| **Symptom Patterns** | Early Clinical Clues (e.g., infant/toddler) | e.g., “floppy baby,” delayed motor milestones, unexplained irritability, excessive falls, frequent sprains/subluxations |
|  | School-Age Manifestations (6–12 yrs) | e.g., chronic pain (location, type), fatigue (severity, impact), proprioceptive dysfunction, increasing joint instability, early signs of dysautonomia, neurodevelopmental challenges |
|  | Adolescent Manifestations (13–18 yrs) | e.g., debilitating pain, severe fatigue, widespread dysautonomia, intractable GI issues, psychological sequelae, social withdrawal |
|  | Specific Symptom Categories Identified | e.g., Musculoskeletal, Autonomic, Gastrointestinal, Neurodevelopmental, Skin/Tissue, Sleep, ENT/Craniofacial, Psychological/Emotional |
| **"Red Flags"** | Specific signs or clusters suggesting pgHSD/hEDS | As per Table 2 in manuscript |
| **Diagnostic Pathway** | Reported age at symptom onset |  |
|  | Reported age at diagnosis |  |
|  | Diagnostic delay (years) | Calculate if possible |
|  | Misdiagnoses or misattributions | Commonly reported |
| **Outcomes** | HRQoL Measures / Findings |  |
|  | Functional Impairment | e.g., school attendance, physical activity limitations |
| **Management** | Recommended Practices / Strategies | e.g., individualized PT, multidisciplinary care, family education, psychological support |
| **Other Notes** | Additional insights, unique observations, or limitations | Relevant to the scoping review objectives |
