# Supplementary material for: Age-related symptom clustering in pediatric hypermobility spectrum disorders: a scoping review
Source: Orphanet J Rare Dis. 2026 May 30;21:264. doi: 10.1186/s13023-026-04424-0 (PMC13435761; doi:10.1186/s13023-026-04424-0)
Supplement: Supplementary file 3 [file 13023_2026_4424_MOESM3_ESM.docx]

**Supplementary File 2: Detailed Embase Search Strategy**

An experienced health librarian developed and ran a search in the Embase database (1974 – 2025 May 08) using the Ovid interface. The search was restricted to English language articles and an effort was made to only include pediatric or child-related articles. Search terms included Emtree terms as well as keywords, including terms for Ehlers-Danlos syndrome, hEDS, child, adolescent, juvenile, pediatric, symptoms, clinical feature, clinical presentation, clinical manifestation or diagnosis and their synonyms. A total of 119 records were found with the search.

Search strategy developed by **Tara Brigham, MLIS, AHIP-D**, Assistant Professor, Medical Education, Mayo Clinic Libraries - Florida.

**Results = 119 citations**

**1. Prevalence of Pain and Effect of Joint Strength on Perceived Physical Function in Pediatric Patients With Ehlers-Danlos Syndrome**

Garcia, N., et al. (2025). "Prevalence of Pain and Effect of Joint Strength on Perceived Physical Function in Pediatric Patients With Ehlers-Danlos Syndrome." Archives of Rehabilitation Research and Clinical Translation 7(1): 100416.

Objective: To identify how pediatric patients with hypermobile-type Ehlers-Danlos syndrome (hEDS) present to physical therapy (PT) and better understand their musculoskeletal dysfunction. <ovid:br/>Design(s): Patients were included in the study if they had a diagnosis of hEDS and at least 1 completed PT evaluation after diagnosis. A chart review was conducted to assess joint strength and pain characteristics at the initial PT assessment. Physical Function Mobility (PF-Mobility; 4 domains with 1-5 scale with higher score representative of better physical function) as part of the Patient-Reported Outcome Measurement Information System and pain intensity score (visual analog scale, 0-10 scale where a lower number indicates less pain) was completed by participants. <ovid:br/>Setting(s): Midwestern tertiary care center. <ovid:br/>Participant(s): Sixty-nine participants were included in the study with an average age of 16.4 years (SD 2.8) at the initial PT visit and 96% were female individuals. <ovid:br/>Intervention(s): Not applicable. <ovid:br/>Main Outcome Measure(s): Joint strength, pain intensity and location, and PF-Mobility. <ovid:br/>Result(s): The knee was the most reported location with pain (68%) followed by the shoulder (46%), hip (41%), and back (39%). Average pain intensity was 5.0 (SD 1.8), joint strength scores ranged from 8.2 (SD 0.9) to 9.9 (SD 0.4), and the PF-Mobility mean was 3.3 (SD 0.6). No correlation was noted between joint pain intensity and strength at the joint. <ovid:br/>Conclusion(s): Although the participants perceived poor mobility and significant pain, PT-assessed strength did not reveal significant muscle weakness. Additional work is needed to explore this discrepancy, including altered biomechanics, evaluation methods, and nonmusculoskeletal factors such as neuropathic and psychological components.<ovid:br/>Copyright &#xa9; 2024 The Authors

https://www.sciencedirect.com/science/journal/25901095

http://ovidsp.ovid.com/ovidweb.cgi?T=JS&PAGE=reference&D=emexb&NEWS=N&AN=2036564008

https://ovidsp.ovid.com/ovidweb.cgi?T=JS&CSC=Y&NEWS=N&PAGE=fulltext&D=emexb&DO=10.1016%2fj.arrct.2024.100416

http://sfxhosted.exlibrisgroup.com/mayo?sid=OVID:embase&id=pmid:&id=doi:10.1016%2Fj.arrct.2024.100416&issn=2590-1095&isbn=&volume=7&issue=1&spage=100416&pages=&date=2025&title=Archives+of+Rehabilitation+Research+and+Clinical+Translation&atitle=Prevalence+of+Pain+and+Effect+of+Joint+Strength+on+Perceived+Physical+Function+in+Pediatric+Patients+With+Ehlers-Danlos+Syndrome&aulast=Garcia&pid=%3Cauthor%3EGarcia+N.%3C%2Fauthor%3E&%3CAN%3E2036564008%3C%2FAN%3E

**2. Prevalence of psychiatric and sleep disorders and their impact on quality of life in children with hypermobile Ehlers-Danlos syndrome: an observational study**

Hertel, A. K., et al. (2025). "Prevalence of psychiatric and sleep disorders and their impact on quality of life in children with hypermobile Ehlers-Danlos syndrome: an observational study." Rheumatology International 45(4): 81.

Hypermobile Ehlers-Danlos Syndrome (hEDS) is the most common connective tissue disorder. However, few studies exist on psychiatric and sleep disorders in pediatric patients with hEDS. This study aims to describe psychiatric and sleep disorders and evaluate their impact on health-related quality of life (HRQoL) in pediatric patients with hEDS. As part of a longitudinal study, a convenience sample of 123 pediatric patients with hEDS, were recruited at a hEDS multidisciplinary clinic in sequential order over a seven-month period. Patient-reported outcomes were completed (Patient Reported Outcomes Measurement Information System Pediatric Profile Version 2 [PROMIS], Generalized Anxiety Disorder-7 [GAD-7], Adolescent Sleep Wake Scale [ASWS], and Pediatric Quality of Life Inventory Rheumatology Module [PEDS-QL Rheum]). The mean age was 15.8 years (SD = 2.7), the majority were female (92%) and Caucasian (92%). Most (86%) had at least one psychiatric diagnosis, with anxiety (80%) the most common, followed by depression (42%). Many (42%) also reported poor sleep. Correlations were seen between the GAD-7, PROMIS pain and HRQoL. Worse anxiety and depression were correlated with more sleep problems. Depression, GAD-7, and ASWS were also correlated with worse HRQoL. Performing regression analyses found anxiety and pain to be most predictive of HRQoL. Psychiatric and sleep disorders are prevalent in pediatric patients with hEDS and impact HRQoL negatively. Clinical focus on the anxiety and pain are important due to their impact on HRQoL.<ovid:br/>Copyright &#xa9; The Author(s) 2025.

https://www.springer.com/journal/296

http://ovidsp.ovid.com/ovidweb.cgi?T=JS&PAGE=reference&D=emexb&NEWS=N&AN=2033982283

https://ovidsp.ovid.com/ovidweb.cgi?T=JS&CSC=Y&NEWS=N&PAGE=fulltext&D=emexb&DO=10.1007%2fs00296-025-05836-0

http://sfxhosted.exlibrisgroup.com/mayo?sid=OVID:embase&id=pmid:40131551&id=doi:10.1007%2Fs00296-025-05836-0&issn=0172-8172&isbn=&volume=45&issue=4&spage=81&pages=&date=2025&title=Rheumatology+International&atitle=Prevalence+of+psychiatric+and+sleep+disorders+and+their+impact+on+quality+of+life+in+children+with+hypermobile+Ehlers-Danlos+syndrome%3A+an+observational+study&aulast=Hertel&pid=%3Cauthor%3EHertel+A.K.%3C%2Fauthor%3E&%3CAN%3E2033982283%3C%2FAN%3E

**3. A case of massive hematoma: reflections on hypermobile Ehlers-Danlos syndrome**

Liu, Q., et al. (2025). "A case of massive hematoma: reflections on hypermobile Ehlers-Danlos syndrome." Frontiers in Medicine 12: 1514349.

Ehlers-Danlos Syndrome (EDS) refers to a group of connective tissue disorders characterized by significant clinical and genetic variability, affecting multiple systems in the body. Classified as a rare disease, EDS includes 14 subtypes, all marked by joint hypermobility, skin extensibility, and tissue fragility. These subtypes present with a wide range of clinical manifestations and severities, including frequent joint dislocations, scoliosis, arterial dissections, and organ ruptures. Hypermobile EDS (hEDS) is the most common subtype, with newly established clinical diagnostic guidelines. In this case, a patient presented with minor hemoptysis over 8h, and a chest CT scan revealed a massive hematoma in the left lower lung. Due to the complexity and varied presentations of EDS, misdiagnosis is common. This report shares our experience with diagnosis and treatment in this case, highlighting the importance of increasing awareness for improved survival outcomes.<ovid:br/>Copyright &#xa9; 2025 Liu, Zeng, Xiong and Xu.

https://journal.frontiersin.org/journal/medicine

http://ovidsp.ovid.com/ovidweb.cgi?T=JS&PAGE=reference&D=emexb&NEWS=N&AN=2033379429

https://ovidsp.ovid.com/ovidweb.cgi?T=JS&CSC=Y&NEWS=N&PAGE=fulltext&D=emexb&DO=10.3389%2ffmed.2025.1514349

http://sfxhosted.exlibrisgroup.com/mayo?sid=OVID:embase&id=pmid:&id=doi:10.3389%2Ffmed.2025.1514349&issn=2296-858X&isbn=&volume=12&issue=&spage=1514349&pages=&date=2025&title=Frontiers+in+Medicine&atitle=A+case+of+massive+hematoma%3A+reflections+on+hypermobile+Ehlers-Danlos+syndrome&aulast=Liu&pid=%3Cauthor%3ELiu+Q.%3C%2Fauthor%3E&%3CAN%3E2033379429%3C%2FAN%3E

**4. Exploring signs of central sensitization in adolescents with hypermobility Spectrum disorder or hypermobile Ehlers-Danlos syndrome**

Schubert-Hjalmarsson, E., et al. (2025). "Exploring signs of central sensitization in adolescents with hypermobility Spectrum disorder or hypermobile Ehlers-Danlos syndrome." European Journal of Pain 29(1): e4754.

Background: Hypermobility Spectrum Disorder (HSD) and Hypermobile Ehlers-Danlos Syndrome (hEDS) are two overlapping heritable connective tissue disorders characterized by joint hypermobility, chronic pain, impaired body perception, and musculoskeletal symptoms. Central sensitization has been proposed as a plausible explanation for symptoms like widespread pain, fatigue, mood disorders, and sleep disturbances in patients with HSD/hEDS. <ovid:br/>Objective(s): The aim of this study was to investigate signs of central sensitization, including exercise-induced hypoalgesia (EIH), and fatigue severity in adolescents with HSD/hEDS. <ovid:br/>Method(s): In this prospective, experimental, case-control study, thirty-seven adolescents with HSD/hEDS and 47 healthy adolescents (all aged 13-17 years) were included. Pressure pain thresholds (PPTs) were measured at four muscle groups using a pressure algometer. EIH was evaluated by measuring PPTs on two muscle groups immediately after an exercise test on a bicycle ergometer. Participants also completed questionnaires on fatigue and cognitive/emotional factors. <ovid:br/>Result(s): The study demonstrated significantly lower PPTs in four different muscle groups in adolescents with HSD/hEDS compared to the healthy control group. Both groups achieved a significantly higher PPTs after exercise in the muscle involved in the activity. Adolescents with HSD/hEDS reported higher fatigue levels and more cognitive/emotional difficulties than the control group. <ovid:br/>Conclusion(s): Adolescents with HSD/hEDS showed generalized hyperalgesia measured through PPTs at different body sites. EIH was partly affected in adolescents with HSD/hEDS, presenting as unchanged pain sensitivity in the remote muscle. Pain should be considered as a phenomenon that is influenced by different biopsychosocial factors, including possible central sensitization, which increase its complexity. Significance statement: This study breaks new ground by showing signs of central sensitization, including diminished EIH, in adolescents with HSD or hEDS. Given that exercise is a key element in pain management, these findings offer valuable insights when developing treatment plans for adolescents with HSD or hEDS.<ovid:br/>Copyright &#xa9; 2024 The Author(s). European Journal of Pain published by John Wiley & Sons Ltd on behalf of European Pain Federation - EFIC .

http://onlinelibrary.wiley.com/journal/10.1002/(ISSN)1532-2149

http://ovidsp.ovid.com/ovidweb.cgi?T=JS&PAGE=reference&D=emed27&NEWS=N&AN=2032213948

https://ovidsp.ovid.com/ovidweb.cgi?T=JS&CSC=Y&NEWS=N&PAGE=fulltext&D=emed27&DO=10.1002%2fejp.4754

http://sfxhosted.exlibrisgroup.com/mayo?sid=OVID:embase&id=pmid:39529262&id=doi:10.1002%2Fejp.4754&issn=1090-3801&isbn=&volume=29&issue=1&spage=e4754&pages=&date=2025&title=European+Journal+of+Pain&atitle=Exploring+signs+of+central+sensitization+in+adolescents+with+hypermobility+Spectrum+disorder+or+hypermobile+Ehlers-Danlos+syndrome&aulast=Schubert-Hjalmarsson&pid=%3Cauthor%3ESchubert-Hjalmarsson+E.%3C%2Fauthor%3E&%3CAN%3E2032213948%3C%2FAN%3E

**5. Hypermobile type Ehlers-Danlos syndrome and generalized hypermobile spectrum disorder treatment preferences - a cross-sectional survey of patients**

Teran-Wodzinski, P. and A. Kumar (2025). "Hypermobile type Ehlers-Danlos syndrome and generalized hypermobile spectrum disorder treatment preferences - a cross-sectional survey of patients." Rheumatology International 45(1): 25.

We aimed to assess the typical experiences, desired outcomes, satisfaction with clinical and anticipated outcomes, and the importance of improvements for individuals with Hypermobile Ehlers-Danlos Syndrome (hEDS) and Generalized Hypermobility Spectrum Disorder (G-HSD). A cross-sectional survey was conducted among adults aged 18 and above with hEDS and G-HSD. The survey included the Patient-Centered Outcome Questionnaire and an adapted version addressing common concerns in these individuals. Descriptive statistics were used for analysis. The survey received 483 responses with an 82% completion rate. Most respondents were females (90%), aged 21-30 (30%), living in North America (76%), and diagnosed with hEDS (80%). Participants diagnosed with hEDS reported higher typical levels of pain compared to those diagnosed with G-HSD and higher expected levels of pain and interference with daily activities post-treatment (p < 0.05). The areas of most significant concern were pain, fatigue, interference with daily activities, and walking issues. Our findings revealed no differences in how individuals from both groups rated their treatment expectations, except for the usual pain level and the expected pain level and interference with daily activities post-treatment. Patients' perspectives are essential for developing appropriate treatment plans and improving outcomes for this patient population. Our results will hopefully inform the development of new interventions to impact outcomes that matter to individuals with hEDS and G-HSD.<ovid:br/>Copyright &#xa9; The Author(s), under exclusive licence to Springer-Verlag GmbH Germany, part of Springer Nature 2025.

https://www.springer.com/journal/296

http://ovidsp.ovid.com/ovidweb.cgi?T=JS&PAGE=reference&D=emexb&NEWS=N&AN=2033123200

https://ovidsp.ovid.com/ovidweb.cgi?T=JS&CSC=Y&NEWS=N&PAGE=fulltext&D=emexb&DO=10.1007%2fs00296-024-05782-3

http://sfxhosted.exlibrisgroup.com/mayo?sid=OVID:embase&id=pmid:39804414&id=doi:10.1007%2Fs00296-024-05782-3&issn=0172-8172&isbn=&volume=45&issue=1&spage=25&pages=&date=2025&title=Rheumatology+International&atitle=Hypermobile+type+Ehlers-Danlos+syndrome+and+generalized+hypermobile+spectrum+disorder+treatment+preferences+-+a+cross-sectional+survey+of+patients&aulast=Teran-Wodzinski&pid=%3Cauthor%3ETeran-Wodzinski+P.%3C%2Fauthor%3E&%3CAN%3E2033123200%3C%2FAN%3E

**6. The Spider; A Multisystemic Symptom Impact Tool for People with Hypermobility-Related Disorders. Initial Validation in Adolescents**

Ewer, E., et al. (2024). "The Spider; A Multisystemic Symptom Impact Tool for People with Hypermobility-Related Disorders. Initial Validation in Adolescents." Journal of Pediatrics: Clinical Practice 12: 200098.

Objectives: The multisystemic comorbid symptoms/conditions of hypermobility spectrum disorders (HSD) and hypermobile Ehlers-Danlos syndrome (hEDS) often significantly affect daily life. Many of these symptoms are under-recognized during diagnosis and treatment; therefore, a comprehensive questionnaire was developed to evaluate their presence and impact. The Spider's 8 domains assess neuromusculoskeletal, pain, fatigue, cardiac dysautonomia, urogenital, gastrointestinal, anxiety, and depression symptoms. This study aims to evaluate the construct validity of the Spider in adolescents. Study design: This cross-sectional study recruited international participants through 3 patient charities and a hypermobility unit. Adolescents aged 13-18 years, with and without HSD/hEDS, were included. Validated and frequently used comparator questionnaires were used to establish convergent validity. Participants answered each Spider domain and the respective comparator via 4 online surveys. Convergent validity was assessed by comparing Spider domain and comparator scores through correlational analysis. Known-group validity was assessed by comparing Spider domain scores of hypermobile and control groups using Mann-Whitney U analysis. <ovid:br/>Result(s): In total, 1154 adolescents participated, 1036 with HSD/hEDS and 118 controls. Six domains (pain, fatigue, depression, cardiac dysautonomia, gastrointestinal, and neuromusculoskeletal) demonstrated strong correlations (r = -0.7 to 0.8) with the respective comparator. The urogenital and anxiety domains showed moderate correlations (r = 0.6). All correlations reached statistical significance (P < .001). Known-group validity was demonstrated in 7 domains (P < .001). The urogenital domain did not show a significant difference between the groups (P = .094). <ovid:br/>Conclusion(s): The Spider domains demonstrate acceptable convergent validity. The Spider questionnaire can accurately, rapidly, and concisely measure the effect of common multisystemic comorbid symptoms/conditions associated with HSD/hEDS to help direct care in adolescents.<ovid:br/>Copyright &#xa9; 2024 The Author(s)

https://www.sciencedirect.com/journal/the-journal-of-pediatrics-clinical-practice/about/editorial-board

http://ovidsp.ovid.com/ovidweb.cgi?T=JS&PAGE=reference&D=emed27&NEWS=N&AN=2032488132

https://ovidsp.ovid.com/ovidweb.cgi?T=JS&CSC=Y&NEWS=N&PAGE=fulltext&D=emed27&DO=10.1016%2fj.jpedcp.2024.200098

http://sfxhosted.exlibrisgroup.com/mayo?sid=OVID:embase&id=pmid:&id=doi:10.1016%2Fj.jpedcp.2024.200098&issn=2950-5410&isbn=&volume=12&issue=&spage=200098&pages=&date=2024&title=Journal+of+Pediatrics%3A+Clinical+Practice&atitle=The+Spider%3B+A+Multisystemic+Symptom+Impact+Tool+for+People+with+Hypermobility-Related+Disorders.+Initial+Validation+in+Adolescents&aulast=Ewer&pid=%3Cauthor%3EEwer+E.%3C%2Fauthor%3E&%3CAN%3E2032488132%3C%2FAN%3E

**7. Functional neurological signs in hypermobile Ehlers-Danlos syndrome and hypermobile spectrum disorders with suspected neuropathic pain**

Fernandez, A., et al. (2024). "Functional neurological signs in hypermobile Ehlers-Danlos syndrome and hypermobile spectrum disorders with suspected neuropathic pain." Brain and Behavior 14(2): e3441.

Background: The hypermobile Ehlers-Danlos syndrome (hEDS) and hypermobility spectrum disorders (HSD) are connective tissue disorders characterized by generalized joint hypermobility, associated with chronic pain and several symptoms, such as fatigue, dysautonomia, as well as psychiatric co-morbidities. Clinical observations of unusual manifestations during systematic sensory testing raised the question of a possible co-existence with a functional neurological disorder (FND). Hence, this study aimed to assess the presence of positive functional neurological signs (FNS) in a cohort of patients with hEDS/HSD. <ovid:br/>Method(s): The clinical data of hEDS/HSD patients (N = 24) were retrospectively analyzed and compared to a prospectively recruited age-/sex-matched healthy control group (N = 22). Four motor- and three sensory-positive FNS were assessed. <ovid:br/>Result(s): Twenty-two patients (92%) presented at least one motor or sensory FNS. Five patients (21%) presented only a single FNS, 14 presented between 2 and 4 FNS (58%), and 3 patients presented 5 or more FNS (12%). None of the healthy controls presented motor FNS, and only two presented a sensory FNS. <ovid:br/>Conclusion(s): The presence of FNS in hEDS/HSD deserves better clinical detection and formal diagnosis of FND to offer more adequate care in co-morbid situations. In fact, FND can severely interfere with rehabilitation efforts in hEDS/HSD, and FND-targeted physical therapy should perhaps be combined with EDS/HSD-specific approaches.<ovid:br/>Copyright &#xa9; 2024 The Authors. Brain and Behavior published by Wiley Periodicals LLC.

http://onlinelibrary.wiley.com/journal/10.1002/(ISSN)2157-9032

http://ovidsp.ovid.com/ovidweb.cgi?T=JS&PAGE=reference&D=emed26&NEWS=N&AN=2028769281

https://ovidsp.ovid.com/ovidweb.cgi?T=JS&CSC=Y&NEWS=N&PAGE=fulltext&D=emed26&DO=10.1002%2fbrb3.3441

http://sfxhosted.exlibrisgroup.com/mayo?sid=OVID:embase&id=pmid:38409931&id=doi:10.1002%2Fbrb3.3441&issn=2162-3279&isbn=&volume=14&issue=2&spage=e3441&pages=&date=2024&title=Brain+and+Behavior&atitle=Functional+neurological+signs+in+hypermobile+Ehlers-Danlos+syndrome+and+hypermobile+spectrum+disorders+with+suspected+neuropathic+pain&aulast=Fernandez&pid=%3Cauthor%3EFernandez+A.%3C%2Fauthor%3E&%3CAN%3E2028769281%3C%2FAN%3E

**8. Editorial: Research advances in understanding the etiology, epidemiology, pathophysiology, clinical features, and management of the Ehlers Danlos syndrome disorders**

Francomano, C. A., et al. (2024). "Editorial: Research advances in understanding the etiology, epidemiology, pathophysiology, clinical features, and management of the Ehlers Danlos syndrome disorders." Frontiers in Medicine 11: 1364308.

https://journal.frontiersin.org/journal/medicine

http://ovidsp.ovid.com/ovidweb.cgi?T=JS&PAGE=reference&D=emed27&NEWS=N&AN=2030845687

https://ovidsp.ovid.com/ovidweb.cgi?T=JS&CSC=Y&NEWS=N&PAGE=fulltext&D=emed27&DO=10.3389%2ffmed.2024.1364308

http://sfxhosted.exlibrisgroup.com/mayo?sid=OVID:embase&id=pmid:&id=doi:10.3389%2Ffmed.2024.1364308&issn=2296-858X&isbn=&volume=11&issue=&spage=1364308&pages=&date=2024&title=Frontiers+in+Medicine&atitle=Editorial%3A+Research+advances+in+understanding+the+etiology%2C+epidemiology%2C+pathophysiology%2C+clinical+features%2C+and+management+of+the+Ehlers+Danlos+syndrome+disorders&aulast=Francomano&pid=%3Cauthor%3EFrancomano+C.A.%3C%2Fauthor%3E&%3CAN%3E2030845687%3C%2FAN%3E

**9. Craniocervical instability in patients with Ehlers-Danlos syndromes: outcomes analysis following occipito-cervical fusion**

Henderson, F. C., et al. (2024). "Craniocervical instability in patients with Ehlers-Danlos syndromes: outcomes analysis following occipito-cervical fusion." Neurosurgical Review 47(1): 27.

Craniocervical instability (CCI) is increasingly recognized in hereditary disorders of connective tissue and in some patients following suboccipital decompression for Chiari malformation (CMI) or low-lying cerebellar tonsils (LLCT). CCI is characterized by severe headache and neck pain, cervical medullary syndrome, lower cranial nerve deficits, myelopathy, and radiological metrics, for which occipital cervical fusion (OCF) has been advocated. We conducted a retrospective analysis of patients with CCI and Ehlers-Danlos syndrome (EDS) to determine whether the surgical outcomes supported the criteria by which patients were selected for OCF. Fifty-three consecutive subjects diagnosed with EDS, who presented with severe head and neck pain, lower cranial nerve deficits, cervical medullary syndrome, myelopathy, and radiologic findings of CCI, underwent open reduction, stabilization, and OCF. Thirty-two of these patients underwent suboccipital decompression for obstruction of cerebral spinal fluid flow. Questionnaire data and clinical findings were abstracted by a research nurse. Follow-up questionnaires were administered at 5-28 months (mean 15.1). The study group demonstrated significant improvement in headache and neck pain (p < 0.001), decreased use of pain medication (p < 0.0001), and improved Karnofsky Performance Status score (p < 0.001). Statistically significant improvement was also demonstrated for nausea, syncope (p < 0.001), speech difficulties, concentration, vertigo, dizziness, numbness, arm weakness, and fatigue (p = 0.001). The mental fatigue score and orthostatic grading score were improved (p < 0.01). There was no difference in pain improvement between patients with CMI/LLCT and those without. This outcomes analysis of patients with disabling CCI in the setting of EDS demonstrated significant benefits of OCF. The results support the reasonableness of the selection criteria for OCF. We advocate for a multi-center, prospective clinical trial of OCF in this population.<ovid:br/>Copyright &#xa9; 2023, The Author(s).

https://www.springer.com/journal/10143

http://ovidsp.ovid.com/ovidweb.cgi?T=JS&PAGE=reference&D=emed26&NEWS=N&AN=2027693021

https://ovidsp.ovid.com/ovidweb.cgi?T=JS&CSC=Y&NEWS=N&PAGE=fulltext&D=emed26&DO=10.1007%2fs10143-023-02249-0

http://sfxhosted.exlibrisgroup.com/mayo?sid=OVID:embase&id=pmid:38163828&id=doi:10.1007%2Fs10143-023-02249-0&issn=0344-5607&isbn=&volume=47&issue=1&spage=27&pages=&date=2024&title=Neurosurgical+Review&atitle=Craniocervical+instability+in+patients+with+Ehlers-Danlos+syndromes%3A+outcomes+analysis+following+occipito-cervical+fusion&aulast=Henderson&pid=%3Cauthor%3EHenderson+F.C.%3C%2Fauthor%3E&%3CAN%3E2027693021%3C%2FAN%3E

**10. Cardiovascular Symptoms, Dysautonomia, and Quality of Life in Adult and Pediatric Patients with Hypermobile Ehlers-Danlos Syndrome: A Brief Review**

Hertel, A., et al. (2024). "Cardiovascular Symptoms, Dysautonomia, and Quality of Life in Adult and Pediatric Patients with Hypermobile Ehlers-Danlos Syndrome: A Brief Review." Current Cardiology Reviews 20(1): e240124226070.

Background: Hypermobile Ehlers-Danlos Syndrome (hEDS) is a connective tissue disorder characterized by joint hypermobility and other systemic manifestations, such as cardiovascular symptoms, musculoskeletal pain, and joint instability. Cardiovascular symptoms, such as lightheadedness and palpitations, and types of dysautonomia, including postural orthostatic tachycardia syndrome (POTS), are frequently reported in adults with hEDS and have been shown to negatively impact quality of life (QoL). <ovid:br/>Objective(s): This brief review will be an overview of co-occurring symptoms in POTS and hEDS to inform potential cardiovascular screening procedures. <ovid:br/>Result(s): While many patients with hEDS report cardiovascular symptoms, few have structural abnormalities, suggesting that dysautonomia is likely responsible for these symptoms. One validated screening measure for dysautonomia symptom burden is the Composite Autonomic Symptom Scale (COMPASS-31). Studies have found that adults with POTS, hEDS, and both POTS and hEDS have higher COMPASS-31 scores than the general population, suggesting a high symptom burden due to dysautonomia, which leads to impaired QoL. <ovid:br/>Conclusion(s): While studies have examined cardiovascular symptoms and the impact of dysautonomia in adults with and without hEDS, there is scant literature on dysautonomia in pediatric patients with hEDS. Therefore, more studies on cardiovascular symptoms and dysautonomia, as they relate to the quality of life in pediatric patients with hEDS, are needed. This brief review summarizes the current literature on dysautonomia and cardiovascular symptoms in pediatric and adult populations with hEDS.<ovid:br/>Copyright &#xa9; 2024 Bentham Science Publishers.

https://www.eurekaselect.com/article/137768

http://ovidsp.ovid.com/ovidweb.cgi?T=JS&PAGE=reference&D=emed26&NEWS=N&AN=2028394601

https://ovidsp.ovid.com/ovidweb.cgi?T=JS&CSC=Y&NEWS=N&PAGE=fulltext&D=emed26&DO=10.2174%2f011573403X271096231203164216

http://sfxhosted.exlibrisgroup.com/mayo?sid=OVID:embase&id=pmid:38275067&id=doi:10.2174%2F011573403X271096231203164216&issn=1573-403X&isbn=&volume=20&issue=1&spage=e240124226070&pages=&date=2024&title=Current+Cardiology+Reviews&atitle=Cardiovascular+Symptoms%2C+Dysautonomia%2C+and+Quality+of+Life+in+Adult+and+Pediatric+Patients+with+Hypermobile+Ehlers-Danlos+Syndrome%3A+A+Brief+Review&aulast=Hertel&pid=%3Cauthor%3EHertel+A.%3C%2Fauthor%3E&%3CAN%3E2028394601%3C%2FAN%3E

**11. Cardiovascular, autonomic symptoms and quality of life in children with hypermobile Ehlers-Danlos syndrome**

Hertel, A. K., et al. (2024). "Cardiovascular, autonomic symptoms and quality of life in children with hypermobile Ehlers-Danlos syndrome." SAGE Open Medicine 12: 20503121241287073.

Objectives: Hypermobile Ehlers-Danlos syndrome is a connective tissue disorder characterized by joint hypermobility and other systemic manifestations. Cardiovascular, autonomic symptoms and dysautonomia are frequently reported in adults with hypermobile Ehlers-Danlos syndrome and have been shown to have a negative impact on quality of life. However, there is scant literature on autonomic symptoms in pediatric patients with hypermobile Ehlers-Danlos syndrome. This study aims to characterize cardiovascular symptoms and diagnoses in pediatric patients with hypermobile Ehlers-Danlos syndrome and evaluate the impact of autonomic symptoms on quality of life. <ovid:br/>Method(s): As part of a longitudinal study, a consecutive sample of 70 patients with Ehlers-Danlos syndromes were recruited at routine clinical care visits. Medical history was reviewed, demographics were obtained, and patient-reported outcomes were completed by the patients. <ovid:br/>Result(s): The average age of 70 patients was 15.8 years, and the majority were females (89%) and Caucasian (89%). The most common cardiovascular diagnoses were orthostatic intolerance (59%), dysautonomia (47%), and postural orthostatic tachycardia syndrome (21%). Most patients had an echocardiogram (77%), that was normal (82%). No patients had mitral valve prolapse, and only one patient had mild aortic root dilation (2%). Patient-reported outcomes revealed decreased quality of life associated with autonomic symptoms. <ovid:br/>Conclusion(s): This study shows that most children with hypermobile Ehlers-Danlos syndrome have cardiovascular and autonomic symptoms, which have a negative impact on quality of life. Few patients with hypermobile Ehlers-Danlos syndrome have structural abnormalities on echocardiogram, which suggests that the cardiovascular symptoms experienced by patients are not due to structural cardiovascular disease and possibly reflective of autonomic pathology, though further studies will need to confirm this. This study confirms that cardiovascular and symptoms are prevalent and have a dramatic impact on quality of life in pediatric and young adult patients diagnosed with hypermobile Ehlers-Danlos syndrome.<ovid:br/>Copyright &#xa9; The Author(s) 2024.

https://journals.sagepub.com/home/SMO

http://ovidsp.ovid.com/ovidweb.cgi?T=JS&PAGE=reference&D=emed27&NEWS=N&AN=2031889767

https://ovidsp.ovid.com/ovidweb.cgi?T=JS&CSC=Y&NEWS=N&PAGE=fulltext&D=emed27&DO=10.1177%2f20503121241287073

http://sfxhosted.exlibrisgroup.com/mayo?sid=OVID:embase&id=pmid:&id=doi:10.1177%2F20503121241287073&issn=2050-3121&isbn=&volume=12&issue=&spage=&pages=&date=2024&title=SAGE+Open+Medicine&atitle=Cardiovascular%2C+autonomic+symptoms+and+quality+of+life+in+children+with+hypermobile+Ehlers-Danlos+syndrome&aulast=Hertel&pid=%3Cauthor%3EHertel+A.K.%3C%2Fauthor%3E&%3CAN%3E2031889767%3C%2FAN%3E

**12. Laryngological Complaint Prevalence in Hypermobile Ehlers-Danlos or Hypermobility Spectrum Disorders**

Menton, S. M., et al. (2024). "Laryngological Complaint Prevalence in Hypermobile Ehlers-Danlos or Hypermobility Spectrum Disorders." Laryngoscope 134(2): 773

EP - 778.

Objective: The aim was to study laryngological complaints in patients with hypermobile Ehlers-Danlos syndrome (hEDS) or hypermobility spectrum disorders (HSD). <ovid:br/>Method(s): A total of 363 patients met inclusion for the study by completing questions related to voice, upper airway, and swallowing between July 7, 2020 and July 13, 2022. Demographic data, voice-related questions, and hypermobility diagnosis were analyzed retrospectively. From those, 289 patients were diagnosed with hEDS or HSD with 74 that did not meet the diagnostic criteria for either diagnosis serving as controls. <ovid:br/>Result(s): There were no statistically significant differences between patients with hEDS and HSD regarding Voice Handicap Index (VHI-10) scores, voice, upper airway, or swallow complaints. However, more hEDS/HSD patients answered positively to the laryngeal dysfunction question versus controls (p = 0.031). 22.5% of hEDS/HSD patients (n = 65) reported hoarseness, of which 52.3% reported hoarseness >2 days/month. 33.9% (n = 98) with hEDS/HSD reported symptoms of dysphagia, and 27.0% (n = 78) reported laryngeal dysfunction symptoms. Controls demonstrated 20.3% prevalence of hoarseness, of which 46.7% reported hoarseness >2 days/month. 24.3% of controls had dysphagia and 14.9% laryngeal dysfunction symptoms. Of the 363 patients, VHI-10 scores >11 were more likely in patients reporting >2 days of hoarseness/month (p = 0.001) versus those with <2 days of hoarseness/month. There was an increased prevalence of voice, upper airway, and dysphagia symptoms in hEDS/HSD patients compared with previously reported prevalence data in the general population. <ovid:br/>Conclusion(s): A significant proportion of patients diagnosed with hypermobility due to hEDS or HSD were found to have voice, upper airway, and dysphagia symptoms. These rates are higher than those previously reported in the general population. <ovid:br/>Level of Evidence: 3 Laryngoscope, 134:773-778, 2024.<ovid:br/>Copyright &#xa9; 2023 The American Laryngological, Rhinological and Otological Society, Inc.

http://onlinelibrary.wiley.com/journal/10.1002/(ISSN)1531-4995

http://ovidsp.ovid.com/ovidweb.cgi?T=JS&PAGE=reference&D=emed26&NEWS=N&AN=2024958958

https://ovidsp.ovid.com/ovidweb.cgi?T=JS&CSC=Y&NEWS=N&PAGE=fulltext&D=emed26&DO=10.1002%2flary.30964

http://sfxhosted.exlibrisgroup.com/mayo?sid=OVID:embase&id=pmid:37597175&id=doi:10.1002%2Flary.30964&issn=0023-852X&isbn=&volume=134&issue=2&spage=773&pages=773-778&date=2024&title=Laryngoscope&atitle=Laryngological+Complaint+Prevalence+in+Hypermobile+Ehlers-Danlos+or+Hypermobility+Spectrum+Disorders&aulast=Menton&pid=%3Cauthor%3EMenton+S.M.%3C%2Fauthor%3E&%3CAN%3E2024958958%3C%2FAN%3E

**13. Early Diagnosis of Vascular Ehlers-Danlos Syndrome Through AI-Powered Facial Analysis: Results from the Montalcino Aortic Consortium**

Murdock, D. R., et al. (2024). "Early Diagnosis of Vascular Ehlers-Danlos Syndrome Through AI-Powered Facial Analysis: Results from the Montalcino Aortic Consortium." medRxiv.

Purpose: Vascular Ehlers-Danlos syndrome (vEDS), caused by COL3A1 pathogenic variants, is a rare heritable aortic and arterial disorder associated with early mortality, mainly due to spontaneous vascular dissections and ruptures. Improved methods for diagnosing vEDS are needed so that guideline-based management can be initiated to prevent deadly complications and differentiate vEDS from overlapping conditions like hypermobile EDS (hEDS). <ovid:br/>Method(s): We implemented an AI facial recognition model based on the PhenoScore framework using a support vector machine (SVM) trained on facial images of thirty individuals, aged 6-65 years, with vEDS from the Montalcino Aortic Consortium (MAC), control images from the Chicago Face Database (CFD), and publicly available images of individuals with hEDS. Cross-validation was used to train the SVM, and statistical measures to evaluate the model performance were calculated. Local Interpretable Model-agnostic Explanations (LIME) was used to generate facial heatmaps highlighting the features driving the model's predictions. <ovid:br/>Result(s): The AI classifier showed excellent performance with as few as thirteen vEDS training images and distinguished vEDS from both controls and individuals with hEDS with high accuracy, achieving an area under the receiver operating characteristic curve (AUC) >= 0.97. LIME highlighted facial regions already established to characterize the facial features of vEDS patients (e.g., prominent eyes). <ovid:br/>Conclusion(s): Our results demonstrate the potential of AI-based facial analysis for diagnosing vEDS. This method democratizes the early diagnosis of vEDS by reducing dependence on genetic testing, enabling optimal management and improved outcomes, particularly in resource-limited areas.<ovid:br/>Copyright The copyright holder for this preprint is the author/funder, who has granted medRxiv a license to display the preprint in perpetuity. All rights reserved. No reuse allowed without permission.

https://www.medrxiv.org/

http://ovidsp.ovid.com/ovidweb.cgi?T=JS&PAGE=reference&D=empp&NEWS=N&AN=2035580523

https://ovidsp.ovid.com/ovidweb.cgi?T=JS&CSC=Y&NEWS=N&PAGE=fulltext&D=empp&DO=10.1101%2f2024.10.20.24315773

http://sfxhosted.exlibrisgroup.com/mayo?sid=OVID:embase&id=pmid:&id=doi:10.1101%2F2024.10.20.24315773&issn=&isbn=&volume=&issue=&spage=&pages=&date=2024&title=medRxiv&atitle=Early+Diagnosis+of+Vascular+Ehlers-Danlos+Syndrome+Through+AI-Powered+Facial+Analysis%3A+Results+from+the+Montalcino+Aortic+Consortium&aulast=Murdock&pid=%3Cauthor%3EMurdock+D.R.%3C%2Fauthor%3E&%3CAN%3E2035580523%3C%2FAN%3E

**14. Joint Hypermobility Syndrome and Membrane Proteins: A Comprehensive Review**

Pliego-Arreaga, R., et al. (2024). "Joint Hypermobility Syndrome and Membrane Proteins: A Comprehensive Review." Biomolecules 14(4): 472.

Ehlers-Danlos syndromes (EDSs) constitute a heterogeneous group of connective tissue disorders characterized by joint hypermobility, skin hyperextensibility, and tissue fragility. Asymptomatic EDSs, joint hypermobility without associated syndromes, EDSs, and hypermobility spectrum disorders are the commonest phenotypes associated with joint hypermobility. Joint hypermobility syndrome (JHS) is a connective tissue disorder characterized by extreme flexibility of the joints, along with pain and other symptoms. JHS can be a sign of a more serious underlying genetic condition, such as EDS, which affects the cartilage, bone, fat, and blood. The exact cause of JHS could be related to genetic changes in the proteins that add flexibility and strength to the joints, ligaments, and tendons, such as collagen. Membrane proteins are a class of proteins embedded in the cell membrane and play a crucial role in cell signaling, transport, and adhesion. Dysregulated membrane proteins have been implicated in a variety of diseases, including cancer, cardiovascular disease, and neurological disorders; recent studies have suggested that membrane proteins may also play a role in the pathogenesis of JHS. This article presents an exploration of the causative factors contributing to musculoskeletal pain in individuals with hypermobility, based on research findings. It aims to provide an understanding of JHS and its association with membrane proteins, addressing the clinical manifestations, pathogenesis, diagnosis, and management of JHS.<ovid:br/>Copyright &#xa9; 2024 by the authors.

http://www.mdpi.com/journal/biomolecules

http://ovidsp.ovid.com/ovidweb.cgi?T=JS&PAGE=reference&D=emed26&NEWS=N&AN=2029591890

https://ovidsp.ovid.com/ovidweb.cgi?T=JS&CSC=Y&NEWS=N&PAGE=fulltext&D=emed26&DO=10.3390%2fbiom14040472

http://sfxhosted.exlibrisgroup.com/mayo?sid=OVID:embase&id=pmid:38672488&id=doi:10.3390%2Fbiom14040472&issn=2218-273X&isbn=&volume=14&issue=4&spage=472&pages=&date=2024&title=Biomolecules&atitle=Joint+Hypermobility+Syndrome+and+Membrane+Proteins%3A+A+Comprehensive+Review&aulast=Pliego-Arreaga&pid=%3Cauthor%3EPliego-Arreaga+R.%3C%2Fauthor%3E&%3CAN%3E2029591890%3C%2FAN%3E

**15. Looking back and beyond the 2017 diagnostic criteria for hypermobile Ehlers-Danlos syndrome: A retrospective cross-sectional study from an Italian reference center**

Ritelli, M., et al. (2024). "Looking back and beyond the 2017 diagnostic criteria for hypermobile Ehlers-Danlos syndrome: A retrospective cross-sectional study from an Italian reference center." American Journal of Medical Genetics, Part A 194(2): 174

EP - 194.

The most common conditions with symptomatic joint hypermobility are hypermobile Ehlers-Danlos syndrome (hEDS) and hypermobility spectrum disorders (HSD). Diagnosing these overlapping connective tissue disorders remains challenging due to the lack of established causes and reliable diagnostic tests. hEDS is diagnosed applying the 2017 diagnostic criteria, and patients with symptomatic joint hypermobility but not fulfilling these criteria are labeled as HSD, which is not officially recognized by all healthcare systems. The 2017 criteria were introduced to improve diagnostic specificity but have faced criticism for being too stringent and failing to adequately capture the multisystemic involvement of hEDS. Herein, we retrospectively evaluated 327 patients from 213 families with a prior diagnosis of hypermobility type EDS or joint hypermobility syndrome based on Villefranche and Brighton criteria, to assess the effectiveness of the 2017 criteria in distinguishing between hEDS and HSD and document the frequencies of extra-articular manifestations. Based on our findings, we propose that the 2017 criteria should be made less stringent to include a greater number of patients who are currently encompassed within the HSD category. This will lead to improved diagnostic accuracy and enhanced patient care by properly capturing the diverse range of symptoms and manifestations present within the hEDS/HSD spectrum.<ovid:br/>Copyright &#xa9; 2023 The Authors. American Journal of Medical Genetics Part A published by Wiley Periodicals LLC.

http://onlinelibrary.wiley.com/journal/10.1002/(ISSN)1552-4833

http://ovidsp.ovid.com/ovidweb.cgi?T=JS&PAGE=reference&D=emed26&NEWS=N&AN=2025793240

https://ovidsp.ovid.com/ovidweb.cgi?T=JS&CSC=Y&NEWS=N&PAGE=fulltext&D=emed26&DO=10.1002%2fajmg.a.63426

http://sfxhosted.exlibrisgroup.com/mayo?sid=OVID:embase&id=pmid:37774134&id=doi:10.1002%2Fajmg.a.63426&issn=1552-4825&isbn=&volume=194&issue=2&spage=174&pages=174-194&date=2024&title=American+Journal+of+Medical+Genetics%2C+Part+A&atitle=Looking+back+and+beyond+the+2017+diagnostic+criteria+for+hypermobile+Ehlers-Danlos+syndrome%3A+A+retrospective+cross-sectional+study+from+an+Italian+reference+center&aulast=Ritelli&pid=%3Cauthor%3ERitelli+M.%3C%2Fauthor%3E&%3CAN%3E2025793240%3C%2FAN%3E

**16. High prevalence of gastrointestinal disorders in a large cohort of patients with joint hypermobility**

Sood, V., et al. (2024). "High prevalence of gastrointestinal disorders in a large cohort of patients with joint hypermobility." Journal of Pediatric Gastroenterology and Nutrition 79(1): 42

EP - 47.

The gastrointestinal (GI) manifestations in children with hypermobile Ehlers-Danlos syndrome/joint hypermobility syndrome (hEDS/JHS) are not well described. We investigated the prevalence of GI disorders in children and young adults with hEDS/JHS through a single-center retrospective review. Demographic data, clinical history, symptoms, and diagnostic studies were reviewed. Of 435 patients with hEDS/JHS, 66% were females (age 5-28 years). We noted a high prevalence of constipation (61%), dysphagia (32%), dyspepsia and/or gastroparesis (25%), eosinophilic esophagitis (EoE) (21%), and celiac disease (4%) in our cohort. Upper endoscopy and gastric emptying scans had the highest yield to detect abnormalities. Motility studies were abnormal in 31% of the 80 patients who underwent them. Dysphagia symptoms are significantly associated with EoE. Thirty-three percent of dysphagia patients had EoE, versus 16% of non-dysphagia patients (p < 0.001). Screening hEDS/JHS patients for GI issues should be routine, with further investigations and referrals guided by identified symptoms.<ovid:br/>Copyright &#xa9; 2024 The Authors. Journal of Pediatric Gastroenterology and Nutrition published by Wiley Periodicals LLC on behalf of European Society for Pediatric Gastroenterology, Hepatology, and Nutrition and North American Society for Pediatric Gastroenterology, Hepatology, and Nutrition.

https://onlinelibrary.wiley.com/journal/15364801

http://ovidsp.ovid.com/ovidweb.cgi?T=JS&PAGE=reference&D=emed26&NEWS=N&AN=2029837471

https://ovidsp.ovid.com/ovidweb.cgi?T=JS&CSC=Y&NEWS=N&PAGE=fulltext&D=emed26&DO=10.1002%2fjpn3.12245

http://sfxhosted.exlibrisgroup.com/mayo?sid=OVID:embase&id=pmid:38747032&id=doi:10.1002%2Fjpn3.12245&issn=0277-2116&isbn=&volume=79&issue=1&spage=42&pages=42-47&date=2024&title=Journal+of+Pediatric+Gastroenterology+and+Nutrition&atitle=High+prevalence+of+gastrointestinal+disorders+in+a+large+cohort+of+patients+with+joint+hypermobility&aulast=Sood&pid=%3Cauthor%3ESood+V.%3C%2Fauthor%3E&%3CAN%3E2029837471%3C%2FAN%3E

**17. An exploration of the journey to diagnosis of Ehlers-Danlos Syndrome (EDS) for women living in Australia**

Trudgian, R. and T. Flood (2024). "An exploration of the journey to diagnosis of Ehlers-Danlos Syndrome (EDS) for women living in Australia." PLoS ONE 19(7 July): e0307574.

Introduction Ehlers-Danlos syndromes (EDS) is an umbrella term for a group of hereditary connective tissue disorders usually featuring hyperextensible skin, easy bruising, poor healing, and joint hypermobility. According to international Ehlers-Danlos support groups, the average time to diagnosis of this rare genetic condition is 10-12 years. Consequently, the journey to diagnosis can potentially be traumatic. This pilot study aims to explore female patients' journey to a diagnosis of EDS while living in Australia. Materials and methods Over six weeks, from January to February 2023, a survey was distributed on EDS social media platforms including Facebook and LinkedIn. Ethical approval was obtained through the Ulster University Institute of Nursing and Health Research Ethics Filter Committee (FCNUR). Thematic and statistical analysis of the anonymous data was completed during March 2023. Results 152 women completed the survey. More than half of the respondents first noticed symptoms of EDS more than 15 years prior to diagnosis and more than three quarters of respondents received other diagnoses prior to their EDS diagnosis. Most respondents saw their general practitioner, a physiotherapist and/or a rheumatologist prior to being correctly diagnosed with the condition. While some respondents had positive experiences with these health professionals, many felt that they were not listened to and, after doing their own research, had to educate their health professional/s about EDS. Conclusion This pilot survey demonstrated that the journey to diagnosis of EDS for women in Australia is frequently long and traumatic. Participants indicated that more EDS education and training is needed for health professionals, especially GPs, to improve the diagnostic process. Implications for practice Participants called for their self-reported symptoms to be listened to by health professionals and to be met with empathy and an open mind instead of being dismissed. Additional education and training to health professionals about connective tissue disorders including EDS may aid earlier diagnosis in Australia.<ovid:br/>Copyright &#xa9; 2024 Trudgian, Flood. This is an open access article distributed under the terms of the Creative Commons Attribution License, which permits unrestricted use, distribution, and reproduction in any medium, provided the original author and source are credited.

https://journals.plos.org//plosone/article?id=10.1371/journal.pone.0307574

http://ovidsp.ovid.com/ovidweb.cgi?T=JS&PAGE=reference&D=emed27&NEWS=N&AN=2033510196

https://ovidsp.ovid.com/ovidweb.cgi?T=JS&CSC=Y&NEWS=N&PAGE=fulltext&D=emed27&DO=10.1371%2fjournal.pone.0307574

http://sfxhosted.exlibrisgroup.com/mayo?sid=OVID:embase&id=pmid:39052631&id=doi:10.1371%2Fjournal.pone.0307574&issn=1932-6203&isbn=&volume=19&issue=7+July&spage=e0307574&pages=&date=2024&title=PLoS+ONE&atitle=An+exploration+of+the+journey+to+diagnosis+of+Ehlers-Danlos+Syndrome+%28EDS%29+for+women+living+in+Australia&aulast=Trudgian&pid=%3Cauthor%3ETrudgian+R.%3C%2Fauthor%3E&%3CAN%3E2033510196%3C%2FAN%3E

**18. Clinical diagnosis of the monogenic Ehlers-Danlos syndromes**

Van Dijk, F. S., et al. (2024). "Clinical diagnosis of the monogenic Ehlers-Danlos syndromes." Medizinische Genetik 36(4): 225

EP - 234.

Monogenic Ehlers-Danlos syndromes (EDS) are a group of inherited connective tissue conditions that are clinically characterised by joint hypermobility, skin hyperextensibility and/or fragility, and generalised tissue fragility. Gene panel testing with massively parallel sequencing is currently gold standard to confirm diagnoses of the monogenic EDS types. We aim to report on the (combination of) clinical features of the monogenic EDS types through text and photographs, to aid clinical diagnosis as despite the significant progress in genetic testing possibilities, a thorough clinical assessment which includes medical history, family history and physical examination remains important in the diagnostic process. In addition, in those cases where no molecular diagnosis is possible, a clinical diagnosis can still guide management and surveillance.<ovid:br/>Copyright &#xa9; 2024 the author(s), published by De Gruyter.

https://www.degruyter.com/journal/key/medgen/html

http://ovidsp.ovid.com/ovidweb.cgi?T=JS&PAGE=reference&D=emexb&NEWS=N&AN=2036608743

https://ovidsp.ovid.com/ovidweb.cgi?T=JS&CSC=Y&NEWS=N&PAGE=fulltext&D=emexb&DO=10.1515%2fmedgen-2024-2060

http://sfxhosted.exlibrisgroup.com/mayo?sid=OVID:embase&id=pmid:&id=doi:10.1515%2Fmedgen-2024-2060&issn=0936-5931&isbn=&volume=36&issue=4&spage=225&pages=225-234&date=2024&title=Medizinische+Genetik&atitle=Clinical+diagnosis+of+the+monogenic+Ehlers-Danlos+syndromes&aulast=Van+Dijk&pid=%3Cauthor%3EVan+Dijk+F.S.%3C%2Fauthor%3E&%3CAN%3E2036608743%3C%2FAN%3E

**19. Ehlers-Danlos syndromes: importance of defining the type**

van Dijk, F. S., et al. (2024). "Ehlers-Danlos syndromes: importance of defining the type." Practical neurology 24(2): 90

EP - 97.

Ehlers-Danlos syndromes (EDS) is an umbrella term describing 14 types, of which 13 are rare and monogenic, with overlapping features of joint hypermobility, skin, and vascular fragility, and generalised connective tissue friability. Hypermobile EDS currently has no identified genetic cause. Most of the rare monogenic EDS types can have neurological features, which are often part of major or minor diagnostic criteria for each type. This review aims to highlight the neurological features and other key characteristics of these EDS types. This should improve recognition of these features, enabling more timely consideration and confirmation or exclusion through genetic testing. In practice, many healthcare professionals still refer to patients as having 'EDS'. However, the different EDS types have distinct clinical features as well as different underlying genetic causes and pathogenic mechanisms, and each requires bespoke management and surveillance. Defining the EDS type is therefore crucial, as EDS is not in itself a diagnosis.<ovid:br/>Copyright &#xa9; Author(s) (or their employer(s)) 2024. No commercial re-use. See rights and permissions. Published by BMJ.

http://ovidsp.ovid.com/ovidweb.cgi?T=JS&PAGE=reference&D=emed26&NEWS=N&AN=643150379

https://ovidsp.ovid.com/ovidweb.cgi?T=JS&CSC=Y&NEWS=N&PAGE=fulltext&D=emed26&DO=10.1136%2fpn-2023-003703

http://sfxhosted.exlibrisgroup.com/mayo?sid=OVID:embase&id=pmid:38160052&id=doi:10.1136%2Fpn-2023-003703&issn=1474-7766&isbn=&volume=24&issue=2&spage=90&pages=90-97&date=2024&title=Practical+neurology&atitle=Ehlers-Danlos+syndromes%3A+importance+of+defining+the+type&aulast=van+Dijk&pid=%3Cauthor%3Evan+Dijk+F.S.%3C%2Fauthor%3E&%3CAN%3E643150379%3C%2FAN%3E

**20. Diagnosis and treatment of the Ehlers-Danlos syndromes in China: synopsis of the first guidelines**

Xu, K., et al. (2024). "Diagnosis and treatment of the Ehlers-Danlos syndromes in China: synopsis of the first guidelines." Orphanet Journal of Rare Diseases 19(1): 194.

Background: The Ehlers-Danlos syndromes (EDS) are a group of rare hereditary connective tissue disorders. EDS is clinically and genetically heterogeneous and usually involves multiple systems. There are 14 subtypes of EDS with hallmark features including joint hypermobility, skin hyperextensibility, and tissue fragility. The clinical manifestations and their severity differ among the subtypes, encompassing recurrent joint dislocations, scoliosis, arterial aneurysm and dissection, and organ rupture. Challenges in diagnosis and management arise from the complexity of the disease, which is further complicated by its rarity. The development of clinical guidelines and implementation of coordinated multi-disciplinary team (MDT) approaches have emerged as global priorities. Main body: Chinese Multi-Disciplinary Working Group on the Ehlers-Danlos Syndromes was therefore established. Healthcare professionals were recruited from 25 top hospitals across China. The experts are specialized in 24 fields, including genetics, vascular surgery, dermatology, and orthopedics, as well as nursing care, rehabilitation, psychology, and nutrition. Based on GRADE methodology, the Guidelines were written by the Group supervised by methodologists, following a systemic review of all 4453 articles in PubMed published before August 9, 2023, using the search term "Ehlers Danlos". A coordinated MDT approach for the diagnosis and management of EDS is highly recommended by the Group, along with 29 specific recommendations addressing key clinical questions. In addition to the treatment plan, the Guidelines also emphasize integrating support from nursing care, rehabilitation, psychology, and nutrition. This integration not only facilitates recovery in hospital settings, but most importantly, the transition from an illness-defined life to a more "normalized" life. <ovid:br/>Conclusion(s): The first guidelines on EDS will shorten the diagnostic odyssey and solve the unmet medical needs of the patients. This article is a synopsis of the full guidelines.<ovid:br/>Copyright &#xa9; The Author(s) 2024.

https://ojrd.biomedcentral.com/

http://ovidsp.ovid.com/ovidweb.cgi?T=JS&PAGE=reference&D=emed26&NEWS=N&AN=2029833157

https://ovidsp.ovid.com/ovidweb.cgi?T=JS&CSC=Y&NEWS=N&PAGE=fulltext&D=emed26&DO=10.1186%2fs13023-024-03121-0

http://sfxhosted.exlibrisgroup.com/mayo?sid=OVID:embase&id=pmid:38741208&id=doi:10.1186%2Fs13023-024-03121-0&issn=1750-1172&isbn=&volume=19&issue=1&spage=194&pages=&date=2024&title=Orphanet+Journal+of+Rare+Diseases&atitle=Diagnosis+and+treatment+of+the+Ehlers-Danlos+syndromes+in+China%3A+synopsis+of+the+first+guidelines&aulast=Xu&pid=%3Cauthor%3EXu+K.%3C%2Fauthor%3E&%3CAN%3E2029833157%3C%2FAN%3E

**21. Fracture prevalence in children diagnosed with Ehlers-Danlos Syndrome and Generalized Joint Hypermobility**

Yeung, F., et al. (2024). "Fracture prevalence in children diagnosed with Ehlers-Danlos Syndrome and Generalized Joint Hypermobility." Child Abuse and Neglect 153: 106828.

Background: There is limited understanding of the hypothesized association between the Ehlers-Danlos Syndromes (EDS), hypermobility and fractures in children. Despite this, EDS and hypermobility continue to be raised in the legal setting as possible causes of unexplained fractures in infants where there is a concern for physical abuse. Further understanding is needed regarding fractures in children with EDS and hypermobility. <ovid:br/>Objective(s): This study assessed fracture prevalence and characteristics in children diagnosed with EDS and Generalized Joint Hypermobility (GJH). The secondary outcome was fracture prevalence in infants <1 year of age. Participants and setting: Children aged <18 years with EDS or GJH seen in a single-center EDS clinic from April 2017 to December 2021 were included. Diagnoses were based on the 2017 international classification. Exclusion criteria were concurrent medical conditions associated with bone fragility. <ovid:br/>Method(s): This retrospective descriptive study examined variables including fracture history, fracture location, fracture type, age of sustaining fracture, and injury mechanism. Descriptive statistics were used for analysis. <ovid:br/>Result(s): Fracture prevalence was 34.6 % (9/26, 95 % CI [16.3, 52.9]) in the EDS population and 25.4 % (15/59, 95 % CI [14.3, 36.5]) in the GJH population. No fractures occurred in infancy. Most fractures occurred in the limbs. There were no rib or skull fractures. Most fractures were the result of an identifiable injury event. <ovid:br/>Conclusion(s): In a cohort of children with formally diagnosed EDS or GJH, fractures occurred commonly in ambulatory children and generally in the limbs from identifiable events. This study does not support EDS or GJH as a cause of fractures in infancy.<ovid:br/>Copyright &#xa9; 2024 Elsevier Ltd

https://www.sciencedirect.com/science/journal/01452134

http://ovidsp.ovid.com/ovidweb.cgi?T=JS&PAGE=reference&D=emed26&NEWS=N&AN=2032220807

https://ovidsp.ovid.com/ovidweb.cgi?T=JS&CSC=Y&NEWS=N&PAGE=fulltext&D=emed26&DO=10.1016%2fj.chiabu.2024.106828

http://sfxhosted.exlibrisgroup.com/mayo?sid=OVID:embase&id=pmid:38749147&id=doi:10.1016%2Fj.chiabu.2024.106828&issn=0145-2134&isbn=&volume=153&issue=&spage=106828&pages=&date=2024&title=Child+Abuse+and+Neglect&atitle=Fracture+prevalence+in+children+diagnosed+with+Ehlers-Danlos+Syndrome+and+Generalized+Joint+Hypermobility&aulast=Yeung&pid=%3Cauthor%3EYeung+F.%3C%2Fauthor%3E&%3CAN%3E2032220807%3C%2FAN%3E

**22. Genetic diagnosis of the Ehlers-Danlos syndromes**

Zschocke, J., et al. (2024). "Genetic diagnosis of the Ehlers-Danlos syndromes." Medizinische Genetik 36(4): 235

EP - 245.

The Ehlers-Danlos syndromes (EDS) represent a group of genetically diverse disorders characterized by the variable combination of joint hypermobility, hyperextensibility of the skin, and connective tissue fragility affecting the skin and other organs. Based on clinical features, 13 different types of EDS have been delineated, 12 of which represent monogenic conditions caused by pathogenic variants in 21 confirmed genes. Pathogenesis is related to disturbances of collagen formation and/or stability. No monogenic cause has been identified for hypermobile EDS (hEDS), a more common EDS type, which is unlikely to represent a single gene disorder in the majority of affected individuals and at present cannot be diagnosed by genetic investigations. Here we summarize the clinical features and the molecular bases of the monogenic EDS types, highlight diagnostic challenges, and provide guidance for the molecular work-up of affected individuals. In general, genetic tests are indicated if clinical features suggest a monogenic EDS type but are usually unrewarding for other cases of hypermobility.<ovid:br/>Copyright &#xa9; 2024 the author(s), published by De Gruyter.

https://www.degruyter.com/journal/key/medgen/html

http://ovidsp.ovid.com/ovidweb.cgi?T=JS&PAGE=reference&D=emexb&NEWS=N&AN=2036608744

https://ovidsp.ovid.com/ovidweb.cgi?T=JS&CSC=Y&NEWS=N&PAGE=fulltext&D=emexb&DO=10.1515%2fmedgen-2024-2061

http://sfxhosted.exlibrisgroup.com/mayo?sid=OVID:embase&id=pmid:&id=doi:10.1515%2Fmedgen-2024-2061&issn=0936-5931&isbn=&volume=36&issue=4&spage=235&pages=235-245&date=2024&title=Medizinische+Genetik&atitle=Genetic+diagnosis+of+the+Ehlers-Danlos+syndromes&aulast=Zschocke&pid=%3Cauthor%3EZschocke+J.%3C%2Fauthor%3E&%3CAN%3E2036608744%3C%2FAN%3E

**23. The concomitant diagnosis of fibromyalgia and connective tissue disorders: A systematic review**

Alsiri, N., et al. (2023). "The concomitant diagnosis of fibromyalgia and connective tissue disorders: A systematic review." Seminars in Arthritis and Rheumatism 58: 152127.

Background: Anecdotally, fibromyalgia syndrome (FMS) and connective tissue disorders (hypermobile Ehlers-Danlos Syndrome (hEDS), Hypermobility Spectrum disorders (HSD) and Generalized Joint Hypermobility (GJH)) manifest overlap in their diagnostic approach and symptomatic features. Understanding this overlap is important for accurate diagnosis and the success of subsequent management. This study therefore aimed to identify the prevalence of concomitant diagnosis of FMS and hEDS/HSD/GJH in adults and their shared symptomatic manifestations using a systematic review. <ovid:br/>Method(s): MEDLINE (via EBSCO host) was systematically searched. Observational research (case-control or single group) studies were considered for inclusion, where adults screened for hEDS/HSD/GJH and FMS were compared in terms of diagnostic prevalence, and musculoskeletal and non-musculoskeletal manifestations. Studies on pediatric populations were excluded. The quality of the included studies was assessed using the National Institute of Health Quality Assessment of Case-Control Studies and Jonna Briggs Critical Appraisal checklist for prevalence studies. The review was registered prospectively in PROSPERO (CRD42020216283). <ovid:br/>Finding(s): The review included eleven studies: nine case-control studies and two single group studies. The prevalence of concomitant diagnosis of hEDS/HSD and FMS ranged from 68%-88.9% and from 8.0 to 64.2% for GJH and FMS. The prevalence and severity of a range of objective and patient-reported features were similar between hEDS/HSD and FMS, including joint pain (duration, persistence, SF-36-pain component score); joint swelling; muscle weakness; neurological problems; multidimensional pain inventory-activity; dysautonomia and total autonomic symptoms burden (including orthostatic intolerance, reflex syncope, vasomotor, gastrointestinal, diarrhea, constipation and pupillomotor domains); function; and quality of life. Shared symptomatic features between GJH and FMS were mean pain level, tender points count, total myalgia score and psychological impact. <ovid:br/>Interpretation(s): There may be overlapping symptomatology and diagnostic prevalence of FMS and hEDS/HSD/GJH. Clinicians should consider both diagnoses to ensure appropriate diagnosis and management.<ovid:br/>Copyright &#xa9; 2022 Elsevier Inc.

http://www.elsevier.com/inca/publications/store/6/2/3/3/3/2/index.htt

http://ovidsp.ovid.com/ovidweb.cgi?T=JS&PAGE=reference&D=emed25&NEWS=N&AN=2021405976

https://ovidsp.ovid.com/ovidweb.cgi?T=JS&CSC=Y&NEWS=N&PAGE=fulltext&D=emed25&DO=10.1016%2fj.semarthrit.2022.152127

http://sfxhosted.exlibrisgroup.com/mayo?sid=OVID:embase&id=pmid:36462303&id=doi:10.1016%2Fj.semarthrit.2022.152127&issn=0049-0172&isbn=&volume=58&issue=&spage=152127&pages=&date=2023&title=Seminars+in+Arthritis+and+Rheumatism&atitle=The+concomitant+diagnosis+of+fibromyalgia+and+connective+tissue+disorders%3A+A+systematic+review&aulast=Alsiri&pid=%3Cauthor%3EAlsiri+N.%3C%2Fauthor%3E&%3CAN%3E2021405976%3C%2FAN%3E

**24. An exploratory study of clinical characteristics and gait features of adolescents with hypermobility disorders**

de Koning, L. E., et al. (2023). "An exploratory study of clinical characteristics and gait features of adolescents with hypermobility disorders." Gait and Posture 100: 222

EP - 229.

Background: In adolescents with non-pathological and pathological joint hypermobility, gait deviations have been associated with pain and fatigue. It remains unclear what distinguishes the non-pathological form of joint hypermobility (JH) from pathological forms (i.e. hypermobile Ehlers-Danlos syndrome (hEDS) or hypermobility spectrum disorders (HSD). Our objective was to identify discriminative clinical characteristics and biomechanical gait features between adolescents with hEDS/HSD, JH, and healthy controls (HC). <ovid:br/>Method(s): Thirty-two adolescents were classified into three subgroups (hEDS/HSD=12, JH=5, HC=15). Clinical characteristics (e.g. pain intensity and surface, fatigue, functional disability) were inventoried. The gait pattern was assessed using a three-dimensional, eight-camera VICON MX1.3 motion capture system, operating at a sample rate of 100 Hz (VICON, Oxford, UK). Spatiotemporal parameters, joint angles (sagittal plane), joint work, joint impulse, ground reaction force and gait variability expressed as percentage using Principal Component Analysis (PCA) were assessed and analysed using multivariate analysis. Multivariate analysis data is expressed in mean differences(MD), standard error(SE) and P-values. <ovid:br/>Result(s): The hEDS/HSD-group had significantly higher fatigue score (+51.5 points, p = <0.001) and functional disability (+1.6, p < .001) than the HC-group. Pain intensity was significantly higher in the hEDS/HSD-group than the other subgroups (JH; +37 mm p = .004, HC; +38 mm, p = .001). The hEDS/HSD-group showed significantly more gait variability (JH; +7.2(2.0)% p = .003, HC; + 7.8(1.4)%, p = <0.001) and lower joint work (JH; -0.07(0.03)J/kg, p = .007, HC; - 0.06(0.03)J/kg, p = .013) than the other subgroups. The JH-group showed significantly increased ankle dorsiflexion during terminal stance (+5.0(1.5)degree, p = .001) compared to hEDS/HSD-group and knee flexion during loading response compared to HC-group (+5.7(1.8) degree, p = .011). <ovid:br/>Significance: A distinctive difference in gait pattern between adolescents with non-pathological and pathological joint hypermobility is found in gait variability, rather than in the biomechanical features of gait. This suggests that a specific gait variability metric is more appropriate than biomechanical individual joint patterns for assessing gait in adolescents with hEDS/HSD.<ovid:br/>Copyright &#xa9; 2023 The Authors

https://www.elsevier.com/locate/gaitpost

http://ovidsp.ovid.com/ovidweb.cgi?T=JS&PAGE=reference&D=emed26&NEWS=N&AN=2022160003

https://ovidsp.ovid.com/ovidweb.cgi?T=JS&CSC=Y&NEWS=N&PAGE=fulltext&D=emed26&DO=10.1016%2fj.gaitpost.2023.01.001

http://sfxhosted.exlibrisgroup.com/mayo?sid=OVID:embase&id=pmid:36638668&id=doi:10.1016%2Fj.gaitpost.2023.01.001&issn=0966-6362&isbn=&volume=100&issue=&spage=222&pages=222-229&date=2023&title=Gait+and+Posture&atitle=An+exploratory+study+of+clinical+characteristics+and+gait+features+of+adolescents+with+hypermobility+disorders&aulast=de+Koning&pid=%3Cauthor%3Ede+Koning+L.E.%3C%2Fauthor%3E&%3CAN%3E2022160003%3C%2FAN%3E

**25. Somatic symptoms, pain, catastrophizing and the association with disability among children with heritable connective tissue disorders**

de Koning, L. E., et al. (2023). "Somatic symptoms, pain, catastrophizing and the association with disability among children with heritable connective tissue disorders." American Journal of Medical Genetics, Part A 191(7): 1792

EP - 1803.

The aim of the present study was to investigate the nature and prevalence of nonspecific somatic symptoms, pain and catastrophizing in children with Heritable Connective Tissue Disorders (HCTD), and to determine their association with disability. This observational, multicenter study included 127 children, aged 4-18 years, with Marfan syndrome (MFS) (59%), Loeys-Dietz syndrome (LDS) (8%), Ehlers-Danlos syndromes (EDS) (12%) and hypermobile Ehlers-Danlos syndrome (hEDS) (23%). The assessments included the Children's Somatization Inventory or parent proxy (CSI, PCSI), pain visual-analogue scale (VAS), SUPERKIDZ body diagram, Pain Catastrophizing Scale Child or parent proxy (PCS-C, PCS-P) and Childhood Health Assessment Questionnaire (CHAQ-30). Data from children aged >=8 years were compared to normative data. In children >= 8 years (n = 90), pain was present in 59%, with a median of 4 (IQR = 3-9) pain areas. Compared to normative data, the HCTD group reported significantly higher on the CSI (p <= 0.001, d = 0.85), VAS pain intensity (p <= 0.001, d = 1.22) and CHAQ-30 (p <= 0.001, d = 1.16) and lower on the PCS-C (p = 0.017, d = -0.82) and PCS-P (p <= 0.001, d = -0.49). The intensity of nonspecific somatic symptoms and pain explained 45% of the variance in disability (r<ovid:sup>2</ovid:sup> = 0.45 F(2,48) = 19.70, p <= 0.001). In children <= 7 years (n = 37), pain was present in 35% with a median of 5(IQR = 1-13) pain areas. The mean(SD) VAS scores for pain intensity was 1.5(2.9). Functional disability was moderately correlated to the number of pain areas (r = 0.56, p <= 0.001), intensity of nonspecific somatic symptoms (r = 0.63, p <= 0.001) and pain (r = 0.83, p <= 0.001). In conclusion, this study supports the need for comprehensive assessment of nonspecific somatic symptoms, pain, and disability in children with HCTD to allow tailored treatment.<ovid:br/>Copyright &#xa9; 2023 The Authors. American Journal of Medical Genetics Part A published by Wiley Periodicals LLC.

http://onlinelibrary.wiley.com/journal/10.1002/(ISSN)1552-4833

http://ovidsp.ovid.com/ovidweb.cgi?T=JS&PAGE=reference&D=emed26&NEWS=N&AN=2022859193

https://ovidsp.ovid.com/ovidweb.cgi?T=JS&CSC=Y&NEWS=N&PAGE=fulltext&D=emed26&DO=10.1002%2fajmg.a.63204

http://sfxhosted.exlibrisgroup.com/mayo?sid=OVID:embase&id=pmid:37186039&id=doi:10.1002%2Fajmg.a.63204&issn=1552-4825&isbn=&volume=191&issue=7&spage=1792&pages=1792-1803&date=2023&title=American+Journal+of+Medical+Genetics%2C+Part+A&atitle=Somatic+symptoms%2C+pain%2C+catastrophizing+and+the+association+with+disability+among+children+with+heritable+connective+tissue+disorders&aulast=de+Koning&pid=%3Cauthor%3Ede+Koning+L.E.%3C%2Fauthor%3E&%3CAN%3E2022859193%3C%2FAN%3E

**26. High overlap in patients diagnosed with hypermobile Ehlers-Danlos syndrome or hypermobile spectrum disorders with fibromyalgia and 40 self-reported symptoms and comorbidities**

Fairweather, D., et al. (2023). "High overlap in patients diagnosed with hypermobile Ehlers-Danlos syndrome or hypermobile spectrum disorders with fibromyalgia and 40 self-reported symptoms and comorbidities." Frontiers in Medicine 10: 1096180.

Background: Joint pain is a common symptom in patients with hypermobile Ehlers-Danlos Syndrome (hEDS), hypermobility spectrum disorders (HSD) and fibromyalgia. The goal of this study was to determine whether symptoms and comorbidities overlap in patients diagnosed with hEDS/HSD and/or fibromyalgia. <ovid:br/>Method(s): We retrospectively examined self-reported data from an EDS Clinic intake questionnaire in patients diagnosed with hEDS/HSD, fibromyalgia, or both vs. controls with an emphasis on joint issues. <ovid:br/>Result(s): From 733 patients seen at the EDS Clinic, 56.5% (n = 414) were diagnosed with hEDS/HSD and fibromyalgia (Fibro), 23.8% (n = 167) hEDS/HSD, 13.3% (n = 98) fibromyalgia, or 7.4% (n = 54) none of these diagnoses. More patients were diagnosed with HSD (76.6%) than hEDS (23.4%). Patients were primarily White (95%) and female (90%) with a median age in their 30s (controls 36.7 [18.0, 70.0], fibromyalgia 39.7 [18.0, 75.0], hEDS/HSD 35.0 [18.0, 71.0], hEDS/HSD&Fibro 31.0 [18.0, 63.0]). There was high overlap in all 40 symptoms/comorbidities that we examined in patients diagnosed with fibromyalgia only or hEDS/HSD&Fibro, regardless of whether they had hEDS or HSD. Patients that only had hEDS/HSD without fibromyalgia had far fewer symptoms/comorbidities than patients with hEDS/HSD&Fibro. The top self-reported issues in patients that only had fibromyalgia were joint pain, hand pain when writing or typing, brain fog, joint pain keeping from daily activities, allergy/atopy and headache. Five issues that significantly and uniquely characterized patients diagnosed with hEDS/HSD&Fibro were subluxations (dislocations in hEDS patients), joint issues like sprains, the need to stop sports due to injuries, poor wound healing, and migraine. <ovid:br/>Conclusion(s): The majority of patients seen at the EDS Clinic had a diagnosis of hEDS/HSD plus fibromyalgia that was associated with more severe disease. Our findings indicate that fibromyalgia should be routinely assessed in patients with hEDS/HSD and vis-a-versa to improve patient care.<ovid:br/>Copyright &#xa9; 2023 Fairweather, Bruno, Darakjian, Bruce, Gehin, Kotha, Jain, Peng, Hodge, Rozen, Munipalli, Rivera, Malavet and Knight.

https://journal.frontiersin.org/journal/medicine

http://ovidsp.ovid.com/ovidweb.cgi?T=JS&PAGE=reference&D=emed25&NEWS=N&AN=2023118245

https://ovidsp.ovid.com/ovidweb.cgi?T=JS&CSC=Y&NEWS=N&PAGE=fulltext&D=emed25&DO=10.3389%2ffmed.2023.1096180

http://sfxhosted.exlibrisgroup.com/mayo?sid=OVID:embase&id=pmid:&id=doi:10.3389%2Ffmed.2023.1096180&issn=2296-858X&isbn=&volume=10&issue=&spage=1096180&pages=&date=2023&title=Frontiers+in+Medicine&atitle=High+overlap+in+patients+diagnosed+with+hypermobile+Ehlers-Danlos+syndrome+or+hypermobile+spectrum+disorders+with+fibromyalgia+and+40+self-reported+symptoms+and+comorbidities&aulast=Fairweather&pid=%3Cauthor%3EFairweather+D.%3C%2Fauthor%3E&%3CAN%3E2023118245%3C%2FAN%3E

**27. Pain Characteristics and Symptom Management in Children with Hypermobile Ehlers-Danlos Syndrome and Hypermobility Spectrum Disorder**

Jeong, H. J., et al. (2023). "Pain Characteristics and Symptom Management in Children with Hypermobile Ehlers-Danlos Syndrome and Hypermobility Spectrum Disorder." Physical & occupational therapy in pediatrics 43(5): 630

EP - 643.

AIMS: This study aims to investigate pediatric hypermobile Ehlers-Danlos syndrome (hEDS) and hypermobility spectrum disorder (HSD) pain features and management strategies. <ovid:br/>METHOD(S): This is a mixed-methods, cross-sectional study design using patient-reported outcomes in 21 children diagnosed with hEDS/HSD. Children who reported bothersome pain were interviewed for pain features. The Child Activity Limitation Interview-21, the Brief Pain Inventory pain interference items, and the Functional Disability Inventory were used to investigate pain interference. To evaluate psychological symptoms regarding pain, the pediatric version of the Survey of Pain Attitude and the child version of the Pain Catastrophizing Scale were used. <ovid:br/>RESULT(S): Nineteen children had bothersome pain and of them, eight children reported constant pain. The most frequently reported regions of pain were at the ankle (mild pain) and the back (moderate-to-severe pain). Children reported mild-to-moderate pain interference and believed medications were beneficial for their pain management. Nineteen children sought treatment and of those 16 children used to exercise and acetaminophen and 13 visited physicians as a means of treatment. Parents were overall satisfied with their child's treatment (13 out of 19). <ovid:br/>CONCLUSION(S): Sufficient awareness of pain-related symptoms and understanding of the treatment strategies in early childhood is needed to prevent deleterious consequences in adulthood.

http://ovidsp.ovid.com/ovidweb.cgi?T=JS&PAGE=reference&D=emed25&NEWS=N&AN=640039292

https://ovidsp.ovid.com/ovidweb.cgi?T=JS&CSC=Y&NEWS=N&PAGE=fulltext&D=emed25&DO=10.1080%2f01942638.2022.2163601

http://sfxhosted.exlibrisgroup.com/mayo?sid=OVID:embase&id=pmid:36647261&id=doi:10.1080%2F01942638.2022.2163601&issn=1541-3144&isbn=&volume=43&issue=5&spage=630&pages=630-643&date=2023&title=Physical+%26+occupational+therapy+in+pediatrics&atitle=Pain+Characteristics+and+Symptom+Management+in+Children+with+Hypermobile+Ehlers-Danlos+Syndrome+and+Hypermobility+Spectrum+Disorder&aulast=Jeong&pid=%3Cauthor%3EJeong+H.-J.%3C%2Fauthor%3E&%3CAN%3E640039292%3C%2FAN%3E

**28. Pediatric joint hypermobility: a diagnostic framework and narrative review**

Tofts, L. J., et al. (2023). "Pediatric joint hypermobility: a diagnostic framework and narrative review." Orphanet Journal of Rare Diseases 18(1): 104.

Background: Hypermobile Ehlers-Danlos syndrome (hEDS) and hypermobility spectrum disorders (HSD) are debilitating conditions. Diagnosis is currently clinical in the absence of biomarkers, and criteria developed for adults are difficult to use in children and biologically immature adolescents. Generalized joint hypermobility (GJH) is a prerequisite for hEDS and generalized HSD. Current literature identifies a large proportion of children as hypermobile using a Beighton score >= 4 or 5/9, the cut off for GJH in adults. Other phenotypic features from the 2017 hEDS criteria can arise over time. Finally, many comorbidities described in hEDS/HSD are also seen in the general pediatric and adolescent population. Therefore, pediatric specific criteria are needed. The Paediatric Working Group of the International Consortium on EDS and HSD has developed a pediatric diagnostic framework presented here. The work was informed by a review of the published evidence. Observations: The framework has 4 components, GJH, skin and tissue abnormalities, musculoskeletal complications, and core comorbidities. A Beighton score of >= 6/9 best identifies children with GJH at 2 standard deviations above average, based on published general population data. Skin and soft tissue changes include soft skin, stretchy skin, atrophic scars, stretch marks, piezogenic papules, and recurrent hernias. Two symptomatic groups were agreed: musculoskeletal and systemic. Emerging comorbid relationships are discussed. The framework generates 8 subgroups, 4 pediatric GJH, and 4 pediatric generalized hypermobility spectrum disorders. hEDS is reserved for biologically mature adolescents who meet the 2017 criteria, which also covers even rarer types of Ehlers-Danlos syndrome at any age. <ovid:br/>Conclusion(s): This framework allows hypermobile children to be categorized into a group describing their phenotypic and symptomatic presentation. It clarifies the recommendation that comorbidities should be defined using their current internationally accepted frameworks. This provides a foundation for improving clinical care and research quality in this population.<ovid:br/>Copyright &#xa9; 2023, The Author(s).

https://ojrd.biomedcentral.com/

http://ovidsp.ovid.com/ovidweb.cgi?T=JS&PAGE=reference&D=emed25&NEWS=N&AN=2023066878

https://ovidsp.ovid.com/ovidweb.cgi?T=JS&CSC=Y&NEWS=N&PAGE=fulltext&D=emed25&DO=10.1186%2fs13023-023-02717-2

http://sfxhosted.exlibrisgroup.com/mayo?sid=OVID:embase&id=pmid:37143135&id=doi:10.1186%2Fs13023-023-02717-2&issn=1750-1172&isbn=&volume=18&issue=1&spage=104&pages=&date=2023&title=Orphanet+Journal+of+Rare+Diseases&atitle=Pediatric+joint+hypermobility%3A+a+diagnostic+framework+and+narrative+review&aulast=Tofts&pid=%3Cauthor%3ETofts+L.J.%3C%2Fauthor%3E&%3CAN%3E2023066878%3C%2FAN%3E

**29. Spinal Deformity in Ehlers-Danlos Syndrome: Focus on Musculocontractural Type**

Uehara, M., et al. (2023). "Spinal Deformity in Ehlers-Danlos Syndrome: Focus on Musculocontractural Type." Genes 14(6): 1173.

Spinal deformity in Ehlers-Danlos syndrome (EDS) is an important symptom that can lead to trunk balance deterioration, respiratory dysfunction, and digestive disorders as the deformity progresses, thereby reducing a patient's quality of life and activities of daily living. The severity of the deformity varies widely, with treatment depending on the extent and the presence of associated complications. The present review addressed the current state of clinical research and treatment of spinal deformities in EDS with a specific focus on the musculocontractural type. Further studies are needed to better understand the underlying mechanisms of spinal deformity in EDS.<ovid:br/>Copyright &#xa9; 2023 by the authors.

http://www.mdpi.com/journal/genes/

http://ovidsp.ovid.com/ovidweb.cgi?T=JS&PAGE=reference&D=emed25&NEWS=N&AN=2024103523

https://ovidsp.ovid.com/ovidweb.cgi?T=JS&CSC=Y&NEWS=N&PAGE=fulltext&D=emed25&DO=10.3390%2fgenes14061173

http://sfxhosted.exlibrisgroup.com/mayo?sid=OVID:embase&id=pmid:37372353&id=doi:10.3390%2Fgenes14061173&issn=2073-4425&isbn=&volume=14&issue=6&spage=1173&pages=&date=2023&title=Genes&atitle=Spinal+Deformity+in+Ehlers-Danlos+Syndrome%3A+Focus+on+Musculocontractural+Type&aulast=Uehara&pid=%3Cauthor%3EUehara+M.%3C%2Fauthor%3E&%3CAN%3E2024103523%3C%2FAN%3E

**30. Hypermobility spectrum disorders and irritable bowel syndrome: A nationwide study of 1.6 million adolescents**

Zloof, Y., et al. (2023). "Hypermobility spectrum disorders and irritable bowel syndrome: A nationwide study of 1.6 million adolescents." Journal of Gastroenterology and Hepatology (Australia) 38(12): 2076

EP - 2082.

Background and aim: The association between hypermobility spectrum disorders/hypermobile type Ehlers-Danlos syndrome (HDS/hEDS) and irritable bowel syndrome (IBS) is yet to be clarified. We aimed to assess this association in a national sample of adolescents. <ovid:br/>Method(s): A population-based cross-sectional study included 1 627 345 Israeli adolescents (58% male; mean age 17 years) who were medically assessed before compulsory military service during 1998-2020. Diagnoses of HSD/hEDS and IBS were confirmed by board-certified specialists. The prevalence and odds ratios (ORs) for IBS in adolescents with and without HSD/hEDS were computed. <ovid:br/>Result(s): A total of 4686 adolescents (2553 male) with HSD/hEDS were identified, of whom 71 were diagnosed with IBS (prevalence = 1.5%). Of the 1 621 721 adolescents in the control group, 8751 were diagnosed with IBS (prevalence = 0.5%). Unadjusted logistic regression revealed a significant association between HSD/hEDS and IBS (OR = 2.16 [95% confidence interval, CI, 1.90-2.45]), which persisted in multivariable adjusted models (OR = 2.58 [95% CI, 2.02-3.24]), and in several sensitivity analyses. The association was evident in both male and female adolescents with ORs of 2.60 (95% CI, 1.87-3.49), and 2.46 (95% CI, 1.66-3.49), respectively. The association was accentuated in a sensitivity analysis accounting for other medical and psychiatric comorbidities. <ovid:br/>Conclusion(s): We found a significant association between HSD/hEDS and IBS in both male and female adolescents. Clinical awareness of the association can promote early diagnosis of IBS and appropriate multidisciplinary treatment. Further research is required to identify the common pathological pathways of the conditions and to develop new IBS treatment strategies for people with HSD/hEDS.<ovid:br/>Copyright &#xa9; 2023 Journal of Gastroenterology and Hepatology Foundation and John Wiley & Sons Australia, Ltd.

http://onlinelibrary.wiley.com/journal/10.1111/(ISSN)1440-1746

http://ovidsp.ovid.com/ovidweb.cgi?T=JS&PAGE=reference&D=emed25&NEWS=N&AN=2025244986

https://ovidsp.ovid.com/ovidweb.cgi?T=JS&CSC=Y&NEWS=N&PAGE=fulltext&D=emed25&DO=10.1111%2fjgh.16341

http://sfxhosted.exlibrisgroup.com/mayo?sid=OVID:embase&id=pmid:37658800&id=doi:10.1111%2Fjgh.16341&issn=0815-9319&isbn=&volume=38&issue=12&spage=2076&pages=2076-2082&date=2023&title=Journal+of+Gastroenterology+and+Hepatology+%28Australia%29&atitle=Hypermobility+spectrum+disorders+and+irritable+bowel+syndrome%3A+A+nationwide+study+of+1.6+million+adolescents&aulast=Zloof&pid=%3Cauthor%3EZloof+Y.%3C%2Fauthor%3E&%3CAN%3E2025244986%3C%2FAN%3E

**31. Hypermobility spectrum disorders and active migraine in Israeli adolescents: A nationwide study**

Zloof, Y., et al. (2023). "Hypermobility spectrum disorders and active migraine in Israeli adolescents: A nationwide study." Headache 63(7): 934

EP - 941.

Objective: To assess the association between hypermobility spectrum disorders/hypermobile type Ehlers Danlos Syndrome (HSD/hEDS) and migraine in a national sample of adolescents in Israel. <ovid:br/>Background(s): The association between HSD/hEDS and migraine is unclear, even more so in pediatric populations. <ovid:br/>Method(s): This population-based, cross-sectional study included 1,627,345 Israeli adolescents (945,519/1,626,407 [58%] males; mean age 17 +/- 0.5 years) who were medically assessed before mandatory military service during 1998-2020. Diagnoses of migraine with at least one attack per month (active migraine) and HSD/hEDS were confirmed by certified specialists. The prevalences of active migraine in adolescents with and without HSD/hEDS were computed and the association between HSD/hEDS and active migraine was examined. <ovid:br/>Result(s): Active migraine was significantly more prevalent in adolescents with HSD/hEDS (307/4686 [6.5%]) compared to those without HSD/hEDS (51,931/1,621,721 [3.2%]) (OR = 2.16, 95% CI 1.90-2.45). The association between HSD/hEDS and active migraine persisted in a multivariable analysis (OR = 2.08, 95% CI 1.85-2.34) and in several sensitivity analyses. <ovid:br/>Conclusion(s): We found a significant association between HSD/hEDS and active migraine in both male and female adolescents. Clinical awareness of the association can promote early diagnosis and treatment of migraine. Further research is required to identify appropriate pharmacologic and nonpharmacologic migraine treatment strategies for individuals with HSD/hEDS.<ovid:br/>Copyright &#xa9; 2023 The Authors. Headache: The Journal of Head and Face Pain published by Wiley Periodicals LLC on behalf of American Headache Society.

http://onlinelibrary.wiley.com/journal/10.1111/(ISSN)1526-4610

http://ovidsp.ovid.com/ovidweb.cgi?T=JS&PAGE=reference&D=emed26&NEWS=N&AN=2023803733

https://ovidsp.ovid.com/ovidweb.cgi?T=JS&CSC=Y&NEWS=N&PAGE=fulltext&D=emed26&DO=10.1111%2fhead.14526

http://sfxhosted.exlibrisgroup.com/mayo?sid=OVID:embase&id=pmid:37313588&id=doi:10.1111%2Fhead.14526&issn=0017-8748&isbn=&volume=63&issue=7&spage=934&pages=934-941&date=2023&title=Headache&atitle=Hypermobility+spectrum+disorders+and+active+migraine+in+Israeli+adolescents%3A+A+nationwide+study&aulast=Zloof&pid=%3Cauthor%3EZloof+Y.%3C%2Fauthor%3E&%3CAN%3E2023803733%3C%2FAN%3E

**32. Case report: Lingering post-concussive symptoms in a pediatric patient with presumed Ehlers-Danlos syndrome**

Curry, T. M., et al. (2022). "Case report: Lingering post-concussive symptoms in a pediatric patient with presumed Ehlers-Danlos syndrome." Frontiers in Pediatrics 10: 937223.

Background: Connective tissue disorders such as Ehlers-Danlos Syndrome (EDS) can affect collagen and elastin content and structure, including weakening of tissues and vasculature, thus contributing to multiple systemic manifestations. Prior research has successfully focused on peripheral life-threatening manifestations resulting in increased life expectancy, yet clinical observations have warranted investigation of neurological vulnerability, where little is known. Compromised brain tissues and cerebrovasculature could leave these patients vulnerable to mild traumatic brain injury (TBI), with increased severity and duration of post-concussive symptoms and delayed recovery. Clinical reports in adults indicate that higher severity of symptoms after a mild TBI, such as a concussion, can unmask connective tissues disorders leading toward diagnosis. This clinical case report is an example of a pediatric patient with presumed Ehlers-Danlos syndrome who demonstrates increased vulnerability to mild TBI/concussion. <ovid:br/>Patient(s): A pediatric female patient presents with unexplained lingering post-concussive symptoms, including trouble sleeping, nausea, frontal headaches, dizziness, visual changes, fatigue, and left-sided weakness more than 6 months post-mild concussion. Patient history of hypermobility, joint derangement, soft tissue mobility, and bruising suggests a potential diagnosis of Ehlers-Danlos syndrome, which may explain symptom severity and length of recovery. <ovid:br/>Discussion(s): This case is the first documented instance of increased vulnerability to TBI in a pediatric patient with presumed Ehlers-Danlos syndrome. It highlights the need for awareness and prevention of injury in this vulnerable patient population, suggests more targeted therapeutic intervention for recovery, and demonstrates the need for preclinical research evaluating the influence of genetic mutations associated with connective tissue disorders on the central nervous system.<ovid:br/>Copyright 2022 Curry, Esfandiarei, Thomas and Rastogi.

https://www.frontiersin.org/journals/pediatrics

http://ovidsp.ovid.com/ovidweb.cgi?T=JS&PAGE=reference&D=emed24&NEWS=N&AN=2020133265

https://ovidsp.ovid.com/ovidweb.cgi?T=JS&CSC=Y&NEWS=N&PAGE=fulltext&D=emed24&DO=10.3389%2ffped.2022.937223

http://sfxhosted.exlibrisgroup.com/mayo?sid=OVID:embase&id=pmid:&id=doi:10.3389%2Ffped.2022.937223&issn=2296-2360&isbn=&volume=10&issue=&spage=937223&pages=&date=2022&title=Frontiers+in+Pediatrics&atitle=Case+report%3A+Lingering+post-concussive+symptoms+in+a+pediatric+patient+with+presumed+Ehlers-Danlos+syndrome&aulast=Curry&pid=%3Cauthor%3ECurry+T.M.%3C%2Fauthor%3E&%3CAN%3E2020133265%3C%2FAN%3E

**33. Diagnostic outcomes for molecular genetic testing in children with suspected Ehlers-Danlos syndrome**

Damseh, N., et al. (2022). "Diagnostic outcomes for molecular genetic testing in children with suspected Ehlers-Danlos syndrome." American Journal of Medical Genetics, Part A 188(5): 1376

EP - 1383.

Ehlers-Danlos syndrome (EDS) is a heterogeneous group of connective tissue disorders characterized by hyperextensible skin, hypermobile joints, easy bruisability, and fragility of the connective tissues. The diagnosis is based on clinical assessment and phenotype-guided genetic testing. Most EDS subtypes can be confirmed by genetic testing except for hypermobile EDS. This study explored the utility of applying the 2017 EDS classification criteria and molecular genetic testing in establishing an EDS diagnosis in children. In this retrospective study, we reviewed 72 patients referred to a tertiary care center for evaluation of EDS who underwent one or more forms of genetic testing. Eighteen patients (18/72, 25%) met the clinical criteria for one of the EDS subtypes and of these, 15 (15/18, 83%) were confirmed molecularly. Fifty-four patients (54/72, 75%) had features that overlapped EDS and other syndromes associated with joint hypermobility but did not fully meet clinical criteria. Twelve of them (12/54, 22%) were later shown to have a positive molecular genetic diagnosis of EDS. Different molecular genetic tests were performed on the cohort of 72 patients (EDS panel, n = 44; microarray, n = 25; whole exome sequencing [WES], n = 9; single gene sequencing, n = 3; familial variant testing, n = 10; other genetic panels n = 3). EDS panel was completed in 44 patients (61%), and a molecular diagnosis was confirmed in nine of the patients who satisfied criteria for one of the EDS subtypes (9/12, 75%) and in nine of the patients who did not fully meet criteria (9/32, 28%). We observed a correlation between generalized joint hypermobility, poor healing, easy bruising, atrophic scars, skin hyperextensibility, and developmental dysplasia of the hip with a positive molecular result. This study provides guidance for the use of molecular genetic testing in combination with the 2017 clinical diagnostic criteria in children presenting with EDS characteristics.<ovid:br/>Copyright &#xa9; 2022 Wiley Periodicals LLC.

http://onlinelibrary.wiley.com/journal/10.1002/(ISSN)1552-4833

http://ovidsp.ovid.com/ovidweb.cgi?T=JS&PAGE=reference&D=emed23&NEWS=N&AN=2014948085

https://ovidsp.ovid.com/ovidweb.cgi?T=JS&CSC=Y&NEWS=N&PAGE=fulltext&D=emed23&DO=10.1002%2fajmg.a.62672

http://sfxhosted.exlibrisgroup.com/mayo?sid=OVID:embase&id=pmid:35128800&id=doi:10.1002%2Fajmg.a.62672&issn=1552-4825&isbn=&volume=188&issue=5&spage=1376&pages=1376-1383&date=2022&title=American+Journal+of+Medical+Genetics%2C+Part+A&atitle=Diagnostic+outcomes+for+molecular+genetic+testing+in+children+with+suspected+Ehlers-Danlos+syndrome&aulast=Damseh&pid=%3Cauthor%3EDamseh+N.%3C%2Fauthor%3E&%3CAN%3E2014948085%3C%2FAN%3E

**34. What are the most important symptoms to assess in hypermobile Ehlers-Danlos syndrome? A questionnaire study based on the Delphi technique**

Hakimi, A., et al. (2022). "What are the most important symptoms to assess in hypermobile Ehlers-Danlos syndrome? A questionnaire study based on the Delphi technique." Disability and rehabilitation 44(26): 8325

EP - 8331.

PURPOSE: This study aims to determine from questionnaires, submitted to patients with Ehlers-Danlos syndrome hypermobile type (hEDS), what symptoms they perceive as having the most impact on their well-being and, according to them, what symptoms should be assessed. MATERIALS AND METHODS: Three rounds of online questionnaires were conducted following the Delphi method. The first round allowed us to obtain the most important symptoms to assess according to the patients. The second and third round aimed at ranking the categories according to their order of importance. Establishment of a consensus was evaluated using Kendall's coefficient of concordance. <ovid:br/>RESULT(S): A total of 118 responses were analyzed for the first round and 87 for the second and the third round. Ten categories were extracted from the first round. Ranking of the 10 categories in the second round did not reach consensus (W = 0.33, p < 0.001) nor did the four most important categories in the third round (W = 0.43, p < 0.001). However, three categories stand out from ranking: "pain", "fatigue and sleep disorders", and "musculoskeletal disorders". <ovid:br/>CONCLUSION(S): These categories seem to be the most important to assess in patients with hEDS, despite the lack of consensus on this ranking.Implications for rehabilitationPain, fatigue and sleep disorders, and musculoskeletal disorders should be given high consideration in the assessment of patients with hypermobile Ehlers-Danlos syndrome (EDS).The high phenotypic variability in the hypermobile EDS requires individualized assessment for each patient and a multidisciplinary approach.

http://ovidsp.ovid.com/ovidweb.cgi?T=JS&PAGE=reference&D=emed24&NEWS=N&AN=636924502

https://ovidsp.ovid.com/ovidweb.cgi?T=JS&CSC=Y&NEWS=N&PAGE=fulltext&D=emed24&DO=10.1080%2f09638288.2021.2012839

http://sfxhosted.exlibrisgroup.com/mayo?sid=OVID:embase&id=pmid:34927503&id=doi:10.1080%2F09638288.2021.2012839&issn=1464-5165&isbn=&volume=44&issue=26&spage=8325&pages=8325-8331&date=2022&title=Disability+and+rehabilitation&atitle=What+are+the+most+important+symptoms+to+assess+in+hypermobile+Ehlers-Danlos+syndrome%3F+A+questionnaire+study+based+on+the+Delphi+technique&aulast=Hakimi&pid=%3Cauthor%3EHakimi+A.%3C%2Fauthor%3E&%3CAN%3E636924502%3C%2FAN%3E

**35. Resource utilization and multidisciplinary care needs for patients with Ehlers-Danlos syndrome**

Jones, J. T., et al. (2022). "Resource utilization and multidisciplinary care needs for patients with Ehlers-Danlos syndrome." Molecular genetics & genomic medicine 10(11): e2057.

BACKGROUND: Ehlers-Danlos syndrome (EDS) represents a family of heritable connective tissue disorders with overlapping phenotypic features, frequently including joint hypermobility, tissue fragility, and skin hyperextensibility. Comorbid symptoms are common for patients with EDS and include multiple body systems marked by neurologic, cardiovascular, gastrointestinal, musculoskeletal issues, chronic pain, headaches, and anxiety and depression. The many comorbidities lead to high disease burden, which requires greater healthcare utilization. <ovid:br/>METHOD(S): This survey of families examines healthcare utilization, of adults and minors, through evaluation of subspecialty care appointments across many healthcare systems in one region. <ovid:br/>RESULT(S): There were 155 adults and 83 minors with a diagnosis of EDS with a total of 693 unique visits across 27 different specialties at over 20 different hospitals or clinics in the surveyed area. Cardiology, neurology, and gastroenterology were the most utilized subspecialties for adults, while rheumatology, cardiology, and neurology were most utilized by minors. Many respondents (67%) reported their medical care needs are not being met, and 87% reported interest in a multidisciplinary clinic for EDS with the most interest in pain management, physical and occupational therapy, and rheumatology. <ovid:br/>CONCLUSION(S): Understanding healthcare utilization and needs of those with EDS can provide the foundation for improved care for those with EDS through a coordinated multidisciplinary care model.<ovid:br/>Copyright &#xa9; 2022 The Authors. Molecular Genetics & Genomic Medicine published by Wiley Periodicals LLC.

http://ovidsp.ovid.com/ovidweb.cgi?T=JS&PAGE=reference&D=emed24&NEWS=N&AN=639098559

https://ovidsp.ovid.com/ovidweb.cgi?T=JS&CSC=Y&NEWS=N&PAGE=fulltext&D=emed24&DO=10.1002%2fmgg3.2057

http://sfxhosted.exlibrisgroup.com/mayo?sid=OVID:embase&id=pmid:36151850&id=doi:10.1002%2Fmgg3.2057&issn=2324-9269&isbn=&volume=10&issue=11&spage=e2057&pages=e2057&date=2022&title=Molecular+genetics+%26+genomic+medicine&atitle=Resource+utilization+and+multidisciplinary+care+needs+for+patients+with+Ehlers-Danlos+syndrome&aulast=Jones&pid=%3Cauthor%3EJones+J.T.%3C%2Fauthor%3E&%3CAN%3E639098559%3C%2FAN%3E

**36. Pathologic Skull Fracture in a Near-Term Neonate with Arthrochalasia Type Ehlers-Danlos Syndrome: A Case Report**

Liu, Y. A., et al. (2022). "Pathologic Skull Fracture in a Near-Term Neonate with Arthrochalasia Type Ehlers-Danlos Syndrome: A Case Report." Fetal and Pediatric Pathology 41(1): 149

EP - 154.

Background: Arthrochalasia type Ehlers-Danlos Syndrome (EDS) is a connective tissue disease characterized by severe generalized joint hypermobility, congenital bilateral hip dislocations, and recurrent joint subluxations and dislocations. Only one study has reported bone fragility resulting in fractures. The genetic abnormality underlying this disorder is a variant in the COL1A1 gene causing entire or partial loss of exon 6, resulting in defective type 1 collagen synthesis. Case Report: We report a female infant born at 35 weeks of gestation presenting with pathologic skull fracture following vaginal delivery. Genetic testing revealed a pathogenic variant in the COL1A1 gene (c.472-1G > C), consistent with arthrochalasia type EDS, reported previously. <ovid:br/>Conclusion(s): This report adds pathologic fractures to the phenotypic breadth of this type of EDS and reinforces the importance of including the condition on the differential diagnosis when early onset non-accidental injury or trauma is being considered.<ovid:br/>Copyright &#xa9; 2020 Taylor & Francis Group, LLC.

http://www.tandfonline.com/loi/ipdp20

http://ovidsp.ovid.com/ovidweb.cgi?T=JS&PAGE=reference&D=emed24&NEWS=N&AN=2004863707

https://ovidsp.ovid.com/ovidweb.cgi?T=JS&CSC=Y&NEWS=N&PAGE=fulltext&D=emed24&DO=10.1080%2f15513815.2020.1753269

http://sfxhosted.exlibrisgroup.com/mayo?sid=OVID:embase&id=pmid:32338564&id=doi:10.1080%2F15513815.2020.1753269&issn=1551-3815&isbn=&volume=41&issue=1&spage=149&pages=149-154&date=2022&title=Fetal+and+Pediatric+Pathology&atitle=Pathologic+Skull+Fracture+in+a+Near-Term+Neonate+with+Arthrochalasia+Type+Ehlers-Danlos+Syndrome%3A+A+Case+Report&aulast=Liu&pid=%3Cauthor%3ELiu+Y.A.%3C%2Fauthor%3E&%3CAN%3E2004863707%3C%2FAN%3E

**37. Craniocervical instability in patients with Ehlers-Danlos syndrome: controversies in diagnosis and management**

Mao, G., et al. (2022). "Craniocervical instability in patients with Ehlers-Danlos syndrome: controversies in diagnosis and management." Spine Journal 22(12): 1944

EP - 1952.

Ehlers-Danlos syndrome (EDS) is a rare hereditary condition that can result in ligamentous laxity and hypermobility of the cervical spine. A subset of patients can develop clinical instability of the craniocervical junction associated with pain and neurological dysfunction, potentially warranting treatment with occipitocervical fixation (OCF). Surgical decision-making in patients with EDS can be complicated by difficulty distinguishing from hypermobility inherent in the disease and true pathological instability necessitating intervention. Here we comprehensively review the available medical literature to critically appraise the evidence behind various proposed definitions of instability in the EDS population, and summarize the available outcomes data after OCF. Several radiographic parameters have been used, including the clivo-axial angle, basion-axial interval, and pB-C2 measurement. Despite increasing recognition of EDS by spine surgeons, there remains a paucity of data supporting proposed radiographic parameters for spinal instability among EDS patients. Furthermore, there is a lack of high-quality evidence concerning the efficacy of surgical treatments for chronic debilitating pain prevalent in this population. More standardized clinical measures and rigorous study methodologies are needed to elucidate the role of surgical intervention in this complex patient population.<ovid:br/>Copyright &#xa9; 2022 Elsevier Inc.

http://www.elsevier.com/locate/spinee

http://ovidsp.ovid.com/ovidweb.cgi?T=JS&PAGE=reference&D=emed23&NEWS=N&AN=2020399221

https://ovidsp.ovid.com/ovidweb.cgi?T=JS&CSC=Y&NEWS=N&PAGE=fulltext&D=emed23&DO=10.1016%2fj.spinee.2022.08.008

http://sfxhosted.exlibrisgroup.com/mayo?sid=OVID:embase&id=pmid:36028216&id=doi:10.1016%2Fj.spinee.2022.08.008&issn=1529-9430&isbn=&volume=22&issue=12&spage=1944&pages=1944-1952&date=2022&title=Spine+Journal&atitle=Craniocervical+instability+in+patients+with+Ehlers-Danlos+syndrome%3A+controversies+in+diagnosis+and+management&aulast=Mao&pid=%3Cauthor%3EMao+G.%3C%2Fauthor%3E&%3CAN%3E2020399221%3C%2FAN%3E

**38. Feasibility of video-based joint hypermobility assessment in individuals with suspected Ehlers-Danlos syndromes/generalised hypermobility spectrum disorders: a single-site observational study protocol**

Mittal, N., et al. (2022). "Feasibility of video-based joint hypermobility assessment in individuals with suspected Ehlers-Danlos syndromes/generalised hypermobility spectrum disorders: a single-site observational study protocol." BMJ open 12(12): e068098.

INTRODUCTION: Ehlers-Danlos syndromes (EDS)/generalised hypermobility spectrum disorders (G-HSD) affect the connective tissue of the body and present with a heterogeneous set of symptoms that pose a challenge for diagnosis. One of the main diagnostic criteria of EDS/G-HSD is generalised joint hypermobility, which is currently assessed by clinicians during a physical exam. However, the practice for measuring joint hypermobility is inconsistent between clinicians, leading to high inter-rater variability. Often patients are misdiagnosed with EDS/G-HSD based on an incorrect hypermobility assessment, leading to increased referral rates and resource utilisation at specialised EDS clinics that results in unnecessary emotional distress for patients. An objective, validated and scalable method for assessing hypermobility might mitigate these issues and result in improved EDS/G-HSD patient care. METHODS AND ANALYSIS: This study will examine the use of videos obtained using a smartphone camera to assess the range of motion (ROM) and hypermobility of the joints assessed in Beighton score and more (spine, shoulders, elbows, knees, ankles, thumbs and fifth fingers) in individuals with suspected EDS/G-HSD. Short videos of participants will be captured as they undergo a formal assessment of joint hypermobility at the GoodHope EDS Clinic at Toronto General Hospital. Clinicians will measure the ROM at each joint using a clinical-grade goniometer to establish ground truth measurements. Open-source human pose-estimation libraries will be used to extract the locations of key joints from the videos. Deterministic and machine learning systems will be developed and evaluated for estimating the ROM at each joint. Results will be analysed separately for each joint and human pose-estimation library. ETHICS AND DISSEMINATION: This study was approved by the Research Ethics Board of the University Health Network in Toronto on 26 April 2022. Participants will provide written informed consent. Findings from this study will be published in peer-reviewed journals and presented at conferences. TRIAL REGISTRATION NUMBER: NCT05366114.<ovid:br/>Copyright &#xa9; Author(s) (or their employer(s)) 2022. Re-use permitted under CC BY-NC. No commercial re-use. See rights and permissions. Published by BMJ.

http://ovidsp.ovid.com/ovidweb.cgi?T=JS&PAGE=reference&D=emed24&NEWS=N&AN=639809356

https://ovidsp.ovid.com/ovidweb.cgi?T=JS&CSC=Y&NEWS=N&PAGE=fulltext&D=emed24&DO=10.1136%2fbmjopen-2022-068098

http://sfxhosted.exlibrisgroup.com/mayo?sid=OVID:embase&id=pmid:36526308&id=doi:10.1136%2Fbmjopen-2022-068098&issn=2044-6055&isbn=&volume=12&issue=12&spage=e068098&pages=e068098&date=2022&title=BMJ+open&atitle=Feasibility+of+video-based+joint+hypermobility+assessment+in+individuals+with+suspected+Ehlers-Danlos+syndromes%2Fgeneralised+hypermobility+spectrum+disorders%3A+a+single-site+observational+study+protocol&aulast=Mittal&pid=%3Cauthor%3EMittal+N.%3C%2Fauthor%3E&%3CAN%3E639809356%3C%2FAN%3E

**39. Association of mast-cell-related conditions with hypermobile syndromes: a review of the literature**

Monaco, A., et al. (2022). "Association of mast-cell-related conditions with hypermobile syndromes: a review of the literature." Immunologic Research 70(4): 419

EP - 431.

Ehlers-Danlos syndrome (EDS) is a group of related connective tissue disorders consisting of 13 subtypes, each with its own unique phenotypic and genetic variation. The overlap of symptoms and multitude of EDS variations makes it difficult for patients to achieve a diagnosis early in the course of their disease. The most common form, hypermobile type EDS (hEDS) and its variant, hypermobile spectrum disorder (HSD), are correlated with rheumatologic and inflammatory conditions. Evidence is still needed to determine the pathophysiology of hEDS; however, the association among these conditions and their prevalence in hEDS/HSD may be explained through consideration of persistent chronic inflammation contributing to a disruption of the connective tissue. Aberrant mast cell activation has been shown to play a role in disruption of connective tissue integrity through activity of its mediators including histamine and tryptase which affects multiple organ systems resulting in mast cell activation disorders (MCAD). The overlap of findings associated with MCAD and the immune-mediated and rheumatologic conditions in patients with hEDS/HSD may provide an explanation for the relationship among these conditions and the presence of chronic inflammatory processes in these patients. It is clear that a multidisciplinary approach is required for the treatment of patients with EDS. However, it is also important for clinicians to consider the summarized symptoms and MCAD-associated characteristics in patients with multiple complaints as possible manifestations of connective tissue disorders, in order to potentially aid in establishing an early diagnosis of EDS.<ovid:br/>Copyright &#xa9; 2022, The Author(s), under exclusive licence to Springer Science+Business Media, LLC, part of Springer Nature.

http://www.springer.com/humana+press/journal/12026

http://ovidsp.ovid.com/ovidweb.cgi?T=JS&PAGE=reference&D=emed24&NEWS=N&AN=2015846346

https://ovidsp.ovid.com/ovidweb.cgi?T=JS&CSC=Y&NEWS=N&PAGE=fulltext&D=emed24&DO=10.1007%2fs12026-022-09280-1

http://sfxhosted.exlibrisgroup.com/mayo?sid=OVID:embase&id=pmid:35449490&id=doi:10.1007%2Fs12026-022-09280-1&issn=0257-277X&isbn=&volume=70&issue=4&spage=419&pages=419-431&date=2022&title=Immunologic+Research&atitle=Association+of+mast-cell-related+conditions+with+hypermobile+syndromes%3A+a+review+of+the+literature&aulast=Monaco&pid=%3Cauthor%3EMonaco+A.%3C%2Fauthor%3E&%3CAN%3E2015846346%3C%2FAN%3E

**40. The Underrecognized Conditions of Hypermobile Ehlers-Danlos Syndrome and Hypermobility Spectrum Disorders in Women**

Robbins, K. (2022). "The Underrecognized Conditions of Hypermobile Ehlers-Danlos Syndrome and Hypermobility Spectrum Disorders in Women." Nursing for women's health 26(3): 174

EP - 183.

Hypermobile Ehlers-Danlos syndrome and hypermobility spectrum disorders are conditions that disproportionately affect women and can result in morbidity, disability, and a poorer quality of life. These conditions are often not recognized by health care professionals, leading to significant delays in diagnosis, especially in women. Hypermobile Ehlers-Danlos syndrome and hypermobility spectrum disorders are known as complex, multisystemic conditions that are frequently comorbid with autonomic dysfunction, mast cell activation syndrome, and autism. Education of health care professionals, including nurses, on these conditions could lead to earlier diagnosis, with improved management and thus better outcomes for those affected by these conditions.<ovid:br/>Copyright &#xa9; 2022 AWHONN. Published by Elsevier Inc. All rights reserved.

http://ovidsp.ovid.com/ovidweb.cgi?T=JS&PAGE=reference&D=emed24&NEWS=N&AN=637963280

https://ovidsp.ovid.com/ovidweb.cgi?T=JS&CSC=Y&NEWS=N&PAGE=fulltext&D=emed24&DO=10.1016%2fj.nwh.2022.03.005

http://sfxhosted.exlibrisgroup.com/mayo?sid=OVID:embase&id=pmid:35533735&id=doi:10.1016%2Fj.nwh.2022.03.005&issn=1751-486X&isbn=&volume=26&issue=3&spage=174&pages=174-183&date=2022&title=Nursing+for+women%27s+health&atitle=The+Underrecognized+Conditions+of+Hypermobile+Ehlers-Danlos+Syndrome+and+Hypermobility+Spectrum+Disorders+in+Women&aulast=Robbins&pid=%3Cauthor%3ERobbins+K.%3C%2Fauthor%3E&%3CAN%3E637963280%3C%2FAN%3E

**41. Hypermobile Ehlers-Danlos syndrome (hEDS) phenotype in fragile X premutation carriers: case series**

Tassanakijpanich, N., et al. (2022). "Hypermobile Ehlers-Danlos syndrome (hEDS) phenotype in fragile X premutation carriers: case series." Journal of Medical Genetics 59(7): 687

EP - 690.

Background While an association between full mutation CGG-repeat expansions of the Fragile X Mental Retardation 1 (FMR1) gene and connective tissue problems are clearly described, problems in fragile X premutation carriers (fXPCs) CGG-repeat range (55-200 repeats) of the FMR1 gene may be overlooked. Objective To report five FMR1 fXPCs cases with the hypermobile Ehlers-Danlos syndrome (hEDS) phenotype. Methods We collected medical histories and FMR1 molecular measures from five cases who presented with joint hypermobility and loose connective tissue and met inclusion criteria for hEDS. Results Five cases were female and ranged between 16 and 49 years. The range of CGG-repeat allele sizes ranged from 66 to 150 repeats. All had symptoms of hEDS since early childhood. Commonalities in molecular pathogenesis and coexisting conditions between the fXPCs and hEDS are also presented. The premutation can lead to a reduction of fragile X mental retardation protein, which is crucial in maintaining functions of the extracellular matrix-related proteins, particularly matrix metallopeptidase 9 and elastin. Moreover, elevated FMR1 messenger RNA causes sequestration of proteins, which results in RNA toxicity. Conclusion Both hEDS phenotype and premutation involvement may co-occur because of related commonalities in pathogenesis.<ovid:br/>Copyright &#xa9; 2022 BMJ Publishing Group. All rights reserved.

http://jmg.bmj.com/content/by/year

http://ovidsp.ovid.com/ovidweb.cgi?T=JS&PAGE=reference&D=emed23&NEWS=N&AN=635412771

https://ovidsp.ovid.com/ovidweb.cgi?T=JS&CSC=Y&NEWS=N&PAGE=fulltext&D=emed23&DO=10.1136%2fjmedgenet-2020-107609

http://sfxhosted.exlibrisgroup.com/mayo?sid=OVID:embase&id=pmid:34193467&id=doi:10.1136%2Fjmedgenet-2020-107609&issn=0022-2593&isbn=&volume=59&issue=7&spage=687&pages=687-690&date=2022&title=Journal+of+Medical+Genetics&atitle=Hypermobile+Ehlers-Danlos+syndrome+%28hEDS%29+phenotype+in+fragile+X+premutation+carriers%3A+case+series&aulast=Tassanakijpanich&pid=%3Cauthor%3ETassanakijpanich+N.%3C%2Fauthor%3E&%3CAN%3E635412771%3C%2FAN%3E

**42. Hypermobile Ehlers-Danlos syndrome and disorders of the gastrointestinal tract: What the gastroenterologist needs to know**

Thwaites, P. A., et al. (2022). "Hypermobile Ehlers-Danlos syndrome and disorders of the gastrointestinal tract: What the gastroenterologist needs to know." Journal of Gastroenterology and Hepatology (Australia) 37(9): 1693

EP - 1709.

Background and Aim: Hypermobile Ehlers-Danlos syndrome (hEDS) and the hypermobility spectrum disorders (HSD) can be challenging to diagnose and manage. Gastrointestinal symptoms and disorders of gut-brain interaction are common in this cohort and multifactorial in origin. The primary aim of this review is to arm the gastroenterologist with a clinically useful understanding of HSD/hEDS, by exploring the association of gastrointestinal disorders with HSD/hEDS, highlighting current pathophysiological understanding and providing a pragmatic approach to managing these patients. <ovid:br/>Method(s): Literature relevant to the gastrointestinal system and hypermobile Ehlers-Danlos syndrome was systematically searched, critically appraised, and summarized. <ovid:br/>Result(s): Diagnosis is based upon clinical criteria and a genetic basis is yet to be defined. The prevalence of many gut symptoms, including abdominal pain (69% vs 27%, P < 0.0001), postprandial fullness (34% vs 16%, P = 0.01), constipation (73% vs 16%, P < 0.001), and diarrhea (47% vs 9%, P < 0.001) are significantly higher in HSD/hEDS compared with non-HSD/hEDS individuals. Disorders of gut-brain interaction are also common, particularly functional dyspepsia. The pathophysiology of gut symptoms is poorly understood but may involve effects of connective tissue laxity and its functional consequences, and the influence of autonomic dysfunction, medication and comorbid mental health disorders. Awareness is the key to early diagnosis. Management is limited in evidence-base but ideally should include an integrated multidisciplinary approach. <ovid:br/>Conclusion(s): HSD/hEDS is a multisystemic disorder in which gastrointestinal symptoms, particularly related to disorders of gut-brain interaction are common. Deficiencies in knowledge regarding the pathophysiological processes limit evidence-based interventions and remain important areas for future research.<ovid:br/>Copyright &#xa9; 2022 The Authors. Journal of Gastroenterology and Hepatology published by Journal of Gastroenterology and Hepatology Foundation and John Wiley & Sons Australia, Ltd.

http://onlinelibrary.wiley.com/journal/10.1111/(ISSN)1440-1746

http://ovidsp.ovid.com/ovidweb.cgi?T=JS&PAGE=reference&D=emed24&NEWS=N&AN=2018288174

https://ovidsp.ovid.com/ovidweb.cgi?T=JS&CSC=Y&NEWS=N&PAGE=fulltext&D=emed24&DO=10.1111%2fjgh.15927

http://sfxhosted.exlibrisgroup.com/mayo?sid=OVID:embase&id=pmid:35750466&id=doi:10.1111%2Fjgh.15927&issn=0815-9319&isbn=&volume=37&issue=9&spage=1693&pages=1693-1709&date=2022&title=Journal+of+Gastroenterology+and+Hepatology+%28Australia%29&atitle=Hypermobile+Ehlers-Danlos+syndrome+and+disorders+of+the+gastrointestinal+tract%3A+What+the+gastroenterologist+needs+to+know&aulast=Thwaites&pid=%3Cauthor%3EThwaites+P.A.%3C%2Fauthor%3E&%3CAN%3E2018288174%3C%2FAN%3E

**43. The Ehlers-Danlos Syndromes against the Backdrop of Inborn Errors of Metabolism**

Van Damme, T., et al. (2022). "The Ehlers-Danlos Syndromes against the Backdrop of Inborn Errors of Metabolism." Genes 13(2): 265.

The Ehlers-Danlos syndromes are a group of multisystemic heritable connective tissue disorders with clinical presentations that range from multiple congenital malformations, over adolescentonset debilitating or even life-threatening complications of connective tissue fragility, to mild conditions that remain undiagnosed in adulthood. To date, thirteen different EDS types have been recognized, stemming from genetic defects in 20 different genes. While initial biochemical and molecular analyses mainly discovered defects in genes coding for the fibrillar collagens type I, III and V or their modifying enzymes, recent discoveries have linked EDS to defects in non-collagenous matrix glycoproteins, in proteoglycan biosynthesis and in the complement pathway. This genetic heterogeneity explains the important clinical heterogeneity among and within the different EDS types. Generalized joint hypermobility and skin hyperextensibility with cutaneous fragility, atrophic scarring and easy bruising are defining manifestations of EDS; however, other signs and symptoms of connective tissue fragility, such as complications of vascular and internal organ fragility, orocraniofacial abnormalities, neuromuscular involvement and ophthalmological complications are variably present in the different types of EDS. These features may help to differentiate between the different EDS types but also evoke a wide differential diagnosis, including different inborn errors of metabolism. In this narrative review, we will discuss the clinical presentation of EDS within the context of inborn errors of metabolism, give a brief overview of their underlying genetic defects and pathophysiological mechanisms and provide a guide for the diagnostic approach.<ovid:br/>Copyright &#xa9; 2022 by the authors. Licensee MDPI, Basel, Switzerland.

https://www.mdpi.com/2073-4425/13/2/265/pdf

http://ovidsp.ovid.com/ovidweb.cgi?T=JS&PAGE=reference&D=emed24&NEWS=N&AN=2015580778

https://ovidsp.ovid.com/ovidweb.cgi?T=JS&CSC=Y&NEWS=N&PAGE=fulltext&D=emed24&DO=10.3390%2fgenes13020265

http://sfxhosted.exlibrisgroup.com/mayo?sid=OVID:embase&id=pmid:35205310&id=doi:10.3390%2Fgenes13020265&issn=2073-4425&isbn=&volume=13&issue=2&spage=265&pages=&date=2022&title=Genes&atitle=The+Ehlers-Danlos+Syndromes+against+the+Backdrop+of+Inborn+Errors+of+Metabolism&aulast=Van+Damme&pid=%3Cauthor%3EVan+Damme+T.%3C%2Fauthor%3E&%3CAN%3E2015580778%3C%2FAN%3E

**44. Prevalence of joint hypermobility syndromes in pediatric postural orthostatic tachycardia syndrome**

Boris, J. R. and T. Bernadzikowski (2021). "Prevalence of joint hypermobility syndromes in pediatric postural orthostatic tachycardia syndrome." Autonomic Neuroscience: Basic and Clinical 231: 102770.

Introduction: Patients with postural orthostatic tachycardia syndrome (POTS) have been shown to exhibit comorbid joint hypermobility manifested as Ehlers-Danlos syndrome (EDS) or hypermobility spectrum disorder (HSD). The prevalence of EDS and HSD in POTS has been demonstrated in smaller studies combining adult and pediatric patients. We examined a large series of pediatric patients to determine their prevalence in children with POTS. <ovid:br/>Material(s) and Method(s): Patients 18 years old, or less, at initial evaluation at our clinic were included. POTS was diagnosed based on at least six months of frequent debilitating symptoms of orthostatic intolerance, plus a consistent heart rate increase of at least 40 beats per minute without orthostatic hypotension on standing test. Patients with a Beighton score of at least 5/9 plus other systemic findings suggestive of EDS were further evaluated in Connective Tissue Disorders clinics. <ovid:br/>Result(s): There were 362 patients meeting inclusion criteria, of which 82 patients had EDS (22.7%) and 141 patients had HSD (39.0%). Patients with EDS had an earlier median age at symptom onset (12.1 vs. 13.5 years, p=0.004) and longer median symptom duration (2.5 vs. 1.5 years, p=0.0008) compared to patients without hypermobility. <ovid:br/>Conclusion(s): Our evaluation of a large series of pediatric patients with POTS revealed that over one-fifth of patients had EDS and over one-third of patients had HSD. The awareness of the prevalence of comorbidities such as hypermobility disorders may help inform providers diagnosing and caring for these patients.<ovid:br/>Copyright &#xa9; 2020 Elsevier B.V.

https://www.elsevier.com/locate/jans

http://ovidsp.ovid.com/ovidweb.cgi?T=JS&PAGE=reference&D=emed22&NEWS=N&AN=2010518470

https://ovidsp.ovid.com/ovidweb.cgi?T=JS&CSC=Y&NEWS=N&PAGE=fulltext&D=emed22&DO=10.1016%2fj.autneu.2020.102770

http://sfxhosted.exlibrisgroup.com/mayo?sid=OVID:embase&id=pmid:33388686&id=doi:10.1016%2Fj.autneu.2020.102770&issn=1566-0702&isbn=&volume=231&issue=&spage=102770&pages=&date=2021&title=Autonomic+Neuroscience%3A+Basic+and+Clinical&atitle=Prevalence+of+joint+hypermobility+syndromes+in+pediatric+postural+orthostatic+tachycardia+syndrome&aulast=Boris&pid=%3Cauthor%3EBoris+J.R.%3C%2Fauthor%3E&%3CAN%3E2010518470%3C%2FAN%3E

**45. The key role of the dental practitioner in early diagnosis of periodontal ehlers-danlos syndromes: A rare case report of siblings**

Brinkmann, J. C. B., et al. (2021). "The key role of the dental practitioner in early diagnosis of periodontal ehlers-danlos syndromes: A rare case report of siblings." Quintessence International 52(2): 166

EP - 174.

Ehlers-Danlos syndromes (EDS) are a group of diverse hereditary connective tissue disorders. Various EDS subtypes present as different diseases. Periodontitis of early onset is a major criterion of periodontal EDS (pEDS). This article reports the clinical case of two siblings, young adults, who came to the clinic for diagnosis and treatment of periodontal disease. The patients had already been diagnosed with pEDS several months earlier after being referred for genetic testing by a dermatologist. It should be noted that in these siblings pEDS had been misinterpreted for years by health care specialists despite the patients' periodontal disease, which had appeared at the age of 3 years. The subsequent effects of periodontal disease in these patients jeopardized the survival prognosis of their teeth. It may be stated that, in spite of pEDS's status as a rare syndrome, the dental practitioner can play a key role in the early diagnosis by responding appropriately to periodontal manifestations at early stages.<ovid:br/>Copyright &#xa9; 2021 Quintessence Publishing Co., Ltd. All rights reserved.

https://www.quintessence-publishing.com/deu/en/article-download/905611/quintessence-international/2021/02/the-key-role-of-the-dental-practitioner-in-early-diagnosis-of-periodontal-ehlers-danlos-syndromes-a-rare-case-report-of-siblings

http://ovidsp.ovid.com/ovidweb.cgi?T=JS&PAGE=reference&D=emed23&NEWS=N&AN=2013164425

https://ovidsp.ovid.com/ovidweb.cgi?T=JS&CSC=Y&NEWS=N&PAGE=fulltext&D=emed23&DO=10.3290%2fj.qi.a45263

http://sfxhosted.exlibrisgroup.com/mayo?sid=OVID:embase&id=pmid:33433082&id=doi:10.3290%2Fj.qi.a45263&issn=0033-6572&isbn=&volume=52&issue=2&spage=166&pages=166-174&date=2021&title=Quintessence+International&atitle=The+key+role+of+the+dental+practitioner+in+early+diagnosis+of+periodontal+ehlers-danlos+syndromes%3A+A+rare+case+report+of+siblings&aulast=Brinkmann&pid=%3Cauthor%3EBrinkmann+J.C.-B.%3C%2Fauthor%3E&%3CAN%3E2013164425%3C%2FAN%3E

**46. Clinical and molecular characteristics of 168 probands and 65 relatives with a clinical presentation of classical Ehlers-Danlos syndrome**

Colman, M., et al. (2021). "Clinical and molecular characteristics of 168 probands and 65 relatives with a clinical presentation of classical Ehlers-Danlos syndrome." Human Mutation 42(10): 1294

EP - 1306.

Classical Ehlers-Danlos syndrome (cEDS) is a heritable connective tissue disorder mainly caused by pathogenic variants in COL5A1 or COL5A2, encoding type V collagen. Its diagnosis, based on clinical criteria and molecular confirmation, can be challenging. We report the molecular and clinical characteristics of 168 probands (72 clinically evaluated at our center) and 65 relatives with a clinical presentation of cEDS. Type V collagen defects were found in 145 probands, 121 (83.5%) were located in COL5A1 and 24 (16.5%) in COL5A2. Although 85.6% of molecularly confirmed patients presented the two major clinical criteria (generalized joint hypermobility, hyperextensible skin with atrophic scarring), significant inter- and intrafamilial phenotypic variability was noted. COL5A2 variants often caused a more severe phenotype. Vascular complications were rare in individuals with type V collagen defects (1.4%). Among the 72 probands clinically evaluated in our center, the mutation detection rate was 82.0%. The majority (68.1%) harbored COL5A1/COL5A2 defects. Yet, 13.9% harbored a defect in another gene (COL1A1, PLOD1, TNXB, AEBP1) highlighting important clinical overlap and the need for molecular confirmation of the diagnosis as this has implications regarding follow-up and genetic counseling. Eighteen percent of the 72 probands remained molecularly unexplained and a COL5A1 variant of unknown significance was identified in 6.9%.<ovid:br/>Copyright &#xa9; 2021 Wiley Periodicals LLC

http://onlinelibrary.wiley.com/journal/10.1002/(ISSN)1098-1004

http://ovidsp.ovid.com/ovidweb.cgi?T=JS&PAGE=reference&D=emed22&NEWS=N&AN=2013281975

https://ovidsp.ovid.com/ovidweb.cgi?T=JS&CSC=Y&NEWS=N&PAGE=fulltext&D=emed22&DO=10.1002%2fhumu.24258

http://sfxhosted.exlibrisgroup.com/mayo?sid=OVID:embase&id=pmid:34265140&id=doi:10.1002%2Fhumu.24258&issn=1059-7794&isbn=&volume=42&issue=10&spage=1294&pages=1294-1306&date=2021&title=Human+Mutation&atitle=Clinical+and+molecular+characteristics+of+168+probands+and+65+relatives+with+a+clinical+presentation+of+classical+Ehlers-Danlos+syndrome&aulast=Colman&pid=%3Cauthor%3EColman+M.%3C%2Fauthor%3E&%3CAN%3E2013281975%3C%2FAN%3E

**47. Hypermobile Ehlers-Danlos syndromes: Complex phenotypes, challenging diagnoses, and poorly understood causes**

Gensemer, C., et al. (2021). "Hypermobile Ehlers-Danlos syndromes: Complex phenotypes, challenging diagnoses, and poorly understood causes." Developmental Dynamics 250(3): 318

EP - 344.

The Ehlers-Danlos syndromes (EDS) are a group of heritable, connective tissue disorders characterized by joint hypermobility, skin hyperextensibility, and tissue fragility. There is phenotypic and genetic variation among the 13 subtypes. The initial genetic findings on EDS were related to alterations in fibrillar collagen, but the elucidation of the molecular basis of many of the subtypes revealed several genes not involved in collagen biosynthesis or structure. However, the genetic basis of the hypermobile type of EDS (hEDS) is still unknown. hEDS is the most common type of EDS and involves generalized joint hypermobility, musculoskeletal manifestations, and mild skin involvement along with the presence of several comorbid conditions. Variability in the spectrum and severity of symptoms and progression of patient phenotype likely depend on age, gender, lifestyle, and expression domains of the EDS genes during development and postnatal life. In this review, we summarize the current molecular, genetic, epidemiologic, and pathogenetic findings related to EDS with a focus on the hypermobile type.<ovid:br/>Copyright &#xa9; 2020 Wiley Periodicals LLC

http://onlinelibrary.wiley.com/journal/10.1002/(ISSN)1097-0177

http://ovidsp.ovid.com/ovidweb.cgi?T=JS&PAGE=reference&D=emed22&NEWS=N&AN=2005882774

https://ovidsp.ovid.com/ovidweb.cgi?T=JS&CSC=Y&NEWS=N&PAGE=fulltext&D=emed22&DO=10.1002%2fdvdy.220

http://sfxhosted.exlibrisgroup.com/mayo?sid=OVID:embase&id=pmid:32629534&id=doi:10.1002%2Fdvdy.220&issn=1058-8388&isbn=&volume=250&issue=3&spage=318&pages=318-344&date=2021&title=Developmental+Dynamics&atitle=Hypermobile+Ehlers-Danlos+syndromes%3A+Complex+phenotypes%2C+challenging+diagnoses%2C+and+poorly+understood+causes&aulast=Gensemer&pid=%3Cauthor%3EGensemer+C.%3C%2Fauthor%3E&%3CAN%3E2005882774%3C%2FAN%3E

**48. Prospective clinical investigations of children with periodontal Ehlers-Danlos syndrome identify generalized lack of attached gingiva as a pathognomonic feature**

Kapferer-Seebacher, I., et al. (2021). "Prospective clinical investigations of children with periodontal Ehlers-Danlos syndrome identify generalized lack of attached gingiva as a pathognomonic feature." Genetics in Medicine 23(2): 316

EP - 322.

Purpose: We report prospective clinical investigations of children affected with periodontal Ehlers-Danlos syndrome (pEDS). The main clinical features of pEDS in adults are early severe periodontitis, generalized lack of attached gingiva, and pretibial hemosiderin plaques due to dominant pathogenic variants in the C1R or C1S genes. <ovid:br/>Method(s): Nineteen children with a parent diagnosed with molecularly confirmed pEDS underwent physical examination including oral and radiological investigations followed by genetic testing. <ovid:br/>Result(s): The only consistent manifestation of pEDS in childhood was a characteristic gingival phenotype: generalized lack of attached gingiva. All children with this gingival phenotype had inherited the familial pathogenic variant (n = 12) whereas the gingival phenotype was absent in children without the familial pathogenic variant (n = 7). Easy bruising was reported in eight affected and zero unaffected children. Other manifestations of pEDS were rarely present in children. Only 2/12 affected children aged 8 and 13 years fulfilled the clinical criteria for pEDS. <ovid:br/>Conclusion(s): Generalized lack of attached gingiva is a pathognomonic feature of pEDS and the only clinical finding that is consistently present in affected adults and children. This is important because an early diagnosis may facilitate better dental hygiene in childhood, which may be essential to prevent early dental loss.<ovid:br/>Copyright &#xa9; 2020, American College of Medical Genetics and Genomics.

http://www.nature.com/gim/index.html

http://ovidsp.ovid.com/ovidweb.cgi?T=JS&PAGE=reference&D=emed22&NEWS=N&AN=2006846362

https://ovidsp.ovid.com/ovidweb.cgi?T=JS&CSC=Y&NEWS=N&PAGE=fulltext&D=emed22&DO=10.1038%2fs41436-020-00985-y

http://sfxhosted.exlibrisgroup.com/mayo?sid=OVID:embase&id=pmid:33005042&id=doi:10.1038%2Fs41436-020-00985-y&issn=1098-3600&isbn=&volume=23&issue=2&spage=316&pages=316-322&date=2021&title=Genetics+in+Medicine&atitle=Prospective+clinical+investigations+of+children+with+periodontal+Ehlers-Danlos+syndrome+identify+generalized+lack+of+attached+gingiva+as+a+pathognomonic+feature&aulast=Kapferer-Seebacher&pid=%3Cauthor%3EKapferer-Seebacher+I.%3C%2Fauthor%3E&%3CAN%3E2006846362%3C%2FAN%3E

**49. Prevalence of adhd and autism spectrum disorder in children with hypermobility spectrum disorders or hypermobile ehlers-danlos syndrome: A retrospective study**

Kindgren, E., et al. (2021). "Prevalence of adhd and autism spectrum disorder in children with hypermobility spectrum disorders or hypermobile ehlers-danlos syndrome: A retrospective study." Neuropsychiatric Disease and Treatment 17: 379

EP - 388.

Introduction: Hypermobility spectrum disorders (HSD) and hypermobile Ehlers-Danlos syndrome (hEDS) are both characterized by generalized hypermobility, in combination with pain, affected proprioception, and pronounced fatigue. Clinical observation indicates that behavioral problems, hyperactivity, and autistic traits are overrepresented in children with those conditions. The purpose of this retrospective study was to establish the prevalence of attention-deficit/hyperactivity disorder (ADHD) and autism spectrum disorder (ASD) among children with HSD and hEDS treated in our clinic since 2012. Subjects and Methods: Since Ehlers-Danlos syndrome (EDS) diagnostic criteria and international classification were changed in 2017, we equate the older diagnosis EDS hypermobility type with the newer hEDS and the older hypermobility syndrome with HSD. A registry search from the computerized medical record system found 201 children (88 boys, 113 girls) aged 6-18 years who were treated at our pediatrics department with the diagnoses HSD or EDS. All medical records (113 with HSD, 88 with EDS) were reviewed, and key symptoms such as fatigue and pain, as well as diagnosis of ADHD/ASD, were recorded. <ovid:br/>Result(s): All EDS cases could be classified as hEDS. Of the entire study cohort, 16% had a verified ADHD diagnosis and a further 7% were undergoing ADHD diagnostic investiga-tion. Significantly more children with hEDS had ADHD compared to children with HSD (p=0.02). In the age group 15-16 years, 35% of those with hEDS had ADHD and, among those aged 17-18 years, ADHD was present in 46%. Children with coexisting ADHD showed a significantly higher proportion of associated symptoms such as fatigue, sleep-problems, and urinary tract problems. ASD had been verified in 6% of the children. Of those with ASD, 92% had sleep problems. <ovid:br/>Conclusion(s): This study shows a strong association between HSD or hEDS and ADHD or ASD. Therefore, children with HSD or hEDS may need to be routinely screened for neuropsychiatric symptoms.<ovid:br/>Copyright &#xa9; 2021 Kindgren et al.

https://www.dovepress.com/getfile.php?fileID=66397

http://ovidsp.ovid.com/ovidweb.cgi?T=JS&PAGE=reference&D=emed22&NEWS=N&AN=2006044194

https://ovidsp.ovid.com/ovidweb.cgi?T=JS&CSC=Y&NEWS=N&PAGE=fulltext&D=emed22&DO=10.2147%2fNDT.S290494

http://sfxhosted.exlibrisgroup.com/mayo?sid=OVID:embase&id=pmid:&id=doi:10.2147%2FNDT.S290494&issn=1178-2021&isbn=&volume=17&issue=&spage=379&pages=379-388&date=2021&title=Neuropsychiatric+Disease+and+Treatment&atitle=Prevalence+of+adhd+and+autism+spectrum+disorder+in+children+with+hypermobility+spectrum+disorders+or+hypermobile+ehlers-danlos+syndrome%3A+A+retrospective+study&aulast=Kindgren&pid=%3Cauthor%3EKindgren+E.%3C%2Fauthor%3E&%3CAN%3E2006044194%3C%2FAN%3E

**50. Rome IV Functional Gastrointestinal Disorders and Health Impairment in Subjects With Hypermobility Spectrum Disorders or Hypermobile Ehlers-Danlos Syndrome**

Lam, C. Y., et al. (2021). "Rome IV Functional Gastrointestinal Disorders and Health Impairment in Subjects With Hypermobility Spectrum Disorders or Hypermobile Ehlers-Danlos Syndrome." Clinical Gastroenterology and Hepatology 19(2): 277

EP - 287.e273.

Background & Aims: Individuals with hypermobility spectrum disorder or hypermobile Ehlers-Danlos Syndrome (HSD/hEDS) are increasingly encountered by gastroenterologists and pose complex clinical challenges. Uncontrolled studies have found functional gastrointestinal disorders (FGIDs) to be common in patients with HSD/hEDS. Some patients have somatic symptoms (medically unexplained symptoms) that might affect FGIDs. We performed a case-control study to determine the prevalence of and factors associated with Rome IV FGIDs in subjects with HSD/hEDS compared with age- and sex- matched population-based controls. <ovid:br/>Method(s): An online general health survey was completed by 603 individuals with HSD/hEDS in October 2018 (cases) and 603 matched individuals from the population of the United Kingdom (controls) in 2015. The mean participant age was 39 yrs, and 96% were women. The survey included questions about Rome IV FGIDs, non-GI and non-musculoskeletal somatic symptoms (maximum number, 10), quality of life, medical history and healthcare use. The prevalence of FGIDs was compared between cases and controls, with subsequent logistic regression models - adjusting for the number of somatic symptoms - used to determine the associations for FGIDs in HSD/hEDS compared with controls. <ovid:br/>Result(s): Nearly all subjects (98%) with HSD/hEDS fulfilled symptom-based criteria for 1 or more Rome IV FGIDs, compared with 47% of controls (P <.0001). The gastrointestinal regions most commonly affected by FGIDs in individuals with HSD/hEDS and control subjects were the bowel (90% vs 40% of controls), gastroduodenal (70% vs 13% of controls), esophageal (56% vs 6% of controls), and anorectal (53% vs 9% of controls); P <.0001. A higher proportion of subjects with HSD/hEDS had FGIDs in 2 or more regions (84% vs 15% of controls; P <.0001). Subjects with HSD/hEDS also reported a significantly higher number of non-GI and non-musculoskeletal somatic symptoms (7.1 vs 3.3 in controls), lower quality of life, and greater healthcare use, including abdominal surgeries and medication use (for example, 84% used analgesics compared with 29% of controls). Almost 40% of subjects with HSD/hEDS reported a diagnosis of chronic fatigue syndrome and/or fibromyalgia. Following adjustments for somatic symptoms, the association for FGIDs in subjects with HSD/hEDS was reduced by as much as 4-fold and in some instances was eliminated. <ovid:br/>Conclusion(s): In a large case-control study of persons with HSD/hEDS, almost all of the cases met criteria for Rome IV FGIDs, incurred considerable health impairment, and had high healthcare use. Patients with HSD/hEDS frequently have somatic symptoms that should be treated to reduce the high burden of gastrointestinal illness in this population.<ovid:br/>Copyright &#xa9; 2021 AGA Institute

http://www.elsevier.com/inca/publications/store/6/7/2/7/4/3/index.htt

http://ovidsp.ovid.com/ovidweb.cgi?T=JS&PAGE=reference&D=emed22&NEWS=N&AN=2010635985

https://ovidsp.ovid.com/ovidweb.cgi?T=JS&CSC=Y&NEWS=N&PAGE=fulltext&D=emed22&DO=10.1016%2fj.cgh.2020.02.034

http://sfxhosted.exlibrisgroup.com/mayo?sid=OVID:embase&id=pmid:32109633&id=doi:10.1016%2Fj.cgh.2020.02.034&issn=1542-3565&isbn=&volume=19&issue=2&spage=277&pages=277-287.e3&date=2021&title=Clinical+Gastroenterology+and+Hepatology&atitle=Rome+IV+Functional+Gastrointestinal+Disorders+and+Health+Impairment+in+Subjects+With+Hypermobility+Spectrum+Disorders+or+Hypermobile+Ehlers-Danlos+Syndrome&aulast=Lam&pid=%3Cauthor%3ELam+C.Y.%3C%2Fauthor%3E&%3CAN%3E2010635985%3C%2FAN%3E

**51. Parental perceptions of bladder dysfunction in children with symptomatic joint hypermobility**

Veriki, M., et al. (2021). "Parental perceptions of bladder dysfunction in children with symptomatic joint hypermobility." American Journal of Medical Genetics, Part C: Seminars in Medical Genetics 187(4): 586

EP - 592.

Children with symptomatic joint hypermobility (SJH) report chronic pain, fatigue, and joint instability as their main symptoms. Symptoms can extend beyond musculoskeletal and include lower urinary tract dysfunction (LUTD). There are no studies exploring parent-perceived quality of life (QoL) in children with LUTD, or strategies used to manage symptoms in this population. Our online cross-sectional survey assessed parents of 6-18-year-olds via two UK charitable organizations. It included the Pediatric Incontinence Questionnaire (PinQ), the Dysfunctional Voiding Symptom Score, and questions exploring treatments and efficacy. Sixty-seven parental questionnaires were analyzed. The majority of children were white females (95%, n = 64). Urgency (97%, n = 65) and enuresis (41.7%, n = 28) were the most frequently reported symptoms. The PinQ questionnaire showed that children were emotionally distressed by LUTD. Twenty-nine parents (43%, n = 29) reported pharmacotherapy as the most effective treatment. Few had accessed other treatments such as physiotherapy (16%, n = 18). Most parents reported that overall treatment did not meet their expectations. This study highlights that parents perceive LUTD as having a great impact on their child's QoL. There is need for education among clinicians about LUTD and hypermobility-related disorders, and for development and evaluation of treatments.<ovid:br/>Copyright &#xa9; 2021 Wiley Periodicals LLC.

http://onlinelibrary.wiley.com/journal/10.1002/(ISSN)1552-4833

http://ovidsp.ovid.com/ovidweb.cgi?T=JS&PAGE=reference&D=emed22&NEWS=N&AN=2014326938

https://ovidsp.ovid.com/ovidweb.cgi?T=JS&CSC=Y&NEWS=N&PAGE=fulltext&D=emed22&DO=10.1002%2fajmg.c.31952

http://sfxhosted.exlibrisgroup.com/mayo?sid=OVID:embase&id=pmid:34845822&id=doi:10.1002%2Fajmg.c.31952&issn=1552-4868&isbn=&volume=187&issue=4&spage=586&pages=586-592&date=2021&title=American+Journal+of+Medical+Genetics%2C+Part+C%3A+Seminars+in+Medical+Genetics&atitle=Parental+perceptions+of+bladder+dysfunction+in+children+with+symptomatic+joint+hypermobility&aulast=Veriki&pid=%3Cauthor%3EVeriki+M.%3C%2Fauthor%3E&%3CAN%3E2014326938%3C%2FAN%3E

**52. Joint hypermobility in children: A neglected sign needing more attention**

Castori, M. (2020). "Joint hypermobility in children: A neglected sign needing more attention." Minerva Pediatrica 72(2): 123

EP - 133.

Joint hypermobility refers to the ability that a joint has to move beyond its normal range of motion. It is common in the general population, particularly in children. While many individuals manifesting joint hypermobility are healthy, this feature can accompany a wide range of symptoms and systemic disorders, whose management can be influenced by their prompt recognition. Given the increasing attention that joint hypermobility is attracting in various fields of medicine, many practitioners are asked to approach more carefully joint hypermobility, in order to avoid over- and under-diagnosis of related disorders. Among the most common conditions featuring joint hypermobility there are hypermobility spectrum disorders and hereditary connective tissue disorders, in particular, the Ehlers-Danlos syndromes. In children, joint hypermobility also accompany a variety of disorders affecting neurodevelopment. The nature of such an association is protean, as joint hypermobility may occur in selected congenital neuromuscular disorders, monogenic multiple malformation/ intellectual disability syndromes, and well-known and emerging genomic syndromes. In addition, joint hypermobility seems strongly associated with developmental coordination disorders. This review offers an overview on definitions, assessment procedures, patterns of associated manifestations and disorders related to joint hypermobility, as well as treatment principles of associated musculoskeletal pain for practitioners that are not familial with this issue but encounter people featuring this physical attribute in their daily activity.<ovid:br/>Copyright &#xa9; 2020 EDIZIONI MINERVA MEDICA.

https://www.minervamedica.it/en/journals/minerva-pediatrica/article.php?cod=R15Y2020N02A0123

http://ovidsp.ovid.com/ovidweb.cgi?T=JS&PAGE=reference&D=emed21&NEWS=N&AN=2006737239

https://ovidsp.ovid.com/ovidweb.cgi?T=JS&CSC=Y&NEWS=N&PAGE=fulltext&D=emed21&DO=10.23736%2fS0026-4946.20.05766-7

http://sfxhosted.exlibrisgroup.com/mayo?sid=OVID:embase&id=pmid:32274910&id=doi:10.23736%2FS0026-4946.20.05766-7&issn=0026-4946&isbn=&volume=72&issue=2&spage=123&pages=123-133&date=2020&title=Minerva+Pediatrica&atitle=Joint+hypermobility+in+children%3A+A+neglected+sign+needing+more+attention&aulast=Castori&pid=%3Cauthor%3ECastori+M.%3C%2Fauthor%3E&%3CAN%3E2006737239%3C%2FAN%3E

**53. The prevalence of the chimeric TNXA/TNXB gene and clinical symptoms of ehlers-danlos syndrome with 21-hydroxylase deficiency**

Gao, Y., et al. (2020). "The prevalence of the chimeric TNXA/TNXB gene and clinical symptoms of ehlers-danlos syndrome with 21-hydroxylase deficiency." Journal of Clinical Endocrinology and Metabolism 105(7): 2288

EP - 2299.

Purpose: Defects in both CYP21A2 and TNXB genes can cause congenital adrenal hyperplasia combined with hypermobility-type Ehlers-Danlos syndrome (EDS), which has recently been named CAH-X syndrome. The purpose of this study is to assess the prevalence of the chimeric TNXA/TNXB gene and clinical symptoms in a Chinese cohort with 21-hydroxylase deficiency (21-OHD). <ovid:br/>Method(s): A total of 424 patients with 21-OHD who were genetically diagnosed were recruited for this study. Multiplex ligation-dependent probe amplification and sequencing were used to identify the CAH-X genotype. Clinical features of joints, skin, and other systems were evaluated in 125 patients. <ovid:br/>Result(s): Ninety-four of the 424 patients had a deletion on at least 1 allele of CYP21A2 and 59 of them harbored the heterozygotic TNXA/TNXB chimera. Frequencies of CAH-X CH-1, CH-2, and CH-3 were 8.2%, 3.1%, and 2.6%, respectively. The incidences of clinical features of EDS were 71.0% and 26.6% in patients with the chimeric TNXA/TNXB genes or without (P < .001). There were statistically significant differences in manifestations among articular (P < .001 in generalized hypermobility) and dermatologic features (P < .001 in hyperextensible skin, P = .015 in velvety skin and P = .033 in poor wound healing). The prevalence of generalized hypermobility was more common in CAH-X CH-2 or CH-3 than CH-1 patients (60% vs 20%, P = .028). <ovid:br/>Conclusion(s): In summary, about 14% of patients with 21-OHD may have chimeric TNXA/TNXB gene mutations in our study and most of them showed EDS-related clinical symptoms. The correlation between CAH-X genotypes and clinical features in connective tissue, like joint or skin, needs to be further investigated.<ovid:br/>Copyright &#xa9; 2020 Endocrine Society. All rights reserved.

https://academic.oup.com/jcem

http://ovidsp.ovid.com/ovidweb.cgi?T=JS&PAGE=reference&D=emed21&NEWS=N&AN=2010072786

https://ovidsp.ovid.com/ovidweb.cgi?T=JS&CSC=Y&NEWS=N&PAGE=fulltext&D=emed21&DO=10.1210%2fclinem%2fdgaa199

http://sfxhosted.exlibrisgroup.com/mayo?sid=OVID:embase&id=pmid:32291442&id=doi:10.1210%2Fclinem%2Fdgaa199&issn=0021-972X&isbn=&volume=105&issue=7&spage=2288&pages=2288-2299&date=2020&title=Journal+of+Clinical+Endocrinology+and+Metabolism&atitle=The+prevalence+of+the+chimeric+TNXA%2FTNXB+gene+and+clinical+symptoms+of+ehlers-danlos+syndrome+with+21-hydroxylase+deficiency&aulast=Gao&pid=%3Cauthor%3EGao+Y.%3C%2Fauthor%3E&%3CAN%3E2010072786%3C%2FAN%3E

**54. Ehlers-Danlos Syndrome in Pregnancy: A Review**

Kang, J., et al. (2020). "Ehlers-Danlos Syndrome in Pregnancy: A Review." European Journal of Obstetrics and Gynecology and Reproductive Biology 255: 118

EP - 123.

Ehlers-Danlos syndrome (EDS) is a group of connective tissue disorders that can result in a range of complications during pregnancy. Pregnant EDS patients generally have a favourable outcome, but those with vascular EDS are more likely to suffer from severe maternal complications. Early diagnosis of EDS and subtype characterization can aid in pre-pregnancy counselling, planning of antenatal care, risk assessment of obstetric and neonatal complications, and influence both obstetric and anaesthetic management of these patients. This piece aims to outline the obstetric implications of classical, hypermobile, and vascular EDS, and review the current literature regarding their optimal obstetric management.<ovid:br/>Copyright &#xa9; 2020 Elsevier B.V.

https://www.elsevier.com/locate/ejogrb

http://ovidsp.ovid.com/ovidweb.cgi?T=JS&PAGE=reference&D=emed21&NEWS=N&AN=2008382292

https://ovidsp.ovid.com/ovidweb.cgi?T=JS&CSC=Y&NEWS=N&PAGE=fulltext&D=emed21&DO=10.1016%2fj.ejogrb.2020.10.033

http://sfxhosted.exlibrisgroup.com/mayo?sid=OVID:embase&id=pmid:33113401&id=doi:10.1016%2Fj.ejogrb.2020.10.033&issn=0301-2115&isbn=&volume=255&issue=&spage=118&pages=118-123&date=2020&title=European+Journal+of+Obstetrics+and+Gynecology+and+Reproductive+Biology&atitle=Ehlers-Danlos+Syndrome+in+Pregnancy%3A+A+Review&aulast=Kang&pid=%3Cauthor%3EKang+J.%3C%2Fauthor%3E&%3CAN%3E2008382292%3C%2FAN%3E

**55. Cardiac involvement in classical or hypermobile Ehlers-Danlos syndrome is uncommon**

Paige, S. L., et al. (2020). "Cardiac involvement in classical or hypermobile Ehlers-Danlos syndrome is uncommon." Genetics in Medicine 22(10): 1583

EP - 1588.

Purpose: Cardiac-valvular and vascular Ehlers-Danlos syndrome (EDS) have significant cardiovascular issues. The prevalence and significance of such abnormalities in classical (cEDS) or hypermobile EDS (hEDS) remain unclear. We report the prevalence of cardiac abnormalities in patients with cEDS and hEDS. <ovid:br/>Method(s): We identified 532 pediatric patients with potential EDS evaluated at our institution from January 2014 through April 2019 by retrospective chart review. Ninety-five patients (12 cEDS and 83 hEDS patients) met 2017 EDS diagnostic criteria and had an echocardiogram. One patient was excluded due to complex congenital heart disease, and two were excluded due to lack of images. We reviewed echocardiograms for all structural abnormalities. <ovid:br/>Result(s): Of these 95 patients, 1 had mild aortic root dilation, and 1 had mild ascending aorta dilation in the setting of a bicuspid aortic valve. Eleven patients (11.6%) had a cardiac valve abnormality, all of which were trivial to mild. None of the patients required cardiac intervention. <ovid:br/>Conclusion(s): Our results demonstrate that aortic dilation and valvular anomalies are uncommon in cEDS or hEDS patients. Given the lack of evidence, we do not recommend echocardiographic evaluation and surveillance in patients with cEDS and hEDS in the absence of clinical findings or positive family history.<ovid:br/>Copyright &#xa9; 2020, American College of Medical Genetics and Genomics.

http://www.nature.com/gim/index.html

http://ovidsp.ovid.com/ovidweb.cgi?T=JS&PAGE=reference&D=emed21&NEWS=N&AN=2005180716

https://ovidsp.ovid.com/ovidweb.cgi?T=JS&CSC=Y&NEWS=N&PAGE=fulltext&D=emed21&DO=10.1038%2fs41436-020-0856-8

http://sfxhosted.exlibrisgroup.com/mayo?sid=OVID:embase&id=pmid:32518415&id=doi:10.1038%2Fs41436-020-0856-8&issn=1098-3600&isbn=&volume=22&issue=10&spage=1583&pages=1583-1588&date=2020&title=Genetics+in+Medicine&atitle=Cardiac+involvement+in+classical+or+hypermobile+Ehlers-Danlos+syndrome+is+uncommon&aulast=Paige&pid=%3Cauthor%3EPaige+S.L.%3C%2Fauthor%3E&%3CAN%3E2005180716%3C%2FAN%3E

**56. Quantitative measures of tissue mechanics to detect hypermobile Ehlers-Danlos syndrome and hypermobility syndrome disorders: a systematic review**

Palmer, S., et al. (2020). "Quantitative measures of tissue mechanics to detect hypermobile Ehlers-Danlos syndrome and hypermobility syndrome disorders: a systematic review." Clinical Rheumatology 39(3): 715

EP - 725.

Hypermobile Ehlers-Danlos syndrome (hEDS) and hypermobility spectrum disorders (HSD) are heritable connective tissue disorders associated with pain, activity limitations and participation restrictions. A key feature is reported to be reduced stiffness and increased extensibility and elasticity of connective tissues. Yet diagnosis relies on assessment of joint range of motion, which may be influenced by other factors, and semi-quantitative assessment of forearm skin extensibility. The objective of this systematic review was to determine if quantitative measures of tissue mechanics can discriminate between hEDS/HSD and healthy tissues. Literature was identified via online databases (AMED, CINAHL+, EMBASE, MEDLINE and SportDiscus) and snowballing. Studies were included if participants had a confirmed diagnosis of hEDS/HSD (or equivalent diagnosis) using internationally recognised criteria, a healthy control group was used as a comparator, and objective measures of tissue stiffness, extensibility or elasticity of muscle, tendon, connective tissue or skin were reported. Included studies were critically appraised, followed by group discussion, consensus and narrative synthesis. Two hundred three potentially relevant studies were identified. Application of the inclusion criteria resulted in four studies being included. A range of quantitative approaches to studying tissue mechanics were used, including diagnostic ultrasound. Overall, three of the four studies found that at least one measure of tissue mechanics distinguished between people with hEDS/HSD and healthy controls. The studies were generally conducted and reported to high standards. Quantitative measures of tissue mechanics have the potential to contribute towards more objective diagnosis of hEDS/HSD. Further validation, particularly within diagnostic scenarios, is required.<ovid:br/>Copyright &#xa9; 2020, The Author(s).

https://link.springer.com/journal/10067

http://ovidsp.ovid.com/ovidweb.cgi?T=JS&PAGE=reference&D=emed21&NEWS=N&AN=2004099574

https://ovidsp.ovid.com/ovidweb.cgi?T=JS&CSC=Y&NEWS=N&PAGE=fulltext&D=emed21&DO=10.1007%2fs10067-020-04939-2

http://sfxhosted.exlibrisgroup.com/mayo?sid=OVID:embase&id=pmid:31955324&id=doi:10.1007%2Fs10067-020-04939-2&issn=0770-3198&isbn=&volume=39&issue=3&spage=715&pages=715-725&date=2020&title=Clinical+Rheumatology&atitle=Quantitative+measures+of+tissue+mechanics+to+detect+hypermobile+Ehlers-Danlos+syndrome+and+hypermobility+syndrome+disorders%3A+a+systematic+review&aulast=Palmer&pid=%3Cauthor%3EPalmer+S.%3C%2Fauthor%3E&%3CAN%3E2004099574%3C%2FAN%3E

**57. Symptomatic joint hypermobility**

Tinkle, B. T. (2020). "Symptomatic joint hypermobility." Best Practice and Research: Clinical Rheumatology 34(3): 101508.

Joint hypermobility is relatively common and has many influences such as age, gender, training, and ethnicity among many. Joint hypermobility may be asymptomatic or symptomatic. It may also be non-syndromic or syndromic. However, "asymptomatic" joint hypermobility may result in repetitive use injury, alter biomechanics of joints at other body sites, or become symptomatic later in life. Symptomatic joint hypermobility can result from soft-tissue rheumatism (e.g. bursitis, tendonitis, etc.) or muscular tension pain due to muscular imbalance. Generalized joint hypermobility (GJH) can be easily assessed using a standardized, quick, in-office examination. Management is relatively straight forward once joint hypermobility is recognized using neuromuscular re-training. It is important to recognize that GJH may also be a feature of a heritable connective tissue disorder with other systemic findings. Therefore, assessing joint hypermobility in those with musculoskeletal complaints may lead to recognizing systemic manifestations and allow the appropriate management.<ovid:br/>Copyright &#xa9; 2020 Elsevier Ltd

http://www.elsevier.com/inca/publications/store/6/2/3/0/0/5/index.htt

http://ovidsp.ovid.com/ovidweb.cgi?T=JS&PAGE=reference&D=emed21&NEWS=N&AN=2005471750

https://ovidsp.ovid.com/ovidweb.cgi?T=JS&CSC=Y&NEWS=N&PAGE=fulltext&D=emed21&DO=10.1016%2fj.berh.2020.101508

http://sfxhosted.exlibrisgroup.com/mayo?sid=OVID:embase&id=pmid:32249022&id=doi:10.1016%2Fj.berh.2020.101508&issn=1521-6942&isbn=&volume=34&issue=3&spage=101508&pages=&date=2020&title=Best+Practice+and+Research%3A+Clinical+Rheumatology&atitle=Symptomatic+joint+hypermobility&aulast=Tinkle&pid=%3Cauthor%3ETinkle+B.T.%3C%2Fauthor%3E&%3CAN%3E2005471750%3C%2FAN%3E

**58. Symptom complaints and impact on functioning in youth with hypermobile Ehlers-Danlos syndrome**

Tran, S. T., et al. (2020). "Symptom complaints and impact on functioning in youth with hypermobile Ehlers-Danlos syndrome." Journal of child health care : for professionals working with children in the hospital and community 24(3): 444

EP - 457.

Hypermobile Ehlers-Danlos syndrome (hEDS), a genetic connective tissue disorder, involves several body systems which makes symptom management and functioning difficult. The aim of this study was to understand pediatric hEDS patients' symptoms and primary complaints. Additionally, we examined the cumulative impact of symptoms on physical and psychological functioning. Thirty-four youth with hEDS were recruited from a genetics clinic and reported the hardest thing about having hEDS, their pain, fatigue, physical symptoms, functional disability, anxiety, and depression. Physical symptoms (pain and fatigue) and limitations (keeping up with friends) were reported as the most difficult parts of having hEDS. A higher number of somatic symptoms was the strongest predictor of disability, anxiety, and depression. Physical symptoms are subjectively distressing and significantly related to impairments in physical and psychological functioning. Thus, addressing these varied symptoms in treatment may yield better functioning in youth with hEDS.

http://ovidsp.ovid.com/ovidweb.cgi?T=JS&PAGE=reference&D=emed21&NEWS=N&AN=629007365

https://ovidsp.ovid.com/ovidweb.cgi?T=JS&CSC=Y&NEWS=N&PAGE=fulltext&D=emed21&DO=10.1177%2f1367493519867174

http://sfxhosted.exlibrisgroup.com/mayo?sid=OVID:embase&id=pmid:31370685&id=doi:10.1177%2F1367493519867174&issn=1741-2889&isbn=&volume=24&issue=3&spage=444&pages=444-457&date=2020&title=Journal+of+child+health+care+%3A+for+professionals+working+with+children+in+the+hospital+and+community&atitle=Symptom+complaints+and+impact+on+functioning+in+youth+with+hypermobile+Ehlers-Danlos+syndrome&aulast=Tran&pid=%3Cauthor%3ETran+S.T.%3C%2Fauthor%3E&%3CAN%3E629007365%3C%2FAN%3E

**59. Pain drawings as a diagnostic tool for the differentiation between two pain-associated rare diseases (Ehlers-Danlos-Syndrome, Guillain-Barre-Syndrome)**

Wester, L., et al. (2020). "Pain drawings as a diagnostic tool for the differentiation between two pain-associated rare diseases (Ehlers-Danlos-Syndrome, Guillain-Barre-Syndrome)." Orphanet Journal of Rare Diseases 15(1): 323.

BACKGROUND: The diagnosis of rare diseases poses a particular challenge to clinicians. This study analyzes whether patients' pain drawings (PDs) help in the differentiation of two pain-associated rare diseases, Ehlers-Danlos Syndrome (EDS) and Guillain-Barre Syndrome (GBS). <ovid:br/>METHOD(S): The study was designed as a prospective, observational, single-center study. The sample comprised 60 patients with EDS (3 male, 52 female, 5 without gender information; 39.2 +/- 11.4 years) and 32 patients with GBS (10 male, 20 female, 2 without gender information; 50.5 +/- 13.7 years). Patients marked areas afflicted by pain on a sketch of a human body with anterior, posterior, and lateral views. PDs were electronically scanned and processed. Each PD was classified based on the Ruzicka similarity to the EDS and the GBS averaged image (pain profile) in a leave-one-out cross validation approach. A receiver operating characteristic (ROC) curve was plotted. <ovid:br/>RESULT(S): 60-80% of EDS patients marked the vertebral column with the neck and the tailbone and the knee joints as pain areas, 40-50% the shoulder-region, the elbows and the thumb saddle joint. 60-70% of GBS patients marked the dorsal and plantar side of the feet as pain areas, 40-50% the palmar side of the fingertips, the dorsal side of the left palm and the tailbone. 86% of the EDS patients and 96% of the GBS patients were correctly identified by computing the Ruzicka similarity. The ROC curve yielded an excellent area under the curve value of 0.95. <ovid:br/>CONCLUSION(S): PDs are a useful and economic tool to differentiate between GBS and EDS. Further studies should investigate its usefulness in the diagnosis of other pain-associated rare diseases. This study was registered in the German Clinical Trials Register, No. DRKS00014777 (Deutsches Register klinischer Studien, DRKS), on 01.06.2018.

http://ovidsp.ovid.com/ovidweb.cgi?T=JS&PAGE=reference&D=emed21&NEWS=N&AN=633453994

https://ovidsp.ovid.com/ovidweb.cgi?T=JS&CSC=Y&NEWS=N&PAGE=fulltext&D=emed21&DO=10.1186%2fs13023-020-01542-1

http://sfxhosted.exlibrisgroup.com/mayo?sid=OVID:embase&id=pmid:33203450&id=doi:10.1186%2Fs13023-020-01542-1&issn=1750-1172&isbn=&volume=15&issue=1&spage=323&pages=323&date=2020&title=Orphanet+journal+of+rare+diseases&atitle=Pain+drawings+as+a+diagnostic+tool+for+the+differentiation+between+two+pain-associated+rare+diseases+%28Ehlers-Danlos-Syndrome%2C+Guillain-Barre-Syndrome%29&aulast=Wester&pid=%3Cauthor%3EWester+L.%3C%2Fauthor%3E&%3CAN%3E633453994%3C%2FAN%3E

**60. Bilateral femoral hernias in a male child as the initial presentation of Ehlers-Danlos syndrome**

Wong, M. and P. J. Javid (2020). "Bilateral femoral hernias in a male child as the initial presentation of Ehlers-Danlos syndrome." Journal of Pediatric Surgery Case Reports 62: 101634.

In this article, we present an unusual case of a young boy who presented with bilateral groin hernias. Surgical exploration ruled out inguinal or direct hernia and subsequent workup including a repeat ultrasound revealed bilateral femoral hernias. Given the patients previous history of joint hypermobility and the rarity of bilateral femoral hernias, the patient was subsequently evaluated for a connective tissue disorder and was ultimately diagnosed with Ehlers-Danlos syndrome (EDS). There are no other such reported cases in the literature. This case highlights the potential association between connective tissue disorders and bilateral femoral hernias in young children.<ovid:br/>Copyright &#xa9; 2020 The Authors

http://www.elsevier.com/journals/journal-of-pediatric-surgery-case-reports/2213-5766

http://ovidsp.ovid.com/ovidweb.cgi?T=JS&PAGE=reference&D=emed21&NEWS=N&AN=2007702659

https://ovidsp.ovid.com/ovidweb.cgi?T=JS&CSC=Y&NEWS=N&PAGE=fulltext&D=emed21&DO=10.1016%2fj.epsc.2020.101634

http://sfxhosted.exlibrisgroup.com/mayo?sid=OVID:embase&id=pmid:&id=doi:10.1016%2Fj.epsc.2020.101634&issn=2213-5766&isbn=&volume=62&issue=&spage=101634&pages=&date=2020&title=Journal+of+Pediatric+Surgery+Case+Reports&atitle=Bilateral+femoral+hernias+in+a+male+child+as+the+initial+presentation+of+Ehlers-Danlos+syndrome&aulast=Wong&pid=%3Cauthor%3EWong+M.%3C%2Fauthor%3E&%3CAN%3E2007702659%3C%2FAN%3E

**61. Severe peripheral joint laxity is a distinctive clinical feature of spondylodysplastic-ehlersdanlos syndrome (Eds)-b4galt7 and spondylodysplastic-eds-b3galt6**

Caraffi, S. G., et al. (2019). "Severe peripheral joint laxity is a distinctive clinical feature of spondylodysplastic-ehlersdanlos syndrome (Eds)-b4galt7 and spondylodysplastic-eds-b3galt6." Genes 10(10): 799.

Variations in genes encoding for the enzymes responsible for synthesizing the linker region of proteoglycans may result in recessive conditions known as "linkeropathies". The two phenotypes related to mutations in genes B4GALT7 and B3GALT6 (encoding for galactosyltransferase I and II respectively) are similar, characterized by short stature, hypotonia, joint hypermobility, skeletal features and a suggestive face with prominent forehead, thin soft tissue and prominent eyes. The most outstanding feature of these disorders is the combination of severe connective tissue involvement, often manifesting in newborns and infants, and skeletal dysplasia that becomes apparent during childhood. Here, we intend to more accurately define some of the clinical features of B4GALT7 and B3GALT6-related conditions and underline the extreme hypermobility of distal joints and the soft, doughy skin on the hands and feet as features that may be useful as the first clues for a correct diagnosis.<ovid:br/>Copyright &#xa9; 2019 by the authors. Licensee MDPI, Basel, Switzerland.

https://www.mdpi.com/2073-4425/10/10/799/pdf

http://ovidsp.ovid.com/ovidweb.cgi?T=JS&PAGE=reference&D=emed20&NEWS=N&AN=2002853922

https://ovidsp.ovid.com/ovidweb.cgi?T=JS&CSC=Y&NEWS=N&PAGE=fulltext&D=emed20&DO=10.3390%2fgenes10100799

http://sfxhosted.exlibrisgroup.com/mayo?sid=OVID:embase&id=pmid:31614862&id=doi:10.3390%2Fgenes10100799&issn=2073-4425&isbn=&volume=10&issue=10&spage=799&pages=&date=2019&title=Genes&atitle=Severe+peripheral+joint+laxity+is+a+distinctive+clinical+feature+of+spondylodysplastic-ehlersdanlos+syndrome+%28Eds%29-b4galt7+and+spondylodysplastic-eds-b3galt6&aulast=Caraffi&pid=%3Cauthor%3ECaraffi+S.G.%3C%2Fauthor%3E&%3CAN%3E2002853922%3C%2FAN%3E

**62. Subjective health complaints and illness perception amongst adults with Joint Hypermobility Syndrome/Ehlers-Danlos Syndrome-HypermobilityType - a cross-sectional study**

Hope, L., et al. (2019). "Subjective health complaints and illness perception amongst adults with Joint Hypermobility Syndrome/Ehlers-Danlos Syndrome-HypermobilityType - a cross-sectional study." Disability and rehabilitation 41(3): 333

EP - 340.

OBJECTIVE: To investigate the prevalence and severity of subjective health complaints and describe illness perception in a population of Joint Hypermobility Syndrome or Ehlers-Danlos Syndrome-Hypermobile Type. <ovid:br/>METHOD(S): This study was a postal survey with a questionnaire battery on demographic data, subjective health complaints inventory, and illness perception. A total of 110 individuals diagnosed with Joint Hypermobility Syndrome or Ehlers-Danlos Syndrome-Hypermobile Type from two specialized hospitals in Norway were offered participation. Further, 140 gender- and age-matched healthy controls from statistics Norway representing the general population were sent the questionnaire for reference. <ovid:br/>RESULT(S): Overall response rate was 30.4% (n = 76), with 44.5% (n = 49) in Joint Hypermobility Syndrome/Ehlers-Danlos Syndrome-Hypermobile Type and 19.3% (n = 27) in controls. Subjective health complaints were significantly higher in Joint Hypermobility Syndrome/Ehlers-Danlos Syndrome-Hypermobile Type - than in the controls (32.06 vs. 11.08; p < 0.001). Further the brief illness perception questionnaire indicated that the adults with Joint Hypermobility Syndrome/Ehlers-Danlos Syndrome-Hypermobile Type had low understanding of their illness and symptoms (understanding, mean: 3.93, SD 2.88), and reported to have moderate personal and treatment control over their illness. <ovid:br/>CONCLUSION(S): Adults with Joint Hypermobility Syndrome/Ehlers-Danlos Syndrome-Hypermobile Type reported higher frequency and severity of subjective health complaints than the matched controls from the general adult population in Norway. Furthermore, Joint Hypermobility Syndrome/Ehlers-Danlos Syndrome-Hypermobile Type reported low understanding of their illness and associated symptoms, and moderate belief that their illness can be kept under control through self-management or treatment. This may indicate one of the reasons why prognosis for these patients is poor. Implications for rehabilitation Awareness of the complexity of the subjective health complaints and inquiry into illness perception could contribute with valuable information about these patients' perceptions of their condition. Such information could in its turn be of value for clinicians as they work towards facilitating a more holistic treatment approach, for example patient education and cognitive behavioural therapy.

http://ovidsp.ovid.com/ovidweb.cgi?T=JS&PAGE=reference&D=emed20&NEWS=N&AN=626356182

https://ovidsp.ovid.com/ovidweb.cgi?T=JS&CSC=Y&NEWS=N&PAGE=fulltext&D=emed20&DO=10.1080%2f09638288.2017.1390695

http://sfxhosted.exlibrisgroup.com/mayo?sid=OVID:embase&id=pmid:29041819&id=doi:10.1080%2F09638288.2017.1390695&issn=1464-5165&isbn=&volume=41&issue=3&spage=333&pages=333-340&date=2019&title=Disability+and+rehabilitation&atitle=Subjective+health+complaints+and+illness+perception+amongst+adults+with+Joint+Hypermobility+Syndrome%2FEhlers-Danlos+Syndrome-HypermobilityType+-+a+cross-sectional+study&aulast=Hope&pid=%3Cauthor%3EHope+L.%3C%2Fauthor%3E&%3CAN%3E626356182%3C%2FAN%3E

**63. Recognizing and Effectively Managing Hypermobility-Related Conditions**

Russek, L. N., et al. (2019). "Recognizing and Effectively Managing Hypermobility-Related Conditions." Physical therapy 99(9): 1189

EP - 1200.

Hypermobility spectrum disorder (HSD) and hypermobile Ehlers-Danlos syndrome (hEDS) can cause widespread or chronic pain, fatigue, and proprioceptive and coordination deficits resulting in functional restrictions. These conditions are common and often unrecognized, and patients are likely to present in physical therapy for musculoskeletal injuries, pain, or coordination deficits. Although physical therapy is considered central to managing these conditions, many patients report pain and iatrogenic injuries due to inappropriate interventions. The diagnostic classification for these conditions was revised in 2017 to supersede previous diagnostic categories of Joint Hypermobility Syndrome and Ehlers-Danlos Syndrome-hypermobility type/type III. It is now known that these conditions affect multiple body systems and not just joints and that patients require a holistic approach. This Perspective article will describe the 2017 diagnostic classification system, clinical presentation, examination, evaluation, and management of patients with HSD/hEDS. Both adult and pediatric cases are presented to illustrate the patient management concepts discussed. This knowledge can lead to more effective management of this patient population.<ovid:br/>Copyright &#xa9; 2019 American Physical Therapy Association.

http://ovidsp.ovid.com/ovidweb.cgi?T=JS&PAGE=reference&D=emed20&NEWS=N&AN=628109077

https://ovidsp.ovid.com/ovidweb.cgi?T=JS&CSC=Y&NEWS=N&PAGE=fulltext&D=emed20&DO=10.1093%2fptj%2fpzz078

http://sfxhosted.exlibrisgroup.com/mayo?sid=OVID:embase&id=pmid:31158283&id=doi:10.1093%2Fptj%2Fpzz078&issn=1538-6724&isbn=&volume=99&issue=9&spage=1189&pages=1189-1200&date=2019&title=Physical+therapy&atitle=Recognizing+and+Effectively+Managing+Hypermobility-Related+Conditions&aulast=Russek&pid=%3Cauthor%3ERussek+L.N.%3C%2Fauthor%3E&%3CAN%3E628109077%3C%2FAN%3E

**64. Prevalence of obstructive sleep apnea in joint hypermobility syndrome: A systematic review and meta-analysis**

Sedky, K., et al. (2019). "Prevalence of obstructive sleep apnea in joint hypermobility syndrome: A systematic review and meta-analysis." Journal of Clinical Sleep Medicine 15(2): 293

EP - 299.

Study Objectives: Because of associated abnormalities affecting connective tissue in various organs including airways, hypermobility syndrome has been associated with high risk for the development sleep apnea. Ehlers-Danlos syndrome (EDS) and Marfan syndrome (MFS) represent the most common hypermobility syndromes; therefore, the purpose of this review was to examine the prevalence of obstructive sleep apnea (OSA) in these populations. <ovid:br/>Method(s): All publications and poster presentations written in English found through August 2018 that describe the prevalence of sleep apnea among people with EDS or MFS were included. <ovid:br/>Result(s): A total of 13 studies were identifed, 7 for EDS and 6 for MFS. A combined random prevalence rate of OSA across both populations was 48.9% (95% confdence interval 38.3 59.6), with a slightly higher rate of 59.7% (39.7 77.0) for MFS versus 39.4% (28.8 51.1) for EDS. However, a high degree of heterogeneity across studies was found in both groups (EDS group: Q = 28.6 and I 2 = 79.0; MFS group: Q = 37.1 and I 2 = 86.5). When directly compared to the general population, patients with EDS/MFS were on average six times more likely (odds ratio 6.28 [95% confdence interval 3.31 11.93], P < 0.001, Z = 5.61) to have a diagnosis of OSA. <ovid:br/>Conclusion(s): OSA is a previously underestimated EDS/MFS-related complication. The high prevalence of OSA might be the result of bony and soft-tissue abnormalities associated with these hypermobility syndromes. Untreated OSA is thought to worsen cardiovascular complications especially among those with MFS. Further research is needed to better delineate whether the prevalence of OSA is moderated by factors such as sex, body mass index, bony structure, and disorder subtype.<ovid:br/>Copyright &#xa9; 2019 American Academy of Sleep Medicine. All Rights Reserved.

http://jcsm.aasm.org/ViewAbstract.aspx?pid=31511

http://ovidsp.ovid.com/ovidweb.cgi?T=JS&PAGE=reference&D=emed20&NEWS=N&AN=2001753055

https://ovidsp.ovid.com/ovidweb.cgi?T=JS&CSC=Y&NEWS=N&PAGE=fulltext&D=emed20&DO=10.5664%2fjcsm.7636

http://sfxhosted.exlibrisgroup.com/mayo?sid=OVID:embase&id=pmid:30736885&id=doi:10.5664%2Fjcsm.7636&issn=1550-9389&isbn=&volume=15&issue=2&spage=293&pages=293-299&date=2019&title=Journal+of+Clinical+Sleep+Medicine&atitle=Prevalence+of+obstructive+sleep+apnea+in+joint+hypermobility+syndrome%3A+A+systematic+review+and+meta-analysis&aulast=Sedky&pid=%3Cauthor%3ESedky+K.%3C%2Fauthor%3E&%3CAN%3E2001753055%3C%2FAN%3E

**65. Symptomatic Joint Hypermobility: The Hypermobile Type of Ehlers-Danlos Syndrome and the Hypermobility Spectrum Disorders**

Tinkle, B. T. and H. P. Levy (2019). "Symptomatic Joint Hypermobility: The Hypermobile Type of Ehlers-Danlos Syndrome and the Hypermobility Spectrum Disorders." Medical Clinics of North America 103(6): 1021

EP - 1033.

Joint hypermobility may be syndromic or nonsyndromic, asymptomatic or symptomatic. However, asymptomatic joint hypermobility can cause repetitive use injury, alter biomechanics, or become symptomatic later in life. Symptomatic joint hypermobility can result from soft tissue injury or muscular strain caused by muscular imbalance. Treatment is straightforward once joint hypermobility is recognized. Generalized joint hypermobility can be assessed using a standardized in-office examination. Generalized joint hypermobility may also be a feature of a heritable connective tissue disorder with other systemic findings. Therefore, assessing joint hypermobility in the context of musculoskeletal complaints may lead to recognizing systemic manifestations and allow treatment accordingly.<ovid:br/>Copyright &#xa9; 2019

http://www.medical.theclinics.com/

http://ovidsp.ovid.com/ovidweb.cgi?T=JS&PAGE=reference&D=emed20&NEWS=N&AN=2003122059

https://ovidsp.ovid.com/ovidweb.cgi?T=JS&CSC=Y&NEWS=N&PAGE=fulltext&D=emed20&DO=10.1016%2fj.mcna.2019.08.002

http://sfxhosted.exlibrisgroup.com/mayo?sid=OVID:embase&id=pmid:31582002&id=doi:10.1016%2Fj.mcna.2019.08.002&issn=0025-7125&isbn=&volume=103&issue=6&spage=1021&pages=1021-1033&date=2019&title=Medical+Clinics+of+North+America&atitle=Symptomatic+Joint+Hypermobility%3A+The+Hypermobile+Type+of+Ehlers-Danlos+Syndrome+and+the+Hypermobility+Spectrum+Disorders&aulast=Tinkle&pid=%3Cauthor%3ETinkle+B.T.%3C%2Fauthor%3E&%3CAN%3E2003122059%3C%2FAN%3E

**66. Ehlers-Danlos syndrome and other heritable connective tissue disorders that impact pregnancies can be detected using next-generation DNA sequencing**

VanderJagt, K. and M. G. Butler (2019). "Ehlers-Danlos syndrome and other heritable connective tissue disorders that impact pregnancies can be detected using next-generation DNA sequencing." Archives of Gynecology and Obstetrics 300(3): 491

EP - 493.

Ehlers-Danlos syndromes (EDS) are a genetically heterogeneous group of inherited connective tissue disorders classified into six major types with a variable collection of findings and different inheritance patterns. Although complications occur in about one-half of pregnancies in women with EDS, the majority can have a good outcome if managed appropriately. Classic EDS is characterized by joint hypermobility, loose skin with poor healing and easy bruising, musculoskeletal problems with chronic pain and at risk for pre-term delivery. In addition, the vascular form of EDS can have cardiac anomalies, aneurysms, gastrointestinal perforation and uterine rupture during pregnancy. Due to overlapping features among the connective tissue disorders, it is difficult to categorize the disorder into specific types without detailed genetic testing which is now available through advanced genomic technology using next-generation DNA sequencing, searching genomic databases and bioinformatics approach. Therefore, obstetrical complications are variable but relate to specific connective tissue disorders requiring an exact diagnosis. There are several dozen genes causing connective tissue disorders that are currently available for testing using next-generation sequencing and bioinformatics to provide pertinent care, treatment and surveillance of the affected pregnant woman but also for her at-risk fetus related to the specific heritable condition.<ovid:br/>Copyright &#xa9; 2019, Springer-Verlag GmbH Germany, part of Springer Nature.

https://www.springer.com/medicine/gynecology/journal/404

http://ovidsp.ovid.com/ovidweb.cgi?T=JS&PAGE=reference&D=emed20&NEWS=N&AN=2002529127

https://ovidsp.ovid.com/ovidweb.cgi?T=JS&CSC=Y&NEWS=N&PAGE=fulltext&D=emed20&DO=10.1007%2fs00404-019-05226-5

http://sfxhosted.exlibrisgroup.com/mayo?sid=OVID:embase&id=pmid:31250196&id=doi:10.1007%2Fs00404-019-05226-5&issn=0932-0067&isbn=&volume=300&issue=3&spage=491&pages=491-493&date=2019&title=Archives+of+Gynecology+and+Obstetrics&atitle=Ehlers-Danlos+syndrome+and+other+heritable+connective+tissue+disorders+that+impact+pregnancies+can+be+detected+using+next-generation+DNA+sequencing&aulast=VanderJagt&pid=%3Cauthor%3EVanderJagt+K.%3C%2Fauthor%3E&%3CAN%3E2002529127%3C%2FAN%3E

**67. Application of immunotherapy for neurological manifestations in hypermobile Ehlers-Danlos syndrome**

Araki, M., et al. (2018). "Application of immunotherapy for neurological manifestations in hypermobile Ehlers-Danlos syndrome." Therapeutic Advances in Neurological Disorders 11: 1756286418793766.

Ehlers-Danlos syndrome (EDS) is a heterogeneous heritable connective tissue disorder with various neurological manifestations, including chronic pain. The neurological manifestations in EDS are often regarded as being caused by the associated musculoskeletal disorders or polyneuropathy. Here, we present two patients with hypermobile EDS (hEDS), presenting with relapsing central nervous system (CNS) manifestations. Although the two patients showed relapsing signs of CNS manifestations like multiple sclerosis (MS) or neuromyelitis optica spectrum disorder (NMOSD), they were unique in that they had widespread opioid-dependent chronic pain, which is not consistent with the symptoms of MS/NMOSD. Unexpectedly, the serious pain of unknown origin was remarkably mitigated by plasmapheresis, and magnetic resonance imaging (MRI) examinations conducted for one of the patients were negative. Collectively, we speculate that hEDS may be more susceptible to 'normal-appearing imaging, neuroimmunologically justified, autoimmune-mediated encephalomyelitis (NINJA).' Analysis of the presented cases and an additional three patients with EDS with chronic pain indicates that treatable immune-mediated mechanisms deserve considerations for neurological symptoms observed in hEDS.<ovid:br/>Copyright &#xa9; The Author(s), 2018.

http://tan.sagepub.com/

http://ovidsp.ovid.com/ovidweb.cgi?T=JS&PAGE=reference&D=emed19&NEWS=N&AN=624227877

https://ovidsp.ovid.com/ovidweb.cgi?T=JS&CSC=Y&NEWS=N&PAGE=fulltext&D=emed19&DO=10.1177%2f1756286418793766

http://sfxhosted.exlibrisgroup.com/mayo?sid=OVID:embase&id=pmid:&id=doi:10.1177%2F1756286418793766&issn=1756-2856&isbn=&volume=11&issue=&spage=&pages=&date=2018&title=Therapeutic+Advances+in+Neurological+Disorders&atitle=Application+of+immunotherapy+for+neurological+manifestations+in+hypermobile+Ehlers-Danlos+syndrome&aulast=Araki&pid=%3Cauthor%3EAraki+M.%3C%2Fauthor%3E&%3CAN%3E624227877%3C%2FAN%3E

**68. Mitral valve prolapse and aortic root dilation in adults with hypermobile Ehlers-Danlos syndrome and related disorders**

Asher, S. B., et al. (2018). "Mitral valve prolapse and aortic root dilation in adults with hypermobile Ehlers-Danlos syndrome and related disorders." American Journal of Medical Genetics, Part A 176(9): 1838

EP - 1844.

Ehlers-Danlos syndromes (EDSs) are a group of inherited connective tissue disorders, and among them, classical EDS (cEDS) and hypermobile EDS (hEDS) are the most common. Mitral valve prolapse (MVP) and aortic root dilation (ARD) have previously been reported to occur at an increased frequency within cEDS and hEDS. More recently, a study performed in the pediatric population did not show increased prevalence (Ritter et al., American Journal of Medical Genetics Part A, 173(6), 1467-1472, 2017). The purpose of this study was to review a large population of individuals with cEDS, hEDS, and hypermobility spectrum disorders to determine the frequency of MVP and ARD. A retrospective chart review of 209 individuals with echocardiograms was performed. Overall, 6.4% (13/209) had MVP and 1.6% (3/189) were found to have ARD. Although the presence of MVP is higher than what has been reported in the general population, no patients had severe MVP or required surgical intervention. No patients in this cohort had an aortic root diameter requiring surgical repair. Based on the results of this study and previous studies, routine echocardiograms to assess for valvular diseases and ARD may not be necessary unless warranted by presence of symptoms or family history.<ovid:br/>Copyright &#xa9; 2018 Wiley Periodicals, Inc.

http://onlinelibrary.wiley.com/journal/10.1002/(ISSN)1552-4833

http://ovidsp.ovid.com/ovidweb.cgi?T=JS&PAGE=reference&D=emed19&NEWS=N&AN=623392138

https://ovidsp.ovid.com/ovidweb.cgi?T=JS&CSC=Y&NEWS=N&PAGE=fulltext&D=emed19&DO=10.1002%2fajmg.a.40364

http://sfxhosted.exlibrisgroup.com/mayo?sid=OVID:embase&id=pmid:30063091&id=doi:10.1002%2Fajmg.a.40364&issn=1552-4825&isbn=&volume=176&issue=9&spage=1838&pages=1838-1844&date=2018&title=American+Journal+of+Medical+Genetics%2C+Part+A&atitle=Mitral+valve+prolapse+and+aortic+root+dilation+in+adults+with+hypermobile+Ehlers-Danlos+syndrome+and+related+disorders&aulast=Asher&pid=%3Cauthor%3EAsher+S.B.%3C%2Fauthor%3E&%3CAN%3E623392138%3C%2FAN%3E

**69. Pharmacological resources, diagnostic approach and coordination of care in joint hypermobility-related disorders**

Baban, A. and M. Castori (2018). "Pharmacological resources, diagnostic approach and coordination of care in joint hypermobility-related disorders." Expert Review of Clinical Pharmacology 11(7): 689

EP - 703.

Introduction: Joint hypermobility (JH) is the hallmark of many hereditary soft connective tissue disorders, including Ehlers-Danlos syndromes and related disorders, disorders of the TGFbeta-pathway, lateral meningocele syndrome, arterial tortuosity syndrome, and cutis laxa syndromes. Contemporary practice separates individuals with isolated, non-syndromic JH from patients with Mendelian syndromes and those with hypermobility spectrum disorders. The latter is a new nosologic entity grouping together individuals with JH and related musculoskeletal manifestations, but lacking inclusion criteria for well-defined and/or single-gene disorders. Area covered: Nomenclature of JH and JH-related disorders are summarized on a practically oriented perspective. Critical areas of clinical management comprise pain; cardiovascular and respiratory issues; fatigue and dysautonomia; bone fragility; and capillary, skin and soft tissue fragility. Medical management stands on low-evidence data. Ongoing preclinical and clinical studies are aimed to reach a more personalized pharmacological approach to the management of the cardiovascular risk, musculoskeletal pain, and reduced bone mass. Expert commentary: Correct classification of patients with JH-related disorders needs a systematic approach, in which a wide array of molecular tests should be intermingled with strong clinical competences in highly specialized settings. A multispecialty, hierarchical approach should be encouraged for optimal coordination of care in systemic phenotypes.<ovid:br/>Copyright &#xa9; 2018, &#xa9; 2018 Informa UK Limited, trading as Taylor & Francis Group.

http://ovidsp.ovid.com/ovidweb.cgi?T=JS&PAGE=reference&D=emed19&NEWS=N&AN=623495170

https://ovidsp.ovid.com/ovidweb.cgi?T=JS&CSC=Y&NEWS=N&PAGE=fulltext&D=emed19&DO=10.1080%2f17512433.2018.1497973

http://sfxhosted.exlibrisgroup.com/mayo?sid=OVID:embase&id=pmid:29979900&id=doi:10.1080%2F17512433.2018.1497973&issn=1751-2433&isbn=&volume=11&issue=7&spage=689&pages=689-703&date=2018&title=Expert+Review+of+Clinical+Pharmacology&atitle=Pharmacological+resources%2C+diagnostic+approach+and+coordination+of+care+in+joint+hypermobility-related+disorders&aulast=Baban&pid=%3Cauthor%3EBaban+A.%3C%2Fauthor%3E&%3CAN%3E623495170%3C%2FAN%3E

**70. Hypermobility in children**

Coles, W., et al. (2018). "Hypermobility in children." Paediatrics and Child Health (United Kingdom) 28(2): 50

EP - 56.

Hypermobility can be a normal variant in children but it is also found in a group of children presenting with musculoskeletal pain. A smaller group of children will have hypermobility associated with a more concerning syndrome. The clinical challenge is to differentiate those children who have isolated hypermobility with no other associations from those with syndromes that have potentially life limiting complications. In this review we will discuss the assessment of and provide a framework for classifying those children who are found to be hypermobile. We review the components of connective tissue and describe where known the genetic basis for particular phenotypes. In 2017 the international classification of Ehlers Danlos (EDS) was published which supersedes the Villefranche classification. This review describes the diagnostic criteria for the common subtypes of EDS, provides information on further differentials and discusses the management options.<ovid:br/>Copyright &#xa9; 2017 Elsevier Ltd

https://www.elsevier-international.com

http://ovidsp.ovid.com/ovidweb.cgi?T=JS&PAGE=reference&D=emed19&NEWS=N&AN=620023801

https://ovidsp.ovid.com/ovidweb.cgi?T=JS&CSC=Y&NEWS=N&PAGE=fulltext&D=emed19&DO=10.1016%2fj.paed.2017.12.001

http://sfxhosted.exlibrisgroup.com/mayo?sid=OVID:embase&id=pmid:&id=doi:10.1016%2Fj.paed.2017.12.001&issn=1751-7222&isbn=&volume=28&issue=2&spage=50&pages=50-56&date=2018&title=Paediatrics+and+Child+Health+%28United+Kingdom%29&atitle=Hypermobility+in+children&aulast=Coles&pid=%3Cauthor%3EColes+W.%3C%2Fauthor%3E&%3CAN%3E620023801%3C%2FAN%3E

**71. Clinical features of children, adolescents, and adults with coexisting hypermobility syndromes and von Willebrand disease**

Hall, S. A., et al. (2018). "Clinical features of children, adolescents, and adults with coexisting hypermobility syndromes and von Willebrand disease." Pediatric Blood and Cancer 65(12): e27370.

We present five patients with coexistent von Willebrand disease (VWD) and Ehlers-Danlos syndrome and 21 with VWD and joint hypermobility. Females outnumbered males ten to three, Beighton scores were documented in 58% (15 of 26 patients), and several patients experienced moderately severe bleeding. We believe coexistent hypermobility disorder with VWD potentially affects bleeding severity and want to raise awareness among hematologists. Evaluation by geneticists is recommended because of the varying complexities observed across the disease spectrum, and the availability of new genetic tests should lead to more accurate diagnoses for the various hypermobility disorders.<ovid:br/>Copyright &#xa9; 2018 Wiley Periodicals, Inc.

http://onlinelibrary.wiley.com/journal/10.1002/(ISSN)1545-5017

http://ovidsp.ovid.com/ovidweb.cgi?T=JS&PAGE=reference&D=emed19&NEWS=N&AN=623775203

https://ovidsp.ovid.com/ovidweb.cgi?T=JS&CSC=Y&NEWS=N&PAGE=fulltext&D=emed19&DO=10.1002%2fpbc.27370

http://sfxhosted.exlibrisgroup.com/mayo?sid=OVID:embase&id=pmid:30084207&id=doi:10.1002%2Fpbc.27370&issn=1545-5009&isbn=&volume=65&issue=12&spage=e27370&pages=&date=2018&title=Pediatric+Blood+and+Cancer&atitle=Clinical+features+of+children%2C+adolescents%2C+and+adults+with+coexisting+hypermobility+syndromes+and+von+Willebrand+disease&aulast=Hall&pid=%3Cauthor%3EHall+S.A.%3C%2Fauthor%3E&%3CAN%3E623775203%3C%2FAN%3E

**72. Ehlers-Danlos Syndrome (EDS). From diagnosis to treatments. Third international symposium. Paris**

Hamonet, C. (2018). "Ehlers-Danlos Syndrome (EDS). From diagnosis to treatments. Third international symposium. Paris." Douleurs 19(1): 54

EP - 56.

A third international symposium on the syndrome of Ehlers-Danlos from diagnosis to treatments was held at the Faculty of Medicine of the University Paris-Descartes on 24 and 25 March 2017. He welcomed speakers from 10 different countries with the participation of Lara Bloom (New York), Executive Director of the International Ehlers-Danlos Society. The new classification of geneticists was presented and criticized. The new clinical criteria with mathematical modeling were presented. The following aspects have been developed: skin biopsy, gynecology, proprioception, pains, garments and orthotics, oxygentherapy, sleep, syndrome of mastocyts activation, psychopathology, autism, cognitive, disorders surgery, physical, and psychocomportementale rehabilitation.<ovid:br/>Copyright &#xa9; 2018

https://www.elsevier.com

http://ovidsp.ovid.com/ovidweb.cgi?T=JS&PAGE=reference&D=emed19&NEWS=N&AN=620767413

https://ovidsp.ovid.com/ovidweb.cgi?T=JS&CSC=Y&NEWS=N&PAGE=fulltext&D=emed19&DO=10.1016%2fj.douler.2017.12.001

http://sfxhosted.exlibrisgroup.com/mayo?sid=OVID:embase&id=pmid:&id=doi:10.1016%2Fj.douler.2017.12.001&issn=1624-5687&isbn=&volume=19&issue=1&spage=54&pages=54-56&date=2018&title=Douleurs&atitle=Syndrome+d%27Ehlers-Danlos+%28SED%29.+Du+diagnostic+aux+traitements.+Troisieme+colloque+international.+Paris&aulast=Hamonet&pid=%3Cauthor%3EHamonet+C.%3C%2Fauthor%3E&%3CAN%3E620767413%3C%2FAN%3E

**73. Characteristics, Diagnosis, and Management of Ehlers-Danlos Syndromes: A Review**

Joseph, A. W., et al. (2018). "Characteristics, Diagnosis, and Management of Ehlers-Danlos Syndromes: A Review." JAMA facial plastic surgery 20(1): 70

EP - 75.

Importance: Ehlers-Danlos syndromes (EDSs) are a group of heritable connective tissue disorders. Patients with EDSs can develop excessive facial rhytids, nasal deformities, and facial scarring, for which they may seek consultation with a facial plastic surgeon. Ehlers-Danlos syndromes can be associated with serious surgical complications and should be identified preoperatively to facilitate optimal treatment. To our knowledge, no management guidelines for patients with EDSs exist in the facial plastic surgery literature. We present a review of the literature and management recommendations for the facial plastic surgeon. Observations: We performed a PubMed/MEDLINE search for all publications in the English language related to surgical experience in patients with EDSs. A total of 37 publications (including reviews and case series) were included. Ehlers-Danlos syndromes are more common than appreciated, with an overall point prevalence between 1 in 2500 and 1 in 5000 according to existing literature. There are 6 main subtypes of EDSs that have significant phenotypic heterogeneity. Patients with classic type and hypermobile EDS (the 2 most common subtypes) can typically undergo elective facial plastic surgery without major complications. However, specific preoperative, intraoperative, and postoperative issues, as outlined in this review, must be taken into consideration. Vascular EDS should serve as a contraindication to elective surgery given its potential for life-threatening complications. <ovid:br/>Conclusions and Relevance: Because of their skin laxity, patients with EDSs may present more frequently to facial plastic surgeons' offices. Simple screening questions can help the surgeon identify potential patients with EDSs. Treatment should be individualized and performed with extreme care to ensure optimal outcomes.

http://ovidsp.ovid.com/ovidweb.cgi?T=JS&PAGE=reference&D=emed19&NEWS=N&AN=629201895

https://ovidsp.ovid.com/ovidweb.cgi?T=JS&CSC=Y&NEWS=N&PAGE=fulltext&D=emed19&DO=10.1001%2fjamafacial.2017.0793

http://sfxhosted.exlibrisgroup.com/mayo?sid=OVID:embase&id=pmid:29121166&id=doi:10.1001%2Fjamafacial.2017.0793&issn=2168-6092&isbn=&volume=20&issue=1&spage=70&pages=70-75&date=2018&title=JAMA+facial+plastic+surgery&atitle=Characteristics%2C+Diagnosis%2C+and+Management+of+Ehlers-Danlos+Syndromes%3A+A+Review&aulast=Joseph&pid=%3Cauthor%3EJoseph+A.W.%3C%2Fauthor%3E&%3CAN%3E629201895%3C%2FAN%3E

**74. Physical and mechanical therapies for lower limb symptoms in children with Hypermobility Spectrum Disorder and Hypermobile Ehlers-Danlos Syndrome: a systematic review**

Peterson, B., et al. (2018). "Physical and mechanical therapies for lower limb symptoms in children with Hypermobility Spectrum Disorder and Hypermobile Ehlers-Danlos Syndrome: a systematic review." Journal of foot and ankle research 11: 59.

Background: Hypermobility Spectrum Disorder and Hypermobile Ehlers Danlos Syndrome are two common heritable genetic disorders of connective tissue. Both conditions are characterised by excessive joint range of motion and the presence of musculoskeletal symptoms, and are associated with joint instability, motion incoordination, decreased joint position sense, and musculoskeletal pain. Hypermobility Spectrum Disorder is the new classification for what was previously known as Joint Hypermobility Syndrome. This systematic review evaluates the evidence for physical and mechanical treatments for lower limb problems in children with Hypermobility Spectrum Disorder and Hypermobile Ehlers Danlos Syndrome. <ovid:br/>Method(s): MEDLINE, EMBASE, Cochrane Central Register of Controlled Trials, PUBMED and CINAHL were searched to October 2017 for randomised controlled trials (RCT) and quasi-RCTs evaluating physical and mechanical interventions for lower limb problems in children with hypermobility. Two authors independently screened studies for eligibility for inclusion and three review authors independently assessed risk of bias of included studies. One author extracted and analysed statistical data, which were checked by a second author. <ovid:br/>Result(s): Two RCTs including a total of 86 participants were eligible for inclusion. Trials evaluated differences between generalised versus targeted physiotherapy programs and between performing knee extension exercises to the neutral versus hypermobile range. There was no clear benefit of any of the physical therapies evaluated. <ovid:br/>Conclusion(s): There is very limited evidence to guide the use of physical and mechanical therapies for lower limb problems in children with Hypermobility Spectrum Disorder and Hypermobile Ehlers Danlos Syndrome. Mechanical therapies have not been evaluated in RCTs and results of the two RCTs of physical therapies do not definitively guide physical therapy prescriptions. Current studies are limited by small sample sizes and high attrition rates. No physical therapy has been compared to a sham intervention no intervention or no intervention, so overall effectiveness is unknown.

http://ovidsp.ovid.com/ovidweb.cgi?T=JS&PAGE=reference&D=emed19&NEWS=N&AN=625057715

https://ovidsp.ovid.com/ovidweb.cgi?T=JS&CSC=Y&NEWS=N&PAGE=fulltext&D=emed19&DO=10.1186%2fs13047-018-0302-1

http://sfxhosted.exlibrisgroup.com/mayo?sid=OVID:embase&id=pmid:30455744&id=doi:10.1186%2Fs13047-018-0302-1&issn=1757-1146&isbn=&volume=11&issue=&spage=59&pages=59&date=2018&title=Journal+of+foot+and+ankle+research&atitle=Physical+and+mechanical+therapies+for+lower+limb+symptoms+in+children+with+Hypermobility+Spectrum+Disorder+and+Hypermobile+Ehlers-Danlos+Syndrome%3A+a+systematic+review&aulast=Peterson&pid=%3Cauthor%3EPeterson+B.%3C%2Fauthor%3E&%3CAN%3E625057715%3C%2FAN%3E

**75. Postural tachycardia syndrome and other forms of orthostatic intolerance in Ehlers-Danlos syndrome**

Roma, M., et al. (2018). "Postural tachycardia syndrome and other forms of orthostatic intolerance in Ehlers-Danlos syndrome." Autonomic Neuroscience: Basic and Clinical 215: 89

EP - 96.

Objective: To review the association between orthostatic intolerance syndromes and both joint hypermobility and Ehlers-Danlos syndrome, and to propose reasons for identifying hereditary connective tissue disorders in those with orthostatic intolerance in the context of both clinical care and research. <ovid:br/>Method(s): We searched the published peer-reviewed medical literature for papers reporting an association between joint hypermobility or Ehlers-Danlos syndrome and orthostatic intolerance. <ovid:br/>Result(s): We identified 10 relevant papers. Although methodological variability between studies introduces some limitations, the published literature consistently identifies a significantly higher prevalence of orthostatic intolerance symptoms in patients with joint hypermobility or Ehlers-Danlos syndrome than in healthy controls, and a significantly higher prevalence of cardiovascular and autonomic abnormalities both at rest and during orthostatic challenge. Postural tachycardia syndrome is the most commonly recognized circulatory disorder. The severity of orthostatic symptoms in those with EDS correlates with impairments in quality of life. <ovid:br/>Conclusion(s): There is a strong association between several forms of cardiovascular dysfunction, most notably postural tachycardia syndrome, and joint hypermobility or Ehlers-Danlos syndrome. We propose that recognition of joint hypermobility and Ehlers-Danlos syndrome among those with orthostatic intolerance syndromes has the potential to improve clinical care and the validity of research findings.<ovid:br/>Copyright &#xa9; 2018 Elsevier B.V.

https://www.elsevier.com/locate/jans

http://ovidsp.ovid.com/ovidweb.cgi?T=JS&PAGE=reference&D=emed19&NEWS=N&AN=2000508350

https://ovidsp.ovid.com/ovidweb.cgi?T=JS&CSC=Y&NEWS=N&PAGE=fulltext&D=emed19&DO=10.1016%2fj.autneu.2018.02.006

http://sfxhosted.exlibrisgroup.com/mayo?sid=OVID:embase&id=pmid:29519641&id=doi:10.1016%2Fj.autneu.2018.02.006&issn=1566-0702&isbn=&volume=215&issue=&spage=89&pages=89-96&date=2018&title=Autonomic+Neuroscience%3A+Basic+and+Clinical&atitle=Postural+tachycardia+syndrome+and+other+forms+of+orthostatic+intolerance+in+Ehlers-Danlos+syndrome&aulast=Roma&pid=%3Cauthor%3ERoma+M.%3C%2Fauthor%3E&%3CAN%3E2000508350%3C%2FAN%3E

**76. Ehlers-Danlos syndromes**

Germain, D. P. (2017). "Ehlers-Danlos syndromes." Annales de Dermatologie et de Venereologie 144(12): 744

EP - 758.

Ehlers-Danlos syndromes (EDS) are a heterogeneous group of inheritable connective tissue disorders characterized by skin hyperextensibility, joint hypermobility and cutaneous fragility with delayed wound healing. Over and above these common features, they differ in the presence or absence of various organ and tissue abnormalities, and differences in genetic causal mechanisms and degree of severity. They are complex and multisystem diseases, with the majority being highly disabling because of major joint problems and neurosensory deficiencies, and in some cases, they may be life-threatening due to associated complications, especially vascular disorders. In 1997, the Villefranche classification defined 6 subtypes of EDS. However, many other new variants have been described over the last years. The "historical" EDS were characterized by abnormalities in fibrillar collagen protein synthesis. More recently, disorders of synthesis and organization of the extracellular matrix have been shown to be responsible for other types of EDS. Thus, many EDS are in fact metabolic diseases related to enzymatic defects. While there is no curative treatment for any type of EDS, early diagnosis is of utmost importance in order to optimize the symptomatic management of patients and to prevent avoidable complications. Patients must be treated and monitored by multidisciplinary teams in highly specialized reference centers. In this article, we present the current state of knowledge on these diseases that continue to be elucidated thanks to new molecular genetic techniques.<ovid:br/>Copyright &#xa9; 2017 Elsevier Masson SAS

https://www.elsevier.com/journals/annales-de-dermatologie-et-de-venereologie/0151-9638

http://ovidsp.ovid.com/ovidweb.cgi?T=JS&PAGE=reference&D=emed18&NEWS=N&AN=618675133

https://ovidsp.ovid.com/ovidweb.cgi?T=JS&CSC=Y&NEWS=N&PAGE=fulltext&D=emed18&DO=10.1016%2fj.annder.2017.06.017

http://sfxhosted.exlibrisgroup.com/mayo?sid=OVID:embase&id=pmid:29032848&id=doi:10.1016%2Fj.annder.2017.06.017&issn=0151-9638&isbn=&volume=144&issue=12&spage=744&pages=744-758&date=2017&title=Annales+de+Dermatologie+et+de+Venereologie&atitle=Les+syndromes+d%27Ehlers-Danlos&aulast=Germain&pid=%3Cauthor%3EGermain+D.-P.%3C%2Fauthor%3E&%3CAN%3E618675133%3C%2FAN%3E

**77. Generalized Hyperalgesia in Children and Adults Diagnosed With Hypermobility Syndrome and Ehlers-Danlos Syndrome Hypermobility Type: A Discriminative Analysis**

Scheper, M. C., et al. (2017). "Generalized Hyperalgesia in Children and Adults Diagnosed With Hypermobility Syndrome and Ehlers-Danlos Syndrome Hypermobility Type: A Discriminative Analysis." Arthritis Care and Research 69(3): 421

EP - 429.

Objective: Lowered pressure-pain thresholds have been demonstrated in adults with Ehlers-Danlos syndrome hypermobility type (EDS-HT), but whether these findings are also present in children is unclear. Therefore, the objectives of the study were to determine whether generalized hyperalgesia is present in children with hypermobility syndrome (HMS)/EDS-HT, explore potential differences in pressure-pain thresholds between children and adults with HMS/EDS-HT, and determine the discriminative value of generalized hyperalgesia. <ovid:br/>Method(s): Patients were classified in 1 of 3 groups: HMS/EDS-HT, hypermobile (Beighton score >=4 of 9), and healthy controls. Descriptive data of age, sex, body mass index, Beighton score, skin laxity, and medication usage were collected. Generalized hyperalgesia was quantified by the average pressure-pain thresholds collected from 12 locations. Confounders collected were pain locations/intensity, fatigue, and psychological distress. Comparisons between children with HMS/EDS-HT and normative values, between children and adults with HMS/EDS-HT, and corrected confounders were analyzed with multivariate analysis of covariance. The discriminative value of generalized hyperalgesia employed to differentiate between HMS/EDS-HT, hypermobility, and controls was quantified with logistic regression. <ovid:br/>Result(s): Significantly lower pressure-pain thresholds were found in children with HMS/EDS-HT compared to normative values (range -22.0% to -59.0%; P <= 0.05). When applying a threshold of 30.8 N/cm<ovid:sup>2</ovid:sup> for males and 29.0 N/cm<ovid:sup>2</ovid:sup> for females, the presence of generalized hyperalgesia discriminated between individuals with HMS/EDS-HT, hypermobility, and healthy controls (odds ratio 6.0). <ovid:br/>Conclusion(s): Children and adults with HMS/EDS-HT are characterized by hypermobility, chronic pain, and generalized hyperalgesia. The presence of generalized hyperalgesia may indicate involvement of the central nervous system in the development of chronic pain.<ovid:br/>Copyright &#xa9; 2016, American College of Rheumatology

http://onlinelibrary.wiley.com/journal/10.1002/(ISSN)2151-4658

http://ovidsp.ovid.com/ovidweb.cgi?T=JS&PAGE=reference&D=emed18&NEWS=N&AN=614598970

https://ovidsp.ovid.com/ovidweb.cgi?T=JS&CSC=Y&NEWS=N&PAGE=fulltext&D=emed18&DO=10.1002%2facr.22998

http://sfxhosted.exlibrisgroup.com/mayo?sid=OVID:embase&id=pmid:27483212&id=doi:10.1002%2Facr.22998&issn=2151-464X&isbn=&volume=69&issue=3&spage=421&pages=421-429&date=2017&title=Arthritis+Care+and+Research&atitle=Generalized+Hyperalgesia+in+Children+and+Adults+Diagnosed+With+Hypermobility+Syndrome+and+Ehlers-Danlos+Syndrome+Hypermobility+Type%3A+A+Discriminative+Analysis&aulast=Scheper&pid=%3Cauthor%3EScheper+M.C.%3C%2Fauthor%3E&%3CAN%3E614598970%3C%2FAN%3E

**78. Difficulty eating and significant weight loss in joint hypermobility syndrome/Ehlers-Danlos syndrome, hypermobility type**

Baeza-Velasco, C., et al. (2016). "Difficulty eating and significant weight loss in joint hypermobility syndrome/Ehlers-Danlos syndrome, hypermobility type." Eating and Weight Disorders 21(2): 175

EP - 183.

Joint Hypermobility Syndrome, also known as Ehlers-Danlos Syndrome Hypermobility Type (JHS/EDS-HT), is a heritable disorder of connective tissue, common but poorly known by the medical community. Although generalized joint hypermobility and fragility of tissues have been described as core features, recent research highlights the multisystemic nature of JHS/EDS-HT, which presents with a wide range of articular and extra-articular symptoms. Among these, gastrointestinal problems, temporomandibular disorders, and smell and taste abnormalities are common among those affected, having significant implications for eating. The present work reviews the literature linking JHS/EDS-HT and eating problems. Two illustrative case reports, in which JHS/EDS-HT manifestations contribute to developing and maintaining disturbed eating behaviors and significant weight loss, are presented.<ovid:br/>Copyright &#xa9; 2015, Springer International Publishing Switzerland.

http://link.springer.com/journal/40519

http://ovidsp.ovid.com/ovidweb.cgi?T=JS&PAGE=reference&D=emed17&NEWS=N&AN=610503485

https://ovidsp.ovid.com/ovidweb.cgi?T=JS&CSC=Y&NEWS=N&PAGE=fulltext&D=emed17&DO=10.1007%2fs40519-015-0232-x

http://sfxhosted.exlibrisgroup.com/mayo?sid=OVID:embase&id=pmid:26506923&id=doi:10.1007%2Fs40519-015-0232-x&issn=1124-4909&isbn=&volume=21&issue=2&spage=175&pages=175-183&date=2016&title=Eating+and+Weight+Disorders&atitle=Difficulty+eating+and+significant+weight+loss+in+joint+hypermobility+syndrome%2FEhlers-Danlos+syndrome%2C+hypermobility+type&aulast=Baeza-Velasco&pid=%3Cauthor%3EBaeza-Velasco+C.%3C%2Fauthor%3E&%3CAN%3E610503485%3C%2FAN%3E

**79. Pain in Ehlers-Danlos syndromes: manifestations, therapeutic strategies and future perspectives**

Castori, M. (2016). "Pain in Ehlers-Danlos syndromes: manifestations, therapeutic strategies and future perspectives." Expert Opinion on Orphan Drugs 4(11): 1145

EP - 1158.

Introduction: Ehlers-Danlos syndrome (EDS) groups together an increasing number of hereditary soft connective tissue disorders. Among the most common variants, the hypermobility type emerges as the most problematic, due to clinical similarities with the joint hypermobility syndrome, strong association with pain and lack of molecular confirmatory tests. To date, chronic pain and the related physical disability are the most relevant clinical issues in the long-term management of EDS. Areas covered: A literature review was carried out covering all known pain manifestations, i.e. musculoskeletal pain, neuropathic pain, central sensitization, headache and visceral pain, in EDS. The natural history of pain, as well as other critical issues, i.e. heterogeneity, clinical approach and evolving phenotype, of EDS are also addressed. All available data on therapeutic strategies for pain in EDS are extensively reported. Expert opinion: Multidisciplinarity emerges as an undeniable prerequisite for the management of the complex EDS patient reporting pain. All available therapeutic resources, comprising painkillers, physical and psychological therapies, and surgery, show a low evidence of efficacy in the long-term treatment of pain. Education and early diagnosis are primary resources for secondary and tertiary prevention. Novel drug therapies could be developed considering the potential role of a dysfunctional extracellular matrix on pain modulation in EDS.<ovid:br/>Copyright &#xa9; 2016 Informa UK Limited, trading as Taylor & Francis Group.

http://ovidsp.ovid.com/ovidweb.cgi?T=JS&PAGE=reference&D=emed17&NEWS=N&AN=612910938

https://ovidsp.ovid.com/ovidweb.cgi?T=JS&CSC=Y&NEWS=N&PAGE=fulltext&D=emed17&DO=10.1080%2f21678707.2016.1238302

http://sfxhosted.exlibrisgroup.com/mayo?sid=OVID:embase&id=pmid:&id=doi:10.1080%2F21678707.2016.1238302&issn=2167-8707&isbn=&volume=4&issue=11&spage=1145&pages=1145-1158&date=2016&title=Expert+Opinion+on+Orphan+Drugs&atitle=Pain+in+Ehlers-Danlos+syndromes%3A+manifestations%2C+therapeutic+strategies+and+future+perspectives&aulast=Castori&pid=%3Cauthor%3ECastori+M.%3C%2Fauthor%3E&%3CAN%3E612910938%3C%2FAN%3E

**80. Epidemiology of bleeding symptoms and hypermobile Ehlers-Danlos syndrome in paediatrics**

Hickey, S. E., et al. (2016). "Epidemiology of bleeding symptoms and hypermobile Ehlers-Danlos syndrome in paediatrics." Haemophilia 22(5): e490

EP - e493.

http://onlinelibrary.wiley.com/journal/10.1111/(ISSN)1365-2516

http://ovidsp.ovid.com/ovidweb.cgi?T=JS&PAGE=reference&D=emed17&NEWS=N&AN=612628190

https://ovidsp.ovid.com/ovidweb.cgi?T=JS&CSC=Y&NEWS=N&PAGE=fulltext&D=emed17&DO=10.1111%2fhae.13063

http://sfxhosted.exlibrisgroup.com/mayo?sid=OVID:embase&id=pmid:27562077&id=doi:10.1111%2Fhae.13063&issn=1351-8216&isbn=&volume=22&issue=5&spage=e490&pages=e490-e493&date=2016&title=Haemophilia&atitle=Epidemiology+of+bleeding+symptoms+and+hypermobile+Ehlers-Danlos+syndrome+in+paediatrics&aulast=Hickey&pid=%3Cauthor%3EHickey+S.E.%3C%2Fauthor%3E&%3CAN%3E612628190%3C%2FAN%3E

**81. Ehlers danlos syndrome - A review**

Sathiyamoorthy, S. (2016). "Ehlers danlos syndrome - A review." Journal of Pharmaceutical Sciences and Research 8(8): 750

EP - 751.

The study aims in reviewing the causes, symptoms, prevalence and diagnosis of Ehlers Danlos syndrome. Ehlers- Danlos syndrome (EDS) is an inherited connective tissue disorder. It is caused by a defect in the structure, production, or processing of collagen or proteins that interact with collagen, such as mutations in the COL5A or COL3A genes. The extra oral manifestations of EDS are the presence of scarring on the chin and forehead, a history of repeated luxations of the TMJ, epicanthus, hypertelorism, a narrow curved nose, sparse hair and hyper-elasticity of the skin. As fragile as the skin, the mucosa tears easily when touched by instruments. Periodontal tissues lead to the premature loss of deciduous and permanent teeth.<ovid:br/>Copyright &#xa9; 2016, Pharmainfo Publications. All rights reserved.

http://www.jpsr.pharmainfo.in/Documents/Volumes/vol8Issue08/jpsr08081610.pdf

http://ovidsp.ovid.com/ovidweb.cgi?T=JS&PAGE=reference&D=emed17&NEWS=N&AN=612022058

https://ovidsp.ovid.com/ovidweb.cgi?T=JS&CSC=Y&NEWS=N&PAGE=fulltext&D=emed17&AN=612022058

http://sfxhosted.exlibrisgroup.com/mayo?sid=OVID:embase&id=pmid:&id=doi:&issn=0975-1459&isbn=&volume=8&issue=8&spage=750&pages=750-751&date=2016&title=Journal+of+Pharmaceutical+Sciences+and+Research&atitle=Ehlers+danlos+syndrome+-+A+review&aulast=Sathiyamoorthy&pid=%3Cauthor%3ESathiyamoorthy+S.%3C%2Fauthor%3E&%3CAN%3E612022058%3C%2FAN%3E

**82. Disability in Adolescents and Adults Diagnosed With Hypermobility-Related Disorders: A Meta-Analysis**

Scheper, M. C., et al. (2016). "Disability in Adolescents and Adults Diagnosed With Hypermobility-Related Disorders: A Meta-Analysis." Archives of Physical Medicine and Rehabilitation 97(12): 2174

EP - 2187.

Objective To (1) establish the association of the most common reported symptoms on disability; and (2) study the effectiveness of treatment on disability in patients with Ehlers-Danlos syndrome-hypermobility type (EDS-HT)/hypermobility syndrome (HMS). Data Sources An electronic search (Medical Subject Headings and free-text terms) was conducted in bibliographic databases CENTRAL/MEDLINE. Study Selection Comparative, cross-sectional, longitudinal cohort studies and (randomized) controlled trials including patients with HMS/EDS-HT aged >=17 years were considered for inclusion. A class of symptoms was included when 5 publications were available. In regards to treatment (physical, cognitive interventions), only (randomized) controlled trials were considered. Surgical and medicinal interventions were excluded. Data Extraction Bias was assessed according to the methodological scoring tools of the Cochrane collaboration. Z-score transformations were applied to classify the extent of disability in comparison with healthy controls and to ensure comparability between studies. Data Synthesis Initially, the electronic search yielded 714 publications, and 21 articles remained for analysis after selection. The following symptoms were included for meta-analysis: pain (n=12), fatigue (n=6), and psychological distress (n=7). Pain (r=.64, P=.021), fatigue (r=.91, P=.011), and psychological distress (r=.86, P=.018) had a significant impact on disability. Regarding treatment, a significant pain reduction was achieved by a variety of physical and cognitive approaches. Treatment effectiveness on disability was not established. Conclusions Disability can affect patients with HMS/EDS-HT significantly and is highly correlated with both physical and psychological factors. Although evidence is available that physical and psychological treatment modalities can induce significant pain reduction, the evidence regarding disability reduction is lacking.<ovid:br/>Copyright &#xa9; 2016 American Congress of Rehabilitation Medicine

http://www.elsevier.com/inca/publications/store/6/2/3/3/5/4/index.htt

http://ovidsp.ovid.com/ovidweb.cgi?T=JS&PAGE=reference&D=emed17&NEWS=N&AN=610446925

https://ovidsp.ovid.com/ovidweb.cgi?T=JS&CSC=Y&NEWS=N&PAGE=fulltext&D=emed17&DO=10.1016%2fj.apmr.2016.02.015

http://sfxhosted.exlibrisgroup.com/mayo?sid=OVID:embase&id=pmid:26976801&id=doi:10.1016%2Fj.apmr.2016.02.015&issn=0003-9993&isbn=&volume=97&issue=12&spage=2174&pages=2174-2187&date=2016&title=Archives+of+Physical+Medicine+and+Rehabilitation&atitle=Disability+in+Adolescents+and+Adults+Diagnosed+With+Hypermobility-Related+Disorders%3A+A+Meta-Analysis&aulast=Scheper&pid=%3Cauthor%3EScheper+M.C.%3C%2Fauthor%3E&%3CAN%3E610446925%3C%2FAN%3E

**83. Connective tissue, Ehlers-Danlos syndrome(s), and head and cervical pain**

Castori, M., et al. (2015). "Connective tissue, Ehlers-Danlos syndrome(s), and head and cervical pain." American Journal of Medical Genetics, Part C: Seminars in Medical Genetics 169(1): 84

EP - 96.

Ehlers-Danlos syndrome (EDS) is an umbrella term for a growing group of hereditary disorders of the connective tissue mainly manifesting with generalized joint hypermobility, skin hyperextensibility, and vascular and internal organ fragility. In contrast with other well known heritable connective tissue disorders with severe cardiovascular involvement (e.g., Marfan syndrome), most EDS patients share a nearly normal life span, but are severely limited by disabling features, such as pain, fatigue and headache. In this work, pertinent literature is reviewed with focus on prevalence, features and possible pathogenic mechanisms of headache in EDSs. Gathered data are fragmented and generally have a low level of evidence. Headache is reported in no less than 1/3 of the patients. Migraine results the most common type in the hypermobility type of EDS. Other possibly related headache disorders include tension-type headache, new daily persistent headache, headache attributed to spontaneous cerebrospinal fluid leakage, headache secondary to Chiari malformation, cervicogenic headache and neck-tongue syndrome, whose association still lacks of reliable prevalence studies. The underlying pathogenesis seems complex and variably associated with cardiovascular dysautonomia, cervical spine and temporomandibular joint instability/dysfunction, meningeal fragility, poor sleep quality, pain-killer drugs overuse and central sensitization. Particular attention is posed on a presumed subclinical cervical spine dysfunction. Standard treatment is always symptomatic and usually unsuccessful. Assessment and management procedures are discussed in order to put some basis for ameliorating the actual patients' needs and nurturing future research.<ovid:br/>Copyright &#xa9; 2015 Wiley Periodicals, Inc.

http://onlinelibrary.wiley.com/journal/10.1002/(ISSN)1552-4876

http://ovidsp.ovid.com/ovidweb.cgi?T=JS&PAGE=reference&D=emed16&NEWS=N&AN=602095018

https://ovidsp.ovid.com/ovidweb.cgi?T=JS&CSC=Y&NEWS=N&PAGE=fulltext&D=emed16&DO=10.1002%2fajmg.c.31426

http://sfxhosted.exlibrisgroup.com/mayo?sid=OVID:embase&id=pmid:25655119&id=doi:10.1002%2Fajmg.c.31426&issn=1552-4868&isbn=&volume=169&issue=1&spage=84&pages=84-96&date=2015&title=American+Journal+of+Medical+Genetics%2C+Part+C%3A+Seminars+in+Medical+Genetics&atitle=Connective+tissue%2C+Ehlers-Danlos+syndrome%28s%29%2C+and+head+and+cervical+pain&aulast=Castori&pid=%3Cauthor%3ECastori+M.%3C%2Fauthor%3E&%3CAN%3E602095018%3C%2FAN%3E

**84. Ehlers-Danlos syndrome in rheumatology: Diagnostic and therapeutic challenges**

Challal, S., et al. (2015). "Ehlers-Danlos syndrome in rheumatology: Diagnostic and therapeutic challenges." Joint Bone Spine 82(5): 305

EP - 307.

https://www.elsevier.com/html/detrevue.cfm?code=RR

http://ovidsp.ovid.com/ovidweb.cgi?T=JS&PAGE=reference&D=emed16&NEWS=N&AN=604478345

https://ovidsp.ovid.com/ovidweb.cgi?T=JS&CSC=Y&NEWS=N&PAGE=fulltext&D=emed16&DO=10.1016%2fj.jbspin.2015.04.002

http://sfxhosted.exlibrisgroup.com/mayo?sid=OVID:embase&id=pmid:26005130&id=doi:10.1016%2Fj.jbspin.2015.04.002&issn=1297-319X&isbn=&volume=82&issue=5&spage=305&pages=305-307&date=2015&title=Joint+Bone+Spine&atitle=Ehlers-Danlos+syndrome+in+rheumatology%3A+Diagnostic+and+therapeutic+challenges&aulast=Challal&pid=%3Cauthor%3EChallal+S.%3C%2Fauthor%3E&%3CAN%3E604478345%3C%2FAN%3E

**85. Differential diagnosis and diagnostic flow chart of joint hypermobility syndrome/ehlers-danlos syndrome hypermobility type compared to other heritable connective tissue disorders**

Colombi, M., et al. (2015). "Differential diagnosis and diagnostic flow chart of joint hypermobility syndrome/ehlers-danlos syndrome hypermobility type compared to other heritable connective tissue disorders." American Journal of Medical Genetics, Part C: Seminars in Medical Genetics 169(1): 6

EP - 22.

Joint hypermobility syndrome/Ehlers-Danlos syndrome hypermobility type (JHS/EDS-HT) is an evolving and protean disorder mostly recognized by generalized joint hypermobility and without a defined molecular basis. JHS/EDS-HT also presents with other connective tissue features affecting a variety of structures and organs, such as skin, eye, bone, and internal organs. However, most of these signs are present in variable combinations and severity in many other heritable connective tissue disorders. Accordingly, JHS/EDS-HT is an "exclusion" diagnosis which needs the absence of any consistent feature indicative of other partially overlapping connective tissue disorders. While both Villefranche and Brighton criteria include such an exclusion as a mandatory item, a systematic approach for reaching a stringent clinical diagnosis of JHS/EDS-HT is still lacking. The absence of a consensus on the diagnostic approach to JHS/EDS-HT concerning its clinical boundaries with similar conditions contribute to limit our actual understanding of the pathologic and molecular bases of this disorder. In this review, we revise the differential diagnosis of JHS/EDS-HT with those heritable connective tissue disorders which show a significant overlap with the former and mostly include EDS classic, vascular and kyphoscoliotic types, osteogenesis imperfecta, Marfan syndrome, Loeys-Dietz syndrome, arterial tortuosity syndrome, and lateral meningocele syndrome. A diagnostic flow chart is also offered with the attempt to support the less experienced clinician in stringently recognizing JHS/EDS-HT and stimulate the debate in the scientific community for both management and research purposes.<ovid:br/>Copyright &#xa9; 2015 Wiley Periodicals, Inc.

http://onlinelibrary.wiley.com/journal/10.1002/(ISSN)1552-4876

http://ovidsp.ovid.com/ovidweb.cgi?T=JS&PAGE=reference&D=emed16&NEWS=N&AN=603278953

https://ovidsp.ovid.com/ovidweb.cgi?T=JS&CSC=Y&NEWS=N&PAGE=fulltext&D=emed16&DO=10.1002%2fajmg.c.31429

http://sfxhosted.exlibrisgroup.com/mayo?sid=OVID:embase&id=pmid:25821090&id=doi:10.1002%2Fajmg.c.31429&issn=1552-4868&isbn=&volume=169&issue=1&spage=6&pages=6-22&date=2015&title=American+Journal+of+Medical+Genetics%2C+Part+C%3A+Seminars+in+Medical+Genetics&atitle=Differential+diagnosis+and+diagnostic+flow+chart+of+joint+hypermobility+syndrome%2Fehlers-danlos+syndrome+hypermobility+type+compared+to+other+heritable+connective+tissue+disorders&aulast=Colombi&pid=%3Cauthor%3EColombi+M.%3C%2Fauthor%3E&%3CAN%3E603278953%3C%2FAN%3E

**86. The neuromuscular differential diagnosis of joint hypermobility**

Donkervoort, S., et al. (2015). "The neuromuscular differential diagnosis of joint hypermobility." American Journal of Medical Genetics, Part C: Seminars in Medical Genetics 169(1): 23

EP - 42.

Joint hypermobility is the defining feature of various inherited connective tissue disorders such as Marfan syndrome and various types of Ehlers-Danlos syndrome and these will generally be the first conditions to be considered by geneticists and pediatricians in the differential diagnosis of a patient presenting with such findings. However, several congenital and adult-onset inherited myopathies also present with joint hypermobility in the context of often only mild-to-moderate muscle weakness and should, therefore, be included in the differential diagnosis of joint hypermobility. In fact, on the molecular level disorders within both groups represent different ends of the same spectrum of inherited extracellular matrix (ECM) disorders. In this review we will summarize the measures of joint hypermobility, illustrate molecular mechanisms these groups of disorders have in common, and subsequently discuss the clinical features of: 1) the most common connective tissue disorders with myopathic or other neuromuscular features: Ehlers-Danlos syndrome, Marfan syndrome and Loeys-Dietz syndrome; 2) myopathy and connective tissue overlap disorders (muscle extracellular matrix (ECM) disorders), including collagen VI related dystrophies and FKBP14 related kyphoscoliotic type of Ehlers-Danlos syndrome; and 3) various (congenital) myopathies with prominent joint hypermobility including RYR1- and SEPN1-related myopathy. The aim of this review is to assist clinical geneticists and other clinicians with recognition of these disorders.<ovid:br/>Copyright &#xa9; 2015 Wiley Periodicals, Inc.

http://onlinelibrary.wiley.com/journal/10.1002/(ISSN)1552-4876

http://ovidsp.ovid.com/ovidweb.cgi?T=JS&PAGE=reference&D=emed16&NEWS=N&AN=603278975

https://ovidsp.ovid.com/ovidweb.cgi?T=JS&CSC=Y&NEWS=N&PAGE=fulltext&D=emed16&DO=10.1002%2fajmg.c.31433

http://sfxhosted.exlibrisgroup.com/mayo?sid=OVID:embase&id=pmid:25821091&id=doi:10.1002%2Fajmg.c.31433&issn=1552-4868&isbn=&volume=169&issue=1&spage=23&pages=23-42&date=2015&title=American+Journal+of+Medical+Genetics%2C+Part+C%3A+Seminars+in+Medical+Genetics&atitle=The+neuromuscular+differential+diagnosis+of+joint+hypermobility&aulast=Donkervoort&pid=%3Cauthor%3EDonkervoort+S.%3C%2Fauthor%3E&%3CAN%3E603278975%3C%2FAN%3E

**87. Dental problems in a patient with the classic type of Ehlers-Danlos syndrome--a case report**

Osica, P. and A. Janas (2015). "Dental problems in a patient with the classic type of Ehlers-Danlos syndrome--a case report." Developmental period medicine 19(4): 496

EP - 502.

The huge progress in diagnostic and therapeutic procedures in developmental medicine allowed not only to save lives of many children and adolescents, but also enforced the necessity of close cooperation between many specialists. Unfortunately dental treatment is still not an integral part of taking care of disabled children and youth with chronic diseases. The situation worsens, when we come across the so-called rare diseases. Lack of access to dental services, when it comes to disabled patients, results from financial situation, healthcare system, as well as parents' ignorance of necessity of more frequent prophylactic and treatment visits. Whereas the reluctance of dentists towards the care of disabled patients is caused by difficulties with enforcing the recommendations and lack of procedures for taking care of patients with rare diseases, which was repeatedly signalled by us. Such situation is ideally pictured by yet another described case of the 17 year old patient with Ehlers-Danlos syndrome. Despite many dental visits, no vital treatment decisions have been made. In our Department, in the procedures of 1-day surgery, tooth 48, being the cause of pain, has been extracted. Basing on all of the above, it can be deduced that preserving the continuation and consequence in spreading the knowledge of rare diseases among patients, as well as physicians and dentists, is a responsibility of everyone who even once came across this problem.

http://ovidsp.ovid.com/ovidweb.cgi?T=JS&PAGE=reference&D=emed16&NEWS=N&AN=610684094

https://ovidsp.ovid.com/ovidweb.cgi?T=JS&CSC=Y&NEWS=N&PAGE=fulltext&D=emed16&AN=610684094

http://sfxhosted.exlibrisgroup.com/mayo?sid=OVID:embase&id=pmid:26982760&id=doi:&issn=1428-345X&isbn=&volume=19&issue=4&spage=496&pages=496-502&date=2015&title=Developmental+period+medicine&atitle=Dental+problems+in+a+patient+with+the+classic+type+of+Ehlers-Danlos+syndrome--a+case+report&aulast=Osica&pid=%3Cauthor%3EOsica+P.%3C%2Fauthor%3E&%3CAN%3E610684094%3C%2FAN%3E

**88. Towards a re-thinking of the clinical significance of generalized joint hypermobility, joint hypermobiity syndrome, and Ehlers-Danlos syndrome, hypermobility type**

Castori, M., et al. (2014). "Towards a re-thinking of the clinical significance of generalized joint hypermobility, joint hypermobiity syndrome, and Ehlers-Danlos syndrome, hypermobility type." American Journal of Medical Genetics, Part A 164(3): 588

EP - 590.

http://onlinelibrary.wiley.com/journal/10.1002/(ISSN)1552-4833

http://ovidsp.ovid.com/ovidweb.cgi?T=JS&PAGE=reference&D=emed15&NEWS=N&AN=52999800

https://ovidsp.ovid.com/ovidweb.cgi?T=JS&CSC=Y&NEWS=N&PAGE=fulltext&D=emed15&DO=10.1002%2fajmg.a.36437

http://sfxhosted.exlibrisgroup.com/mayo?sid=OVID:embase&id=pmid:24504907&id=doi:10.1002%2Fajmg.a.36437&issn=1552-4825&isbn=&volume=164&issue=3&spage=588&pages=588-590&date=2014&title=American+Journal+of+Medical+Genetics%2C+Part+A&atitle=Towards+a+re-thinking+of+the+clinical+significance+of+generalized+joint+hypermobility%2C+joint+hypermobiity+syndrome%2C+and+Ehlers-Danlos+syndrome%2C+hypermobility+type&aulast=Castori&pid=%3Cauthor%3ECastori+M.%3C%2Fauthor%3E&%3CAN%3E52999800%3C%2FAN%3E

**89. Ehlers-Danlos syndrome - A commonly misunderstood group of conditions**

Sobey, G. (2014). "Ehlers-Danlos syndrome - A commonly misunderstood group of conditions." Clinical Medicine, Journal of the Royal College of Physicians of London 14(4): 432

EP - 436.

All clinicians need to be aware of EDS and its variable presentations that, in some types, can be life threatening. Recognition of EDS can allow accurate diagnosis by genetic testing, genetic counselling and specific, appropriate management and follow-up. &#xa9; Royal College of Physicians 2014. All rights reserved.

http://www.clinmed.rcpjournal.org/content/14/4/432.full.pdf+html

http://ovidsp.ovid.com/ovidweb.cgi?T=JS&PAGE=reference&D=emed15&NEWS=N&AN=373770860

https://ovidsp.ovid.com/ovidweb.cgi?T=JS&CSC=Y&NEWS=N&PAGE=fulltext&D=emed15&DO=10.7861%2fclinmedicine.14-4-432

http://sfxhosted.exlibrisgroup.com/mayo?sid=OVID:embase&id=pmid:25099849&id=doi:10.7861%2Fclinmedicine.14-4-432&issn=1470-2118&isbn=&volume=14&issue=4&spage=432&pages=432-436&date=2014&title=Clinical+Medicine%2C+Journal+of+the+Royal+College+of+Physicians+of+London&atitle=Ehlers-Danlos+syndrome+-+A+commonly+misunderstood+group+of+conditions&aulast=Sobey&pid=%3Cauthor%3ESobey+G.%3C%2Fauthor%3E&%3CAN%3E373770860%3C%2FAN%3E

**90. Re-writing the natural history of pain and related symptoms in the joint hypermobility syndrome/Ehlers-Danlos syndrome, hypermobility type**

Castori, M., et al. (2013). "Re-writing the natural history of pain and related symptoms in the joint hypermobility syndrome/Ehlers-Danlos syndrome, hypermobility type." American Journal of Medical Genetics, Part A 161(12): 2989

EP - 3004.

Joint hypermobility syndrome (JHS) and Ehlers-Danlos syndrome, hypermobility type (EDS-HT) are two clinically overlapping connective tissue disorders characterized by chronic/recurrent pain, joint instability complications, and minor skin changes. Fatigue and headache are also common, although are not yet considered diagnostic criteria. JHS/EDS-HT is a unexpectedly common condition that remains underdiagnosed by most clinicians and pain specialists. This results in interventions limited to symptomatic and non-satisfactory treatments, lacking reasonable pathophysiologic rationale. In this manuscript the fragmented knowledge on pain, fatigue, and headache in JHS/EDS is presented with review of the available published information and a description of the clinical course by symptoms, on the basis of authors' experience. Pathogenic mechanisms are suggested through comparisons with other functional somatic syndromes (e.g., chronic fatigue syndrome, fibromyalgia, and functional gastrointestinal disorders). The re-writing of the natural history of JHS/EDS-HT is aimed to raise awareness among clinical geneticists and specialists treating chronic pain conditions about pain and other complications of JHS/EDS-HT. Symptoms' clustering by disease stage is proposed to investigate both the molecular causes and the symptoms management of JHS/EDS-HT in future studies. &#xa9; 2013 Wiley Periodicals, Inc.

http://ovidsp.ovid.com/ovidweb.cgi?T=JS&PAGE=reference&D=emed14&NEWS=N&AN=52862962

https://ovidsp.ovid.com/ovidweb.cgi?T=JS&CSC=Y&NEWS=N&PAGE=fulltext&D=emed14&DO=10.1002%2fajmg.a.36315

http://sfxhosted.exlibrisgroup.com/mayo?sid=OVID:embase&id=pmid:24254847&id=doi:10.1002%2Fajmg.a.36315&issn=1552-4825&isbn=&volume=161&issue=12&spage=2989&pages=2989-3004&date=2013&title=American+Journal+of+Medical+Genetics%2C+Part+A&atitle=Re-writing+the+natural+history+of+pain+and+related+symptoms+in+the+joint+hypermobility+syndrome%2FEhlers-Danlos+syndrome%2C+hypermobility+type&aulast=Castori&pid=%3Cauthor%3ECastori+M.%3C%2Fauthor%3E&%3CAN%3E52862962%3C%2FAN%3E

**91. Ehlers-Danlos syndrome, hypermobility type: A characterization of the patients' lived experience**

Murray, B., et al. (2013). "Ehlers-Danlos syndrome, hypermobility type: A characterization of the patients' lived experience." American Journal of Medical Genetics, Part A 161(12): 2981

EP - 2988.

Hypermobility type Ehlers-Danlos syndrome (EDS-HT) is an inherited connective tissue disorder clinically diagnosed by the presence of significant joint hypermobility and associated skin manifestations. This article presents a large-scale study that reports the lived experience of EDS-HT patients, the broad range of symptoms that individuals with EDS-HT experience, and the impact these symptoms have on daily functioning. A 237-item online survey, including validated questions regarding pain and depression, was developed. Four hundred sixty-six (466) adults (90% female, 52% college or higher degree) with a self-reported diagnosis of EDS-HT made in a clinic or hospital were included. The most frequently reported symptoms were joint pain (99%), hypermobility (99%), and limb pain (91%). They also reported a high frequency of other conditions including chronic fatigue (82%), anxiety (73%), depression (69%), and fibromyalgia (42%). Forty-six percent of respondents reported constant pain often described as aching and tiring/exhausting. Despite multiple interventions and therapies, many individuals (53%) indicated that their diagnosis negatively affected their ability to work or attend school. Our results show that individuals with EDS-HT can experience a wide array of symptoms and co-morbid conditions. The degree of constant pain and disability experienced by the majority of EDS-HT respondents is striking and illustrates the impact this disorder has on quality of life as well as the clinical challenges inherent in managing this complex connective tissue disorder. &#xa9; 2013 Wiley Periodicals, Inc.

http://ovidsp.ovid.com/ovidweb.cgi?T=JS&PAGE=reference&D=emed14&NEWS=N&AN=52862961

https://ovidsp.ovid.com/ovidweb.cgi?T=JS&CSC=Y&NEWS=N&PAGE=fulltext&D=emed14&DO=10.1002%2fajmg.a.36293

http://sfxhosted.exlibrisgroup.com/mayo?sid=OVID:embase&id=pmid:24254846&id=doi:10.1002%2Fajmg.a.36293&issn=1552-4825&isbn=&volume=161&issue=12&spage=2981&pages=2981-2988&date=2013&title=American+Journal+of+Medical+Genetics%2C+Part+A&atitle=Ehlers-Danlos+syndrome%2C+hypermobility+type%3A+A+characterization+of+the+patients%27+lived+experience&aulast=Murray&pid=%3Cauthor%3EMurray+B.%3C%2Fauthor%3E&%3CAN%3E52862961%3C%2FAN%3E

**92. Children with generalised joint hypermobility and musculoskeletal complaints: state of the art on diagnostics, clinical characteristics, and treatment**

Scheper, M. C., et al. (2013). "Children with generalised joint hypermobility and musculoskeletal complaints: state of the art on diagnostics, clinical characteristics, and treatment." BioMed Research International 2013: 121054.

To provide a state of the art on diagnostics, clinical characteristics, and treatment of paediatric generalised joint hypermobility (GJH) and joint hypermobility syndrome (JHS). A narrative review was performed regarding diagnostics and clinical characteristics. Effectiveness of treatment was evaluated by systematic review. Searches of Medline and Central were performed and included nonsymptomatic and symptomatic forms of GJH (JHS, collagen diseases). In the last decade, scientific research has accumulated on all domains of the ICF. GJH/JHS can be considered as a clinical entity, which can have serious effects during all stages of life. However research regarding the pathological mechanism has resulted in new potential opportunities for treatment. When regarding the effectiveness of current treatments, the search identified 1318 studies, from which three were included (JHS: n = 2, Osteogenesis Imperfecta: n = 1). According to the best evidence synthesis, there was strong evidence that enhancing physical fitness is an effective treatment for children with JHS. However this was based on only two studies. Based on the sparsely available knowledge on intervention studies, future longitudinal studies should focus on the effect of physical activity, fitness, and joint stabilisation. In JHS and chronic pain, the effectiveness of a multidisciplinary approach should be investigated.

http://ovidsp.ovid.com/ovidweb.cgi?T=JS&PAGE=reference&D=emed14&NEWS=N&AN=563033310

https://ovidsp.ovid.com/ovidweb.cgi?T=JS&CSC=Y&NEWS=N&PAGE=fulltext&D=emed14&AN=563033310

http://sfxhosted.exlibrisgroup.com/mayo?sid=OVID:embase&id=pmid:23971021&id=doi:&issn=2314-6141&isbn=&volume=2013&issue=&spage=121054&pages=121054&date=2013&title=BioMed+research+international&atitle=Children+with+generalised+joint+hypermobility+and+musculoskeletal+complaints%3A+state+of+the+art+on+diagnostics%2C+clinical+characteristics%2C+and+treatment&aulast=Scheper&pid=%3Cauthor%3EScheper+M.C.%3C%2Fauthor%3E&%3CAN%3E563033310%3C%2FAN%3E

**93. Children with generalised joint hypermobility and musculoskeletal complaints: State of the art on diagnostics, clinical characteristics, and treatment**

Scheper, M. C., et al. (2013). "Children with generalised joint hypermobility and musculoskeletal complaints: State of the art on diagnostics, clinical characteristics, and treatment." BioMed Research International 2013: 121054.

Introduction. To provide a state of the art on diagnostics, clinical characteristics, and treatment of paediatric generalised joint hypermobility (GJH) and joint hypermobility syndrome (JHS). Method. A narrative review was performed regarding diagnostics and clinical characteristics. Effectiveness of treatment was evaluated by systematic review. Searches of Medline and Central were performed and included nonsymptomatic and symptomatic forms of GJH (JHS, collagen diseases). Results. In the last decade, scientific research has accumulated on all domains of the ICF. GJH/JHS can be considered as a clinical entity, which can have serious effects during all stages of life. However research regarding the pathological mechanism has resulted in new potential opportunities for treatment. When regarding the effectiveness of current treatments, the search identified 1318 studies, from which three were included (JHS: n=2, Osteogenesis Imperfecta: n=1). According to the best evidence synthesis, there was strong evidence that enhancing physical fitness is an effective treatment for children with JHS. However this was based on only two studies. Conclusion. Based on the sparsely available knowledge on intervention studies, future longitudinal studies should focus on the effect of physical activity, fitness, and joint stabilisation. In JHS and chronic pain, the effectiveness of a multidisciplinary approach should be investigated. &#xa9; 2013 M. C. Scheper et al.

http://ovidsp.ovid.com/ovidweb.cgi?T=JS&PAGE=reference&D=emed14&NEWS=N&AN=369562229

https://ovidsp.ovid.com/ovidweb.cgi?T=JS&CSC=Y&NEWS=N&PAGE=fulltext&D=emed14&DO=10.1155%2f2013%2f121054

http://sfxhosted.exlibrisgroup.com/mayo?sid=OVID:embase&id=pmid:23971021&id=doi:10.1155%2F2013%2F121054&issn=2314-6133&isbn=&volume=2013&issue=&spage=121054&pages=&date=2013&title=BioMed+Research+International&atitle=Children+with+generalised+joint+hypermobility+and+musculoskeletal+complaints%3A+State+of+the+art+on+diagnostics%2C+clinical+characteristics%2C+and+treatment&aulast=Scheper&pid=%3Cauthor%3EScheper+M.C.%3C%2Fauthor%3E&%3CAN%3E369562229%3C%2FAN%3E

**94. An update on pediatric bleeding disorders: Bleeding scores, benign joint hypermobility, and platelet function testing in the evaluation of the child with bleeding symptoms**

O'Brien, S. H. (2012). "An update on pediatric bleeding disorders: Bleeding scores, benign joint hypermobility, and platelet function testing in the evaluation of the child with bleeding symptoms." American Journal of Hematology 87(SUPPL. 1): S40

EP - S44.

Evaluating a child with symptoms of easy bruising and/or bleeding remains a challenge in pediatric hematology, and there is no "one size fits all" approach. This review focuses on recent research in three elements of the evaluation of a child with a suspected bleeding disorder. We will first discuss the development of the standardized Pediatric Bleeding Questionnaire, and its applications in research and clinical settings. We will then discuss the relationship between benign hypermobility syndromes and hemostasis, and the importance of including a Beighton Score in the physical examination of any child presenting with unusual bruising or bleeding. While prolonged bleeding times and abnormal platelet aggregation are common findings in children with benign hypermobility, normal coagulation studies do not exclude the presence of a connective tissue disorder in a child presenting with easy bleeding and joint hypermobility on examination. Finally, we will discuss the current state of knowledge regarding the laboratory evaluation of platelet function in children. Platelet function disorders are among the most common inherited bleeding disorders. However, testing for such disorders is time-consuming and requires a step-wise approach. We will review the indications for and limitations of the most commonly utilized platelet function laboratory studies. &#xa9; 2012 Wiley Periodicals, Inc.

http://ovidsp.ovid.com/ovidweb.cgi?T=JS&PAGE=reference&D=emed13&NEWS=N&AN=51937218

https://ovidsp.ovid.com/ovidweb.cgi?T=JS&CSC=Y&NEWS=N&PAGE=fulltext&D=emed13&DO=10.1002%2fajh.23157

http://sfxhosted.exlibrisgroup.com/mayo?sid=OVID:embase&id=pmid:22460356&id=doi:10.1002%2Fajh.23157&issn=0361-8609&isbn=&volume=87&issue=SUPPL.+1&spage=S40&pages=S40-S44&date=2012&title=American+Journal+of+Hematology&atitle=An+update+on+pediatric+bleeding+disorders%3A+Bleeding+scores%2C+benign+joint+hypermobility%2C+and+platelet+function+testing+in+the+evaluation+of+the+child+with+bleeding+symptoms&aulast=O%27Brien&pid=%3Cauthor%3EO%27Brien+S.H.%3C%2Fauthor%3E&%3CAN%3E51937218%3C%2FAN%3E

**95. Muscle-tendon tissue properties in the hypermobility type of Ehlers-Danlos syndrome**

Rombaut, L., et al. (2012). "Muscle-tendon tissue properties in the hypermobility type of Ehlers-Danlos syndrome." Arthritis care & research 64(5): 766

EP - 772.

To investigate the passive properties of the plantar flexors muscle-tendon tissue in patients with the hypermobility type of Ehlers-Danlos syndrome (EDS-HT). Twenty-five women with EDS-HT and 25 sex- and age-matched healthy control subjects participated in the study. Passive resistive torque (PRT) of the plantar flexors was measured with an isokinetic dynamometer during 2 standardized stretch protocols to obtain the passive muscle tension. Protocol 1 consisted of 4 continuous cycles to a predetermined angle of 10degree dorsiflexion. Protocol 2 consisted of a slow stretch to the onset of pain. Torque, angle, and electromyography were simultaneously recorded during the tests. To take muscle thickness into account, muscle cross-sectional area (MCSA) was obtained with peripheral quantitative computed tomography. Stiffness of the Achilles tendon was assessed using a dynamometer in combination with ultrasonography. The results demonstrate a significantly larger maximal joint angle in the EDS-HT patients accompanied by a similar PRT compared to the control subjects (protocol 2), indicating a lower passive muscle tension in the patient group. PRT for the predetermined angle (protocol 1) was the same for both groups and there was no difference in MSCA. Furthermore, a significantly lower Achilles tendon stiffness was seen in the patient group than in the control group. This study is the first to provide evidence for altered passive properties of the muscle-tendon unit in EDS-HT patients. These changes are thought to be associated with structural modifications in connective tissue. Copyright &#xa9; 2012 by the American College of Rheumatology.

http://ovidsp.ovid.com/ovidweb.cgi?T=JS&PAGE=reference&D=emed13&NEWS=N&AN=364820327

https://ovidsp.ovid.com/ovidweb.cgi?T=JS&CSC=Y&NEWS=N&PAGE=fulltext&D=emed13&AN=364820327

http://sfxhosted.exlibrisgroup.com/mayo?sid=OVID:embase&id=pmid:22232076&id=doi:&issn=2151-4658&isbn=&volume=64&issue=5&spage=766&pages=766-772&date=2012&title=Arthritis+care+%26+research&atitle=Muscle-tendon+tissue+properties+in+the+hypermobility+type+of+Ehlers-Danlos+syndrome&aulast=Rombaut&pid=%3Cauthor%3ERombaut+L.%3C%2Fauthor%3E&%3CAN%3E364820327%3C%2FAN%3E

**96. Comprehensive molecular analysis demonstrates type V collagen mutations in over 90% of patients with classic EDS and allows to refine diagnostic criteria**

Symoens, S., et al. (2012). "Comprehensive molecular analysis demonstrates type V collagen mutations in over 90% of patients with classic EDS and allows to refine diagnostic criteria." Human Mutation 33(10): 1485

EP - 1493.

Type V collagen mutations are associated with classic Ehlers-Danlos Syndrome (EDS), but it is unknown for which proportion they account and to what extent other genes are involved. We analyzed COL5A1 and COL5A2 in 126 patients with a diagnosis or suspicion of classic EDS. In 93 patients, a type V collagen defect was found, of which 73 were COL5A1 mutations, 13 were COL5A2 mutations and seven were COL5A1 null-alleles with mutation unknown. The majority of the 73 COL5A1 mutations generated a COL5A1 null-allele, whereas one-third were structural mutations, scattered throughout COL5A1. All COL5A2 mutations were structural mutations. Reduced availability of type V collagen appeared to be the major disease-causing mechanism, besides other intra- and extracellular contributing factors. All type V collagen defects were identified within a group of 102 patients fulfilling all major clinical Villefranche criteria, that is, skin hyperextensibility, dystrophic scarring and joint hypermobility. No COL5A1/COL5A2 mutation was detected in 24 patients who displayed skin and joint hyperextensibility but lacked dystrophic scarring. Overall, over 90% of patients fulfilling all major Villefranche criteria for classic EDS were shown to harbor a type V collagen defect, which indicates that this is the major-if not only-cause of classic EDS. &#xa9; 2012 Wiley Periodicals, Inc.

http://ovidsp.ovid.com/ovidweb.cgi?T=JS&PAGE=reference&D=emed13&NEWS=N&AN=52105026

https://ovidsp.ovid.com/ovidweb.cgi?T=JS&CSC=Y&NEWS=N&PAGE=fulltext&D=emed13&DO=10.1002%2fhumu.22137

http://sfxhosted.exlibrisgroup.com/mayo?sid=OVID:embase&id=pmid:22696272&id=doi:10.1002%2Fhumu.22137&issn=1059-7794&isbn=&volume=33&issue=10&spage=1485&pages=1485-1493&date=2012&title=Human+Mutation&atitle=Comprehensive+molecular+analysis+demonstrates+type+V+collagen+mutations+in+over+90%25+of+patients+with+classic+EDS+and+allows+to+refine+diagnostic+criteria&aulast=Symoens&pid=%3Cauthor%3ESymoens+S.%3C%2Fauthor%3E&%3CAN%3E52105026%3C%2FAN%3E

**97. Chronic fatigue syndrome is commonly diagnosed in patients with Ehlers-Danlos syndrome hypermobility type/joint hypermobility syndrome**

Castori, M., et al. (2011). "Chronic fatigue syndrome is commonly diagnosed in patients with Ehlers-Danlos syndrome hypermobility type/joint hypermobility syndrome." Clinical and Experimental Rheumatology 29(3): 597

EP - 598.

http://ovidsp.ovid.com/ovidweb.cgi?T=JS&PAGE=reference&D=emed12&NEWS=N&AN=362527527

https://ovidsp.ovid.com/ovidweb.cgi?T=JS&CSC=Y&NEWS=N&PAGE=fulltext&D=emed12&AN=362527527

http://sfxhosted.exlibrisgroup.com/mayo?sid=OVID:embase&id=pmid:21722506&id=doi:&issn=0392-856X&isbn=&volume=29&issue=3&spage=597&pages=597-598&date=2011&title=Clinical+and+Experimental+Rheumatology&atitle=Chronic+fatigue+syndrome+is+commonly+diagnosed+in+patients+with+Ehlers-Danlos+syndrome+hypermobility+type%2Fjoint+hypermobility+syndrome&aulast=Castori&pid=%3Cauthor%3ECastori+M.%3C%2Fauthor%3E&%3CAN%3E362527527%3C%2FAN%3E

**98. Need for a consensus on the methods by which to measure joint mobility and the definition of norms for hypermobility that reflect age, gender and ethnic-dependent variation: is revision of criteria for joint hypermobility syndrome and Ehlers-Danlos syndrome hypermobility type indicated?**

Remvig, L., et al. (2011). "Need for a consensus on the methods by which to measure joint mobility and the definition of norms for hypermobility that reflect age, gender and ethnic-dependent variation: is revision of criteria for joint hypermobility syndrome and Ehlers-Danlos syndrome hypermobility type indicated?" Rheumatology (Oxford, England) 50(6): 1169

EP - 1171.

http://ovidsp.ovid.com/ovidweb.cgi?T=JS&PAGE=reference&D=emed12&NEWS=N&AN=560073160

https://ovidsp.ovid.com/ovidweb.cgi?T=JS&CSC=Y&NEWS=N&PAGE=fulltext&D=emed12&DO=10.1093%2frheumatology%2fker140

http://sfxhosted.exlibrisgroup.com/mayo?sid=OVID:embase&id=pmid:21482540&id=doi:10.1093%2Frheumatology%2Fker140&issn=1462-0332&isbn=&volume=50&issue=6&spage=1169&pages=1169-1171&date=2011&title=Rheumatology+%28Oxford%2C+England%29&atitle=Need+for+a+consensus+on+the+methods+by+which+to+measure+joint+mobility+and+the+definition+of+norms+for+hypermobility+that+reflect+age%2C+gender+and+ethnic-dependent+variation%3A+is+revision+of+criteria+for+joint+hypermobility+syndrome+and+Ehlers-Danlos+syndrome+hypermobility+type+indicated%3F&aulast=Remvig&pid=%3Cauthor%3ERemvig+L.%3C%2Fauthor%3E&%3CAN%3E560073160%3C%2FAN%3E

**99. Impact of joint laxity and hypermobility on the musculoskeletal system**

Wolf, J. M., et al. (2011). "Impact of joint laxity and hypermobility on the musculoskeletal system." Journal of the American Academy of Orthopaedic Surgeons 19(8): 463

EP - 471.

Excessive joint laxity, or hypermobility, is a common finding of clinical importance in the management of musculoskeletal conditions. Hypermobility is common in young patients and in general is associated with an increased incidence of musculoskeletal injury. Hypermobility has been implicated in ankle sprains, anterior cruciate ligament injury, shoulder instability, and osteoarthritis of the hand. Patients with hypermobility and musculoskeletal injuries often seek care for diffuse musculoskeletal pain and injuries with no specific inciting event. Orthopaedic surgeons and other healthcare providers should be aware of the underlying relationship between hypermobility and musculoskeletal injury to avoid unnecessary diagnostic tests and inappropriate management. Prolonged therapy and general conditioning are typically required, with special emphasis on improving strength and proprioception to address symptoms and prevent future injury. Orthopaedic surgeons must recognize the implications of joint mobility syndromes in the management and rehabilitation of several musculoskeletal injuries and orthopaedic disorders.

http://ovidsp.ovid.com/ovidweb.cgi?T=JS&PAGE=reference&D=emed12&NEWS=N&AN=362451898

https://ovidsp.ovid.com/ovidweb.cgi?T=JS&CSC=Y&NEWS=N&PAGE=fulltext&D=emed12&DO=10.5435%2f00124635-201108000-00002

http://sfxhosted.exlibrisgroup.com/mayo?sid=OVID:embase&id=pmid:21807914&id=doi:10.5435%2F00124635-201108000-00002&issn=1067-151X&isbn=&volume=19&issue=8&spage=463&pages=463-471&date=2011&title=Journal+of+the+American+Academy+of+Orthopaedic+Surgeons&atitle=Impact+of+joint+laxity+and+hypermobility+on+the+musculoskeletal+system&aulast=Wolf&pid=%3Cauthor%3EWolf+J.M.%3C%2Fauthor%3E&%3CAN%3E362451898%3C%2FAN%3E

**100. Atypical symptom of Ehlers-Danlos syndrome impeding diagnosis: Feeling of spinal instability**

Gasik, R. and T. Styczynski (2009). "Atypical symptom of Ehlers-Danlos syndrome impeding diagnosis: Feeling of spinal instability." Journal of Rheumatology 36(8): 1847

EP - 1848.

http://jrheum.org/content/36/8/1847.full.pdf+html

http://ovidsp.ovid.com/ovidweb.cgi?T=JS&PAGE=reference&D=emed11&NEWS=N&AN=355110057

https://ovidsp.ovid.com/ovidweb.cgi?T=JS&CSC=Y&NEWS=N&PAGE=fulltext&D=emed11&DO=10.3899%2fjrheum.081146

http://sfxhosted.exlibrisgroup.com/mayo?sid=OVID:embase&id=pmid:19671830&id=doi:10.3899%2Fjrheum.081146&issn=0315-162X&isbn=&volume=36&issue=8&spage=1847&pages=1847-1848&date=2009&title=Journal+of+Rheumatology&atitle=Atypical+symptom+of+Ehlers-Danlos+syndrome+impeding+diagnosis%3A+Feeling+of+spinal+instability&aulast=Gasik&pid=%3Cauthor%3EGasik+R.%3C%2Fauthor%3E&%3CAN%3E355110057%3C%2FAN%3E

**101. Desmopressin responsiveness in children with Ehlers-Danlos syndrome associated bleeding symptoms**

Mast, K. J., et al. (2009). "Desmopressin responsiveness in children with Ehlers-Danlos syndrome associated bleeding symptoms." British Journal of Haematology 144(2): 230

EP - 233.

Ehlers-Danlos Syndrome (EDS) is caused by heritable collagen defects and may be associated with bleeding symptoms. Desmopressin has been described in case reports to decrease bleeding times in these patients. This study sought to assess bleeding time responsiveness to desmopressin therapy in a cohort of children with EDS-associated bleeding manifestations. A retrospective chart review of children with EDS referred for bleeding symptoms was utilized. Twenty-six children were included; 19 (73%) had a desmopressin challenge. The mean bleeding time was 11.26 (+/-4.39) min, decreasing to 5.95 (+/-2.41) min with treatment (P < 0.01). Desmopressin normalizes bleeding times in children with EDS. &#xa9; 2008 The Authors.

http://ovidsp.ovid.com/ovidweb.cgi?T=JS&PAGE=reference&D=emed11&NEWS=N&AN=354017304

https://ovidsp.ovid.com/ovidweb.cgi?T=JS&CSC=Y&NEWS=N&PAGE=fulltext&D=emed11&DO=10.1111%2fj.1365-2141.2008.07446.x

http://sfxhosted.exlibrisgroup.com/mayo?sid=OVID:embase&id=pmid:19036109&id=doi:10.1111%2Fj.1365-2141.2008.07446.x&issn=0007-1048&isbn=&volume=144&issue=2&spage=230&pages=230-233&date=2009&title=British+Journal+of+Haematology&atitle=Desmopressin+responsiveness+in+children+with+Ehlers-Danlos+syndrome+associated+bleeding+symptoms&aulast=Mast&pid=%3Cauthor%3EMast+K.J.%3C%2Fauthor%3E&%3CAN%3E354017304%3C%2FAN%3E

**102. Oral manifestations of Ehlers-Danlos syndrome and presentation of a case**

Pourshahidi, S., et al. (2009). "Oral manifestations of Ehlers-Danlos syndrome and presentation of a case." Iranian Red Crescent Medical Journal 11(2): 206

EP - 209.

Ehlers-Danlos syndrome (EDS) is a rare syndrome, primarily diagnosed by clinical findings and family history. The clinical manifestations of EDS in the orofacial region consist of extra-oral and intra-oral manifestations. Its prognosis depends on the (sub) type and the proper, early diagnosis of the syndrome. The purpose of the present paper is to discuss the manifestations of this syndrome, especially its oral manifestations, and to present a case. &#xa9; Iranian Red Crescent Medical Journal.

http://www.irmj.ir/browse.php?a_code=A-10-12-17&slc_lang=en&sid=1&ftxt=1

http://ovidsp.ovid.com/ovidweb.cgi?T=JS&PAGE=reference&D=emed11&NEWS=N&AN=354802774

https://ovidsp.ovid.com/ovidweb.cgi?T=JS&CSC=Y&NEWS=N&PAGE=fulltext&D=emed11&AN=354802774

http://sfxhosted.exlibrisgroup.com/mayo?sid=OVID:embase&id=pmid:&id=doi:&issn=2074-1804&isbn=&volume=11&issue=2&spage=206&pages=206-209&date=2009&title=Iranian+Red+Crescent+Medical+Journal&atitle=Oral+manifestations+of+Ehlers-Danlos+syndrome+and+presentation+of+a+case&aulast=Pourshahidi&pid=%3Cauthor%3EPourshahidi+S.%3C%2Fauthor%3E&%3CAN%3E354802774%3C%2FAN%3E

**103. The differential diagnosis of children with joint hypermobility: A review of the literature**

Tofts, L. J., et al. (2009). "The differential diagnosis of children with joint hypermobility: A review of the literature." Pediatric Rheumatology 7: 1.

Background: In this study we aimed to identify and review publications relating to the diagnosis of joint hypermobility and instability and develop an evidence based approach to the diagnosis of children presenting with joint hypermobility and related symptoms. <ovid:br/>Method(s): We searched Medline for papers with an emphasis on the diagnosis of joint hypermobility, including Heritable Disorders of Connective Tissue (HDCT). <ovid:br/>Result(s): 3330 papers were identified: 1534 pertained to instability of a particular joint; 1666 related to the diagnosis of Ehlers Danlos syndromes and 330 related to joint hypermobility. There are inconsistencies in the literature on joint hypermobility and how it relates to and overlaps with milder forms of HDCT. There is no reliable method of differentiating between Joint Hypermobility Syndrome, familial articular hypermobility and Ehlers-Danlos syndrome (hypermobile type), suggesting these three disorders may be different manifestations of the same spectrum of disorders. We describe our approach to children presenting with joint hypermobility and the published evidence and expert opinion on which this is based. <ovid:br/>Conclusion(s): There is value in identifying both the underlying genetic cause of joint hypermobility in an individual child and those hypermobile children who have symptoms such as pain and fatigue and might benefit from multidisciplinary rehabilitation management. Every effort should be made to diagnose the underlying disorder responsible for joint hypermobility which may only become apparent over time. We recommend that the term "Joint Hypermobility Syndrome" is used for children with symptomatic joint hypermobility resulting from any underlying HDCT and that these children are best described using both the term Joint Hypermobility Syndrome and their HDCT diagnosis. &#xa9; 2009 Tofts et al; licensee BioMed Central Ltd.

http://ovidsp.ovid.com/ovidweb.cgi?T=JS&PAGE=reference&D=emed11&NEWS=N&AN=354236742

https://ovidsp.ovid.com/ovidweb.cgi?T=JS&CSC=Y&NEWS=N&PAGE=fulltext&D=emed11&DO=10.1186%2f1546-0096-7-1

http://sfxhosted.exlibrisgroup.com/mayo?sid=OVID:embase&id=pmid:&id=doi:10.1186%2F1546-0096-7-1&issn=1546-0096&isbn=&volume=7&issue=1&spage=1&pages=&date=2009&title=Pediatric+Rheumatology&atitle=The+differential+diagnosis+of+children+with+joint+hypermobility%3A+A+review+of+the+literature&aulast=Tofts&pid=%3Cauthor%3ETofts+L.J.%3C%2Fauthor%3E&%3CAN%3E354236742%3C%2FAN%3E

**104. Joint hypermobility as a distinctive feature in the differential diagnosis of myopathies**

Voermans, N. C., et al. (2009). "Joint hypermobility as a distinctive feature in the differential diagnosis of myopathies." Journal of Neurology 256(1): 13

EP - 27.

Congenital and adultonset inherited myopathies represent a wide spectrum of syndromes. Classification is based upon clinical features and biochemical and genetic defects. Joint hypermobility is one of the distinctive clinical features that has often been underrecognized so far. We therefore present an overview of myopathies associated with joint hypermobility: Ullrich congenital muscular dystrophy, Bethlem myopathy, congenital muscular dystrophy with joint hyperlaxity, multi-minicore disease, central core disease, and limb girdle muscular dystrophy 2E with joint hyperlaxity and contractures. We shortly discuss a second group of disorders characterised by both muscular features and joint hypermobility: the inherited disorders of connective tissue Ehlers-Danlos syndrome and Marfan syndrome. Furthermore, we will briefly discuss the extent and pattern of joint hypermobility in these myopathies and connective tissue disorders and propose two grading scales commonly used to score the severity of joint hypermobility. We will conclude focusing on the various molecules involved in these disorders and on their role and interactions in muscle and tendon, with a view to further elucidate the pathophysiology of combined hypermobility and myopathy. Hopefully, this review will contribute to enhanced recognition of joint hypermobility and thus be of aid in differential diagnosis. &#xa9; 2009 Steinkopff-Verlag.

http://ovidsp.ovid.com/ovidweb.cgi?T=JS&PAGE=reference&D=emed11&NEWS=N&AN=50427135

https://ovidsp.ovid.com/ovidweb.cgi?T=JS&CSC=Y&NEWS=N&PAGE=fulltext&D=emed11&DO=10.1007%2fs00415-009-0105-1

http://sfxhosted.exlibrisgroup.com/mayo?sid=OVID:embase&id=pmid:19221853&id=doi:10.1007%2Fs00415-009-0105-1&issn=0340-5354&isbn=&volume=256&issue=1&spage=13&pages=13-27&date=2009&title=Journal+of+Neurology&atitle=Joint+hypermobility+as+a+distinctive+feature+in+the+differential+diagnosis+of+myopathies&aulast=Voermans&pid=%3Cauthor%3EVoermans+N.C.%3C%2Fauthor%3E&%3CAN%3E50427135%3C%2FAN%3E

**105. Dysphonia-A rare early symptom of Ehlers-Danlos syndrome?**

Rimmer, J., et al. (2008). "Dysphonia-A rare early symptom of Ehlers-Danlos syndrome?" International Journal of Pediatric Otorhinolaryngology 72(12): 1889

EP - 1892.

Ehlers-Danlos syndrome is a connective tissue disorder characterized by joint hypermobility, skin hyperextensibility and cutaneous fragility. It usually presents in young adults and is rarely diagnosed in children. Voice involvement in young children, to our knowledge, has not been reported in the literature. We present two cases with dysphonia from birth and an eventual diagnosis of Ehlers-Danlos syndrome. The syndrome and its relevance to voice pathology are discussed. We suggest that Ehlers-Danlos syndrome should be considered as an underlying diagnosis in atypical presentations of dysphonia in young children. &#xa9; 2008 Elsevier Ireland Ltd. All rights reserved.

http://ovidsp.ovid.com/ovidweb.cgi?T=JS&PAGE=reference&D=emed10&NEWS=N&AN=50314997

https://ovidsp.ovid.com/ovidweb.cgi?T=JS&CSC=Y&NEWS=N&PAGE=fulltext&D=emed10&DO=10.1016%2fj.ijporl.2008.09.018

http://sfxhosted.exlibrisgroup.com/mayo?sid=OVID:embase&id=pmid:18976821&id=doi:10.1016%2Fj.ijporl.2008.09.018&issn=0165-5876&isbn=&volume=72&issue=12&spage=1889&pages=1889-1892&date=2008&title=International+Journal+of+Pediatric+Otorhinolaryngology&atitle=Dysphonia-A+rare+early+symptom+of+Ehlers-Danlos+syndrome%3F&aulast=Rimmer&pid=%3Cauthor%3ERimmer+J.%3C%2Fauthor%3E&%3CAN%3E50314997%3C%2FAN%3E

**106. Differential diagnosis of muscular hypotonia in infants: The kyphoscoliotic type of Ehlers-Danlos syndrome (EDS VI)**

Yis, U., et al. (2008). "Differential diagnosis of muscular hypotonia in infants: The kyphoscoliotic type of Ehlers-Danlos syndrome (EDS VI)." Neuromuscular Disorders 18(3): 210

EP - 214.

The kyphoscoliotic type of Ehlers-Danlos syndrome (EDS VI) (OMIM 225400) is an inherited connective tissue disorder characterized by hypotonia and kyphoscoliosis at birth, joint hypermobility, and skin hyperelasticity and fragility. Biochemically, it is characterized by a deficiency of collagen lysyl hydroxylase (EC 1.14.11.4) due to mutations in PLOD1. This deficiency results in underhydroxylation of collagen lysyl residues and, hence, an abnormal pattern of lysyl pyridinoline (LP) and hydroxylysyl pyridinoline (HP) crosslinks excreted in the urine. Because of hypotonia and delay in gross motor development, a neuromuscular disease is usually suspected, and in most cases the diagnosis is considered only very late, after performing an invasive neuromuscular work-up with normal results. We report a 12-month-old boy with kyphoscoliosis and delayed gross motor development, in whom the differential diagnosis of kyphoscoliotic type of Ehlers-Danlos syndrome (EDS VI) was initially suspected and successively confirmed by the abnormal urinary ratio of total pyridinolines (LP to HP), and by mutation analysis. We advocate the analysis of urinary pyridinolines in all infants with severe hypotonia which is highly specific and sensitive, quick and inexpensive. &#xa9; 2007 Elsevier B.V. All rights reserved.

http://ovidsp.ovid.com/ovidweb.cgi?T=JS&PAGE=reference&D=emed10&NEWS=N&AN=50023607

https://ovidsp.ovid.com/ovidweb.cgi?T=JS&CSC=Y&NEWS=N&PAGE=fulltext&D=emed10&DO=10.1016%2fj.nmd.2007.11.006

http://sfxhosted.exlibrisgroup.com/mayo?sid=OVID:embase&id=pmid:18155911&id=doi:10.1016%2Fj.nmd.2007.11.006&issn=0960-8966&isbn=&volume=18&issue=3&spage=210&pages=210-214&date=2008&title=Neuromuscular+Disorders&atitle=Differential+diagnosis+of+muscular+hypotonia+in+infants%3A+The+kyphoscoliotic+type+of+Ehlers-Danlos+syndrome+%28EDS+VI%29&aulast=Yis&pid=%3Cauthor%3EYis+U.%3C%2Fauthor%3E&%3CAN%3E50023607%3C%2FAN%3E

**107. Ehlers-Danlos syndrome - 20 Years experience with diagnosis and classification**

Proske, S., et al. (2006). "Ehlers-Danlos syndrome - 20 Years experience with diagnosis and classification." JDDG - Journal of the German Society of Dermatology 4(4): 308

EP - 318.

Background: The Ehlers-Danlos syndrome encompasses a group of hereditary disorders of the connective tissue, characterized by hyperextensible skin, joint hypermobility; and varying degrees of vessel and tissue fragility. The main forms are classical, hypermobile, vascular, kyphoscoliotic A/B, arthrochalasis A/B and dermatosparaxis types. <ovid:br/>Patients and Methods: We report our experience in diagnosis and classification of Ehlers-Danlos-syndrome, especially with the combination of clinical and morphological criteria, at the Department of Dermatology of the University of Heidelberg with more than 600 patients between 1984 and 2004. <ovid:br/>Result(s): We classified those types of EDS which are characterized by regular and characteristic ultrastructural changes in the dermal components, primarily collagen, including the classical, hypermobile and vascular types as well as the less-common arthrochalasis and dermatosparaxis types. The combination of clinical and morphologic features facilitates the selection of candidate genes for molecular genetic investigation. <ovid:br/>Conclusion(s): Besides the skin, skeleton and vessels, many other organ systems such as eyes and intestine, can be affected in Ehlers-Danlos syndrome. Accordingly, interdisciplinary cooperation (pediatrics, surgery, orthopedics, rheumatology, neurology, genetics) is necessary. As the connective tissue of the skin is accessible for biopsy and diagnostic investigation, dermatologists should be trained in the diagnostic approach and classification of this syndrome. &#xa9; Blackwell Verlag GmbH.

http://ovidsp.ovid.com/ovidweb.cgi?T=JS&PAGE=reference&D=emed9&NEWS=N&AN=44139131

https://ovidsp.ovid.com/ovidweb.cgi?T=JS&CSC=Y&NEWS=N&PAGE=fulltext&D=emed9&DO=10.1111%2fj.1610-0387.2006.05958.x

http://sfxhosted.exlibrisgroup.com/mayo?sid=OVID:embase&id=pmid:16638060&id=doi:10.1111%2Fj.1610-0387.2006.05958.x&issn=1610-0379&isbn=&volume=4&issue=4&spage=308&pages=308-318&date=2006&title=JDDG+-+Journal+of+the+German+Society+of+Dermatology&atitle=Ehlers-Danlos-Syndrom+-+20+Jahre+erfahrungen+in+diagnostik+und+klassifikation+an+der+Universitats-Hautklinik+Heidelberg&aulast=Proske&pid=%3Cauthor%3EProske+S.%3C%2Fauthor%3E&%3CAN%3E44139131%3C%2FAN%3E

**108. Benign joint hypermobility syndrome: Evaluation, diagnosis, and management**

Simpson, M. R. (2006). "Benign joint hypermobility syndrome: Evaluation, diagnosis, and management." Journal of the American Osteopathic Association 106(9): 531

EP - 536.

Benign joint hypermobility syndrome (BJHS) is a connective tissue disorder with hypermobility in which musculoskeletal symptoms occur in the absence of systemic rheumatologic disease. Although BJHS has been well recognized in the rheumatology and orthopedic literature, it has not been discussed in the family medicine literature. Because most patients with musculoskeletal complaints are first seen by family physicians, it behooves primary care physicians to be familiar with recognizing and diagnosing BJHS. When patients with this syndrome are first seen by a physician, their chief complaint is joint pain, so BJHS can be easily overlooked and not considered in the differential diagnosis. Use of the Brighton criteria facilitates the diagnosis of BJHS. Treatment modalities include patient education, activity modification, stretching and strengthening exercises for the affected joint, and osteopathic manipulative treatment.

http://www.jaoa.org/cgi/reprint/106/9/531.pdf

http://ovidsp.ovid.com/ovidweb.cgi?T=JS&PAGE=reference&D=emed9&NEWS=N&AN=44704011

https://ovidsp.ovid.com/ovidweb.cgi?T=JS&CSC=Y&NEWS=N&PAGE=fulltext&D=emed9&AN=44704011

http://sfxhosted.exlibrisgroup.com/mayo?sid=OVID:embase&id=pmid:17079522&id=doi:&issn=0098-6151&isbn=&volume=106&issue=9&spage=531&pages=531-536&date=2006&title=Journal+of+the+American+Osteopathic+Association&atitle=Benign+joint+hypermobility+syndrome%3A+Evaluation%2C+diagnosis%2C+and+management&aulast=Simpson&pid=%3Cauthor%3ESimpson+M.R.%3C%2Fauthor%3E&%3CAN%3E44704011%3C%2FAN%3E

**109. Joint hypermobility syndrome in childhood. A not so benign multisystem disorder?**

Adib, N., et al. (2005). "Joint hypermobility syndrome in childhood. A not so benign multisystem disorder?" Rheumatology 44(6): 744

EP - 750.

Objectives. Joint hypermobility (JH) or 'ligamentous laxity' is felt to be an underlying risk factor for many types of musculoskeletal presentation in paediatrics, and joint hypermobility syndrome (JHS) describes such disorders where symptoms become chronic, often more generalized and associated with functional impairment. Clinical features are felt to have much in common with more severe disorders, including Ehlers-Danlos syndrome (EDS), osteogenesis imperfecta and Marfan syndrome, although this has not been formally studied in children. We defined the clinical characteristics of all patients with joint hypermobility-related presentations seen from 1999 to 2002 in a tertiary referral paediatric rheumatology unit. Methods. Patients were identified and recruited from paediatric rheumatology clinic and ward, and a dedicated paediatric rheumatology hypermobility clinic at Great Ormond Street Hospital. Data were collected retrospectively on the patients from the paediatric rheumatology clinics (1999-2002) and prospectively on patients seen in the hypermobility clinic (2000-2002). Specifically, historical details of developmental milestones, musculoskeletal or soft tissue diagnoses and symptoms, and significant past medical history were recorded. Examination features sought included measurements of joint and soft tissue laxity, and associated conditions such as scoliosis, dysmorphic features, cardiac murmurs and eye problems. Results. One hundred and twenty-five children (64 females) were included on whom sufficient clinical data could be identified and who had clinical problems ascribed to JH present for longer than 3 months. Sixty-four were from the paediatric rheumatology clinic and 61 from the hypermobility clinic. No differences were found in any of the measures between the two populations and results are presented in a combined fashion. Three-quarters of referrals came from paediatricians and general practitioners but in only 10% was hypermobility recognized as a possible cause of joint complaint. The average age at onset of symptoms was 6.2 yr and age at diagnosis 9.0 yr, indicating a 2- to 3-yr delay in diagnosis. The major presenting complaint was arthralgia in 74%, abnormal gait in 10%, apparent joint deformity 10% and back pain in 6%. Mean age at first walking was 15.0 months; 48% were considered 'clumsy' and 36% as having poor coordination in early childhood. Twelve per cent had 'clicky' hips at birth and 4% actual congenital dislocatable hip. Urinary tract infections were present in 13 and 6% of the female and male cases, respectively. Thirteen and 14%, respectively, had speech and learning difficulties diagnosed. A history of recurrent joint sprains was seen in 20% and actual subluxation/dislocation of joints in 10%. Forty per cent had experienced problems with handwriting tasks, 48% had major limitations of school-based physical education activities, 67% other physical activities and 41% had missed significant periods of schooling because of symptoms. Forty-three per cent described a history of easy bruising. Examination revealed that 94% scored >=4/9 on the Beighton scale for generalized hypermobility, with knees (92%), elbows (87%), wrists (82%), hand metacarpophalangeal joints (79%), and ankles (75%) being most frequently involved. Conclusions. JHS is poorly recognized in children with a long delay in the time to diagnosis. Although there is a referral bias towards joint symptoms, a surprisingly large proportion is associated with significant neuromuscular and motor development problems. Our patients with JHS also show many overlap features with genetic disorders such as EDS and Marfan syndrome. The delay in diagnosis results in poor control of pain and disruption of normal home life, schooling and physical activities. Knowledge of the diagnosis and simple interventions are likely to be highly effective in reducing the morbidity and cost to the health and social services. &#xa9; The Author 2005. Published by Oxford University Press on behalf of the British Society for Rheumatology. All rights reserved.

http://ovidsp.ovid.com/ovidweb.cgi?T=JS&PAGE=reference&D=emed9&NEWS=N&AN=41487202

https://ovidsp.ovid.com/ovidweb.cgi?T=JS&CSC=Y&NEWS=N&PAGE=fulltext&D=emed9&DO=10.1093%2frheumatology%2fkeh557

http://sfxhosted.exlibrisgroup.com/mayo?sid=OVID:embase&id=pmid:15728418&id=doi:10.1093%2Frheumatology%2Fkeh557&issn=1462-0324&isbn=&volume=44&issue=6&spage=744&pages=744-750&date=2005&title=Rheumatology&atitle=Joint+hypermobility+syndrome+in+childhood.+A+not+so+benign+multisystem+disorder%3F&aulast=Adib&pid=%3Cauthor%3EAdib+N.%3C%2Fauthor%3E&%3CAN%3E41487202%3C%2FAN%3E

**110. The clinical presentation of Ehlers-Danlos syndrome**

Lawrence, E. J. (2005). "The clinical presentation of Ehlers-Danlos syndrome." Advances in Neonatal Care 5(6): 301

EP - 314.

Ehlers-Danlos syndrome (EDS), a heterogeneous group of inheritable connective tissue disorders, is attributed to mutations in connective tissue genes. These mutations cause defects in collagen. Collagen, a connective tissue protein that acts like glue, gives strength to the body and provides support and elasticity for movement. Thus, the altered gene affects the mechanical properties of skin, joints, ligaments, and blood vessels. Ehlers-Danlos syndrome is transmitted through autosomal dominant, autosomal recessive, or x-linked patterns of inheritance. The life expectancy of an affected infant varies with the type of EDS. This article provides an overview of the 6 major classifications of EDS, their unique clinical presentations, a focused physical assessment guide, considerations for nursing care, and resources for parents. Ehlers-Danlos syndrome can be a potentially debilitating syndrome. It requires preventative and protective measures starting at birth to preserve joint function to improve infant outcomes. Caring for patients with EDS requires an understanding of the potential associated complications to help minimize the physical and emotional impact of the syndrome and improve the quality of life for affected individuals. Copyright &#xa9; 2005 by The National Association of Neonatal Nurses.

http://ovidsp.ovid.com/ovidweb.cgi?T=JS&PAGE=reference&D=emed9&NEWS=N&AN=41773456

https://ovidsp.ovid.com/ovidweb.cgi?T=JS&CSC=Y&NEWS=N&PAGE=fulltext&D=emed9&DO=10.1016%2fj.adnc.2005.09.006

http://sfxhosted.exlibrisgroup.com/mayo?sid=OVID:embase&id=pmid:16338669&id=doi:10.1016%2Fj.adnc.2005.09.006&issn=1536-0903&isbn=&volume=5&issue=6&spage=301&pages=301-314&date=2005&title=Advances+in+Neonatal+Care&atitle=The+clinical+presentation+of+Ehlers-Danlos+syndrome&aulast=Lawrence&pid=%3Cauthor%3ELawrence+E.J.%3C%2Fauthor%3E&%3CAN%3E41773456%3C%2FAN%3E

**111. Prevalence of temporomandibular joint dysfunction in Ehlers-Danlos syndromes**

De Coster, P. J., et al. (2004). "Prevalence of temporomandibular joint dysfunction in Ehlers-Danlos syndromes." Orthodontics & craniofacial research 7(4): 237

EP - 241.

In 2018, the Ministry of Health and Social Services (MoHSS) introduced acetic acid visual inspection (AAVI) screening program and treatment of cervical precancers with cryotherapy in replacement of Pap smear intervention in Namibia. The study examines the effective use of AAVI-and-cryotherapy treatment on HPV-diagnosed women. Female patients of the reproductive age (20-49 years) group visiting 2 Namibian healthcare facilities for AAVI and cryotherapy treatment were investigated using a cross-sectional approach. The SPSS and coding themes were used to analyze data received through questionnaires and face-to-face interviews from a total of 265 participants. Written informed consent was obtained from participants for treatment and for publication. Among the women that participated in the study, 151 (62%) tested HPV positive, of which 132 (53%) were referred for cryotherapy and 19 (8%) for colposcopy treatments due to Cervical Intraepithelial Neoplasia (CIN I-III) detected. Regardless of the age groups, there was significant evidence of an association between para gravida and HPV positive (chi(2)(6) = 24.518; P < .001) and HPV negative (chi(2)(18) = 137.098; P < .001). About 80% of all participants experienced unpleasant pelvic sensations during the examination, with slight abdominal pain during and after the procedure. Ten percent experienced brownish discharges from their pelvis, of which was treated during the cryotherapy treatment. These findings suggest that MoHSS should actively re-evaluate the existing policies to promote AAVI and cryotherapy treatment.

http://ovidsp.ovid.com/ovidweb.cgi?T=JS&PAGE=reference&D=emed8&NEWS=N&AN=39679498

https://ovidsp.ovid.com/ovidweb.cgi?T=JS&CSC=Y&NEWS=N&PAGE=fulltext&D=emed8&AN=39679498

http://sfxhosted.exlibrisgroup.com/mayo?sid=OVID:embase&id=pmid:15562587&id=doi:&issn=1601-6335&isbn=&volume=7&issue=4&spage=237&pages=237-240%3B+author+reply+240-241&date=2004&title=Orthodontics+%26+craniofacial+research&atitle=Prevalence+of+temporomandibular+joint+dysfunction+in+Ehlers-Danlos+syndromes&aulast=De+Coster&pid=%3Cauthor%3EDe+Coster+P.J.%3C%2Fauthor%3E&%3CAN%3E39679498%3C%2FAN%3E

**112. Connective Tissue Disorders with Spontaneous Spinal Cerebrospinal Fluid Leaks and Intracranial Hypotension: A Prospective Study**

Schievink, W. I., et al. (2004). "Connective Tissue Disorders with Spontaneous Spinal Cerebrospinal Fluid Leaks and Intracranial Hypotension: A Prospective Study." Neurosurgery 54(1): 65

EP - 71.

OBJECTIVE: Intracranial hypotension attributable to a spontaneous spinal cerebrospinal fluid (CSF) leak is an increasingly recognized cause of postural headaches. The cause of these leaks is poorly understood, but it is likely multifactorial and may involve a primary connective tissue disorder. We undertook a study to estimate the contribution of systemic connective tissue disorders to the development of spontaneous spinal CSF leaks. <ovid:br/>METHOD(S): We examined a group of 18 consecutive patients with spontaneous spinal CSF leaks for features of a connective tissue disorder. <ovid:br/>RESULT(S): The mean age of the 15 female patients and 3 male patients was 38 years (range, 22-55 yr). Seven patients (38%) demonstrated stigmata of a systemic connective tissue disorder, and three distinct types of disorders could be identified, as follows. 1) The association of spontaneous spinal CSF leaks and minor skeletal features of Marfan syndrome was noted for three patients. 2) Ehlers-Danlos syndrome Type 11 was noted for two patients. 3) Joint hypermobility associated with marked attenuation of the dorsal muscular fascia, precluding proper wound closure, was noted for two patients. In addition, isolated small-joint hypermobility was observed for five patients (28%). Slit-lamp ocular examinations, echocardiographic evaluations, histopathological examinations of skin biopsy specimens, and renal scanning did not reveal any other features of a systemic connective tissue disorder. <ovid:br/>CONCLUSION(S): Findings suggesting connective tissue disorders are common among patients with spontaneous spinal CSF leaks, and manifestations may be subtle. A variety of disorders can be identified, probably reflecting genetic heterogeneity. Problems with wound healing may occur as a result of the systemic nature of the underlying connective tissue disorder.

http://ovidsp.ovid.com/ovidweb.cgi?T=JS&PAGE=reference&D=emed8&NEWS=N&AN=38040858

https://ovidsp.ovid.com/ovidweb.cgi?T=JS&CSC=Y&NEWS=N&PAGE=fulltext&D=emed8&DO=10.1227%2f01.NEU.0000097200.18478.7B

http://sfxhosted.exlibrisgroup.com/mayo?sid=OVID:embase&id=pmid:14683542&id=doi:10.1227%2F01.NEU.0000097200.18478.7B&issn=0148-396X&isbn=&volume=54&issue=1&spage=65&pages=65-71&date=2004&title=Neurosurgery&atitle=Connective+Tissue+Disorders+with+Spontaneous+Spinal+Cerebrospinal+Fluid+Leaks+and+Intracranial+Hypotension%3A+A+Prospective+Study&aulast=Schievink&pid=%3Cauthor%3ESchievink+W.I.%3C%2Fauthor%3E&%3CAN%3E38040858%3C%2FAN%3E

**113. Reduced skin thickness: A new minor diagnostic criterion for the classical and hypermobility types of Ehlers-Danlos syndrome**

Eisenbeiss, C., et al. (2003). "Reduced skin thickness: A new minor diagnostic criterion for the classical and hypermobility types of Ehlers-Danlos syndrome." British Journal of Dermatology 149(4): 850

EP - 852.

Background: The diagnosis of Ehlers-Danlos syndrome (EDS) is mainly based on clinical criteria, although in some instances a sound molecular diagnosis is available. Clinical signs can be divided into two categories: one with high diagnostic specificity and the other with low specificity. Despite the fact that reduced skin thickness is one of the dermatological features in patients with EDS, this issue has not been analysed in greater detail. <ovid:br/>Objective(s): To determine skin thickness in patients with the classical and the hypermobility types of EDS. <ovid:br/>Method(s): In 21 patients with classical type of EDS and in nine patients with hypermobility type of EDS, skin thickness was analysed at different body sites by cross-sectional b-mode scans obtained with a 20-MHz ultrasound system. <ovid:br/>Result(s): We found a significant decrease in skin thickness in both types of EDS, which was highest at the chest and at the distal part of the lower leg. <ovid:br/>Conclusion(s): We propose that the reduced thickness of the dermis as determined by high-resolution 20-MHz ultrasound can be used as a new minor criterion in the diagnosis of the classical and the hypermobility types of EDS.

http://ovidsp.ovid.com/ovidweb.cgi?T=JS&PAGE=reference&D=emed8&NEWS=N&AN=37412008

https://ovidsp.ovid.com/ovidweb.cgi?T=JS&CSC=Y&NEWS=N&PAGE=fulltext&D=emed8&DO=10.1046%2fj.1365-2133.2003.05439.x

http://sfxhosted.exlibrisgroup.com/mayo?sid=OVID:embase&id=pmid:14616379&id=doi:10.1046%2Fj.1365-2133.2003.05439.x&issn=0007-0963&isbn=&volume=149&issue=4&spage=850&pages=850-852&date=2003&title=British+Journal+of+Dermatology&atitle=Reduced+skin+thickness%3A+A+new+minor+diagnostic+criterion+for+the+classical+and+hypermobility+types+of+Ehlers-Danlos+syndrome&aulast=Eisenbeiss&pid=%3Cauthor%3EEisenbeiss+C.%3C%2Fauthor%3E&%3CAN%3E37412008%3C%2FAN%3E

**114. Prevalence of aortic root dilation in the Ehlers-Danlos syndrome**

Wenstrup, R. J., et al. (2002). "Prevalence of aortic root dilation in the Ehlers-Danlos syndrome." Genetics in Medicine 4(3): 112

EP - 117.

Purpose: To determine the prevalence of proximal aortic abnormalities in patients with Ehlers-Danlos syndrome (EDS). <ovid:br/>Method(s): In a prospective cohort study, aortic measurements by two-dimensional echocardiography were performed on consecutive EDS patients. <ovid:br/>Result(s): Twenty-eight percent (20 of 71) had aortic root dilation (ARD) (> +2 SD above population based norms). Fourteen of 42 individuals with the classical form of EDS (types I/II) and 6 of 29 individuals with the hypermobile form (type III) had ARD, with no gender differences. <ovid:br/>Conclusion(s): ARD is a common finding in EDS. Longitudinal studies are indicated to determine progression of ARD and its clinical significance.

http://ovidsp.ovid.com/ovidweb.cgi?T=JS&PAGE=reference&D=emed7&NEWS=N&AN=44698569

https://ovidsp.ovid.com/ovidweb.cgi?T=JS&CSC=Y&NEWS=N&PAGE=fulltext&D=emed7&DO=10.1097%2f00125817-200205000-00003

http://sfxhosted.exlibrisgroup.com/mayo?sid=OVID:embase&id=pmid:&id=doi:10.1097%2F00125817-200205000-00003&issn=1098-3600&isbn=&volume=4&issue=3&spage=112&pages=112-117&date=2002&title=Genetics+in+Medicine&atitle=Prevalence+of+aortic+root+dilation+in+the+Ehlers-Danlos+syndrome&aulast=Wenstrup&pid=%3Cauthor%3EWenstrup+R.J.%3C%2Fauthor%3E&%3CAN%3E44698569%3C%2FAN%3E

**115. Wrist problems in patients with Ehlers-Danlos syndrome**

Woerdeman, L. A. E., et al. (2000). "Wrist problems in patients with Ehlers-Danlos syndrome." European Journal of Plastic Surgery 23(4): 208

EP - 210.

Ehlers-Danlos syndrome (EDS) is a group of hereditary disorders involving collagen synthesis. One hundred patients with EDS were mailed a general questionnaire, containing a detailed section concerning their wrists. Fifty-six percent of patients returned the questionnaire, of which 70% had wrist complaints. Sixteen out of 39 patients with complaints had undergone surgery for these complaints, all with eventual relief of the symptoms. All 18 patients with EDS type III had severe wrist complaints and all of them were unable to work because of these complaints, at a relatively young age. Ten of these patients visited our clinic and all had a severe midcarpal instability both clinically and on cinematography All X-rays were normal. This finding should prompt clinicians to make an early diagnosis of EDS with appropriate counseling for future jobs and hobbies.

https://www.springer.com/journal/238

http://ovidsp.ovid.com/ovidweb.cgi?T=JS&PAGE=reference&D=emed27&NEWS=N&AN=30330385

https://ovidsp.ovid.com/ovidweb.cgi?T=JS&CSC=Y&NEWS=N&PAGE=fulltext&D=emed27&DO=10.1007%2fs002380050252

http://sfxhosted.exlibrisgroup.com/mayo?sid=OVID:embase&id=pmid:&id=doi:10.1007%2Fs002380050252&issn=0930-343X&isbn=&volume=23&issue=4&spage=208&pages=208-210&date=2000&title=European+Journal+of+Plastic+Surgery&atitle=Wrist+problems+in+patients+with+Ehlers-Danlos+syndrome&aulast=Woerdeman&pid=%3Cauthor%3EWoerdeman+L.A.E.%3C%2Fauthor%3E&%3CAN%3E30330385%3C%2FAN%3E

**116. Ehlers-Danlos syndrome type VII: Clinical features and molecular defects**

Giunta, C., et al. (1999). "Ehlers-Danlos syndrome type VII: Clinical features and molecular defects." Journal of Bone and Joint Surgery 81(2): 225

EP - 238.

We evaluated the clinical features, molecular defects, and problems associated with the management of two patients who had type-VII Ehlers- Danlos syndrome and reviewed the cases of eighteen patients with this condition who had been reported on previously. The typical clinical features associated with this syndrome include bilateral congenital dislocation of the hip; severe generalized hypermobility of the joints; multiple dislocations of joints other than the hip; muscular hypotonia; and hyperelasticity, fragility, and a doughy texture of the skin. Collagen and DNA analyses demonstrated that both of our patients had type-VIIB Ehlers-Danlos syndrome, which is caused by heterozygous new mutations of the COL1A2 gene that encodes the proalpha2(I) chain of type-I procollagen. The obligatory GT dinucleotide at the splice donor site of intron 6 was altered in both of our patients: one patient (Case 1) had an A substitution of the G nucleotide, and the other patient (Case 2) had a C substitution of the T nucleotide. Abnormal splicing resulted in the loss of the exon 6-encoded N-telopeptide, which includes the N-proteinase cleavage site. Despite multiple operative procedures, one of our patients, who was thirty-seven years old at the time of the most recent follow-up, continued to have persistent subluxation of the right hip and osteoarthritis of the left hip. Closed reduction of the dislocated hips, regardless of the type of immobilization used, was unsuccessful in all twenty patients. The results of open reduction were improved when capsulorrhaphy was combined with lilac or femoral osteotomy, or both.

https://journals.lww.com/jbjsjournal/pages/default.aspx

http://ovidsp.ovid.com/ovidweb.cgi?T=JS&PAGE=reference&D=emed6&NEWS=N&AN=29124083

https://ovidsp.ovid.com/ovidweb.cgi?T=JS&CSC=Y&NEWS=N&PAGE=fulltext&D=emed6&DO=10.2106%2f00004623-199902000-00010

http://sfxhosted.exlibrisgroup.com/mayo?sid=OVID:embase&id=pmid:10073586&id=doi:10.2106%2F00004623-199902000-00010&issn=0021-9355&isbn=&volume=81&issue=2&spage=225&pages=225-238&date=1999&title=Journal+of+Bone+and+Joint+Surgery&atitle=Ehlers-Danlos+syndrome+type+VII%3A+Clinical+features+and+molecular+defects&aulast=Giunta&pid=%3Cauthor%3EGiunta+C.%3C%2Fauthor%3E&%3CAN%3E29124083%3C%2FAN%3E

**117. Hypermobility syndrome**

Everman, D. B. and N. H. Robin (1998). "Hypermobility syndrome." Pediatrics in review / American Academy of Pediatrics 19(4): 111

EP - 117.

http://ovidsp.ovid.com/ovidweb.cgi?T=JS&PAGE=reference&D=emed6&NEWS=N&AN=128254882

https://ovidsp.ovid.com/ovidweb.cgi?T=JS&CSC=Y&NEWS=N&PAGE=fulltext&D=emed6&DO=10.1542%2fpir.19-4-111

http://sfxhosted.exlibrisgroup.com/mayo?sid=OVID:embase&id=pmid:9557061&id=doi:10.1542%2Fpir.19-4-111&issn=0191-9601&isbn=&volume=19&issue=4&spage=111&pages=111-117&date=1998&title=Pediatrics+in+review+%2F+American+Academy+of+Pediatrics&atitle=Hypermobility+syndrome&aulast=Everman&pid=%3Cauthor%3EEverman+D.B.%3C%2Fauthor%3E&%3CAN%3E128254882%3C%2FAN%3E

**118. Peripheral neuropathy in Ehlers-Danlos syndrome**

Galan, E. and B. G. Kousseff (1995). "Peripheral neuropathy in Ehlers-Danlos syndrome." Pediatric Neurology 12(3): 242

EP - 245.

Two unrelated male patients with clinical manifestations of Ehlers-Danlos syndrome type III and peripheral neuropathy are presented. At age 13 years, one developed bilateral brachial plexus palsy unrelated to trauma and 2 years later, a right lumbosacral plexopathy. The other presented at age 3 years with a left brachial plexopathy after sustaining a fracture of the lateral condyle of the right humerus. In both patients, nerve conduction velocities demonstrated conduction block across the brachial plexus and recovery was incomplete, indicating that peripheral neuropathy is a serious complication of Ehlers-Danlos syndrome. Its prompt diagnosis facilitates the care of patients with this syndrome. Increased ligament laxity/stretchability and mechanical trauma may play an important role in the pathogenesis of the neuropathy.

http://ovidsp.ovid.com/ovidweb.cgi?T=JS&PAGE=reference&D=emed5&NEWS=N&AN=25146159

https://ovidsp.ovid.com/ovidweb.cgi?T=JS&CSC=Y&NEWS=N&PAGE=fulltext&D=emed5&DO=10.1016%2f0887-8994%252895%252900003-X

http://sfxhosted.exlibrisgroup.com/mayo?sid=OVID:embase&id=pmid:7619192&id=doi:10.1016%2F0887-8994%252895%252900003-X&issn=0887-8994&isbn=&volume=12&issue=3&spage=242&pages=242-245&date=1995&title=Pediatric+Neurology&atitle=Peripheral+neuropathy+in+Ehlers-Danlos+syndrome&aulast=Galan&pid=%3Cauthor%3EGalan+E.%3C%2Fauthor%3E&%3CAN%3E25146159%3C%2FAN%3E

**119. Ehlers-Danlos syndrome identified from periodontal findings: case report**

Bond, P. J., et al. (1993). "Ehlers-Danlos syndrome identified from periodontal findings: case report." Pediatric dentistry 15(3): 212

EP - 213.

Metabolic syndrome (MS) is a complex disorder characterized by a group of metabolic abnormalities. In the present study, the case of an 18‑year‑old male who presented with MS characteristics with central obesity (overweight and a waist circumference of 95 cm) and dyslipidemia (high TG, low HDL levels and low apoA‑I/apoB‑100) was reported. The patient's family has maternally inherited diabetes and a number of the patient's maternal relatives present MS features. For the investigation of the mitochondrial DNA variants in the patient and the patient's family, genomic DNA of all the family members were extracted from peripheral blood using routine methods. Amplification of mitochondrial DNA in 24 overlapping fragments by PCR, direct sequencing and denaturing high‑performance liquid chromatography was utilized for genetic analysis. Sequences were compared to the reference sequence to identify variants. Bioinformatic methods and databases were used to analyze conservation of the variants and to predict the protein secondary structure. With the exception of the patient, five relatives were diagnosed with MS. Moreover, 5 of the 8 family members had been diagnosed with diabetes, hearing loss and mild kidney impairment according to serum biochemical analysis. Further molecular genetic analysis indicated that the MS‑associated variant T16189C was detected in this family. Notably, a heteroplasmic mutation A8890G was detected in the patient in the mitochondrial ATP6 gene, which codes the ATP synthase subunit 6 (ATPase6). A8890G changed the highly conserved ATPase6 Lys122 into Glu122 in the mitochondrial inner membrane. However, this mutation was not detected in other family members. In conclusion, the mutation A8890G may affect the function of ATPase 6 and the production of ATP, thus contributing to juvenile‑onset MS. It was not detected in other family members possibly due to the mitochondrial genetic segregation or production of a new germline mutation in the juvenile‑onset patient.

http://ovidsp.ovid.com/ovidweb.cgi?T=JS&PAGE=reference&D=emed5&NEWS=N&AN=23921547

https://ovidsp.ovid.com/ovidweb.cgi?T=JS&CSC=Y&NEWS=N&PAGE=fulltext&D=emed5&AN=23921547

http://sfxhosted.exlibrisgroup.com/mayo?sid=OVID:embase&id=pmid:8378161&id=doi:&issn=0164-1263&isbn=&volume=15&issue=3&spage=212&pages=212-213&date=1993&title=Pediatric+dentistry&atitle=Ehlers-Danlos+syndrome+identified+from+periodontal+findings%3A+case+report&aulast=Bond&pid=%3Cauthor%3EBond+P.J.%3C%2Fauthor%3E&%3CAN%3E23921547%3C%2FAN%3E
